# Supplementary material for: Predictive Biomarkers for Outcomes of Immune Checkpoint Inhibitors (ICIs) in Melanoma: A Systematic Review
Source: Cancers (Basel). 2021 Dec 18;13(24):6366. doi: 10.3390/cancers13246366 (PMC8699321; doi:10.3390/cancers13246366)
Supplement: Supplementary file 1 [file cancers-13-06366-s001.zip › cancers-1505001-supplementary.pdf]

Supplementary Appendix

Predictive biomarkers for outcomes of immune checkpoint inhibitors (ICIs) in melanoma: a systematic review

Cancers (submitted in 2021) – J. C. Baltussen et al

TABLE OF CONTENTS

Table S1 - Study characteristics of all included reports and quality assessment ……2
Table S2 – Outcomes and interpretation of all included reports ……19
Table S3 - Peripheral blood biomarkers that were studied for anti-CLTA-4 therapy ……41
Table S4 - Peripheral blood biomarkers that were studied for anti-PD-1 therapy ……44
Table S5 - Peripheral blood biomarkers that were studied for mixed cohorts……46
Table S6 - Peripheral blood biomarkers that were studied for combination therapy ……47
Table S7 - Tumor biomarkers that were studied for anti-CLTA-4 therapy ……48
Table S8 - Tumor biomarkers that were studied for anti-PD-1 therapy ……50
Table S9 - Tumor biomarkers that were studied for mixed cohorts ……51
Table S10 - Tumor biomarkers that were studied for combination therapy ……52
Table S11 – Fecal biomarkers that were studied for all ICIs ……54
Complete search stragety……54
Reference list of all included studies……55

Table S1 - Study characteristics of all included reports in systematic review with most important extracted data and quality assessment

Paper	Study design	N Melanoma patients	Type of melanoma	Biomarker of interest	Tumor (T) or blood (B)	Type of test	Timing of biomarker	Study phase	Adjustment for relevant confounders	Median age (range)	Median follow-up	Type of treatment	Risk of bias	Quality assessment					
														Participation	Attrition	Prognostic	Outcome	Adjustment	Statistics
Amaral et. al. Cancers 2020 [1]	Prospective cohort (Germany)	59	Uveal 15.3%, unknown 8.5%, acral 10.2%, cutaneous 59%, mucosal 5.7%	Pathologic or likely pathologic (P/LP) germline mutations, TMB, LDH, s100B	T	DNA sequencing in blood and formalin-fixed paraffin-embedded blocks	P	D	LDH, TMB	61 years, IQR 51-72	13 months	Ipilimumab + nivolumab	M	M	M	L	L	M	L
Arce Vargas et. al, Cancer Cell, 2019 **[2]**	Data from 3 cohorts (Finland/UK)	51	NR	Germline DNA-mutations CD16a-V158F, CD32a-H131R, SNPs in association w. TMB	T	Germline DNA sequencing	P	D	N/A	NR	NR	Ipilimumab	H	H	H	L	H	H	H
Ascierto et. al, J. Immunoth. Cancer, 2019 **[3]**	Retrospective cohort	71	Cutaneous 73%, mucosal 7%, SPI 7% uveal 7 NR 7%	BDX008 (proteomic test), LDH, NLR	B	Proteomic test, developed by machine learning	P	D	Mutation status, line of treatment and LDH	59 (28-86) in BDX008+ 66 (32-80) in BD008- patients	29.7 versus 6.8 months in BDX008+	Anti-PD1 (in different lines)	M	L	M	M	L	L	L
Auslander et. Al, Nature Medicine 2018 **[4]**	Data selected from 3 datasets (US)	41	NR	Immuno-predictive score (IMPRES based on transcriptomics)	T	Transcriptomics	P	D	N/A	NR	NR	Anti-PD1 (31), anti-CTLA4 (10)	M	M	M	L	M	H	H
Ayers et. al, J. Clin. Investigation, 2017 **[5]**	Data from 3 trials	81	NR	GEP including IFN-y-10-gene signature and expanded immune signature	T	Nano String, using RNA from baseline tumour samples	P	D + V	Cancer type, performance status	NR	NR	Pembrolizumab	M	M	M	L	L	H	H
Babacic et. al, J. Immunoth.Cancer2020 **[6]**	Prospective cohort (Sweden)	24	NR	Plasma proteomics, including PD-L1	B	Mass-spectrometry for global proteomics + proximity extension assays for targeted proteomics	P + D	D	N/A	72 (SD 15.81)	809 days (responders), 125 days non-responders	Anti-PD1 compared to MAPKI	M	H	M	L	L	H	M
Balatoni et. al, Pathol. Oncol. Res. 2020 **[7]**	Retrospective cohort (Hungary)	47	Skin, ocular or mucosal, treated with second line immunotherapy	LDH, ESR,ALC, ANC, AEC + TILs in tumour	T	Automatic cell counting+ semiquantitative analysis of HE-stained slides	P + D	D	Gender, age, stage, PS, disease load, LDH, previous treatment	57 (26–83)	9.8 months	Ipilimumab	M	M	H	L	L	L	M
Balatoni et. al, Cancer Immun. Immunotherapy, 2018 **[8]**	Retrospective cohort (Hungary)	30	Skin melanoma	Monoclonal antibodies against CD8, CD20cy, CD45RO, CD68, CD16, CD137, CD13, FOXP3, NKp46, CD4, PD-1 + LDH	T	Immunohisto-chemistry	P	D	Immune cell densities, age, gender, stage, PS, organs involved, LDH, previous treatments	Responders 67 (51–78) Non-responders 53 (30–66)	NR	Ipilimumab	M	L	M	L	L	L	H
Barak et. al, Anticancer Res, 2015 **[9]**	NR (Israeli cohort)	137	NR	S-100β	B	ELISA essay	P + D	D	N/A	NR	NR	Anti-BRAF, ipi, anti- PD1	H	H	H	L	H	H	H

Paper	Study design	N Melanoma patients	Type of melanoma	Biomarker of interest	Tumor (T) or blood (B)	Type of test	Timing of biomarker	Study phase	Adjustment for relevant confounders	Median age (range)	Median follow-up	Type of treatment	Quality assessment						
													Risk of bias	Participation	Attrition	Prognostic	Outcome	Adjustment	Statistics
Bartlett et. al, Cancer, 2020 **[10]**	Retrospective analysis of prospective database (US)	224	Cutaneous, mucosal, unknown primary	NLR	B	Peripheral complete blood count with differentiation	P + D	D	Type of melanoma, number of metastatic sites	65.5 (24-94)	39 months	Pembroli-zumab or nivolumab	L	L	L	L	L	L	L
Bence et. al, JEAVD, 2020 **[11]**	Retrospective cohort (France)	51	Cutaneous metastatic melanoma (stage III/IV)	PD-L1, CD8+ Tumor-infiltrating lymphocytes, CD28, Ki67	T	Immunohisto-chemistry	P	D	Age, histological type, ulceration and pTNM stage	Mean 60.55 (26-83)	Maximum of 60 months	Anti-PD1 or CTLA-4 inhibitors	M	M	H	M	L	H	M
Bjoern et. al, Oncoimmunology, 2016 **[12]**	Prospective cohort (Denmark)	40	NR	ALC, T-cells, CD27, CD45RA, CCR7, HLA-DR. MDSC's, cytokines, LAG-3, TIM-3, PD-1. NK	B	Flow cytometry, cytokine measurements in serum	P + D	D	N/A	67.2 (35-83)	NR	Ipilimumab	M	M	M	M	L	H	H
Bochem et. al, Plos One, 2019 **[13]**	Cohort (Germany)	75	NR	CD56, PD1	B	Flow cytometry using PBMCs	P	D	M-category, LDH, CMV-serostatus, sex, age	73 (IQR 62-79)	Nearly 2 years (709 days)	Anti-PD1	M	M	M	L	L	M	M
Bruggemann et. al, J Cancer Res Clin Oncol 2017 **[14]**	Retrospective multi-center case (Germany / Switserland)	111	NR	PD-L1 on mRNA level	T	Quantitative RT-PCF after extracting mRNA from formalin-fixed paraffin tissue	P	D	Lymph node metastases	Mean 61 (42-82) for responders and 62 (30-86) for NR	At least 3 months of follow-up available	Ipilimumab	M	L	L	L	M	H	M
Cabrita et. al, Nature, 2020 **[15]**	Retrospective manually selected form 3 cohorts (Denmark+ other)	186	NR	Constructed gene signature of tertiary lymphoid structures	T	Gene expression Profiling tumour/ RNA sequence data from the TCGA	P	D	TMB,	61(24-90)	NR	Anti-CTLA-4 or anti-PD-1	M	M	H	L	L	H	M
Campesato et. al, Oncotarget, 2015 **[16]**	Retrospective cohort (Brazil)	64	NR	Cancer gene panels (CGP)- mutational load	T	CGP-mutational load from WES mutation data, extracted from DNA	D	D	N/A	NR	At least 6 months of follow up	Ipilimumab	H	H	H	L	H	H	M
Capone et. al, J.Immunother. Cancer, 2018 **[17]**	Retrospective cohort (Italy)	97	NR	WBC, ALC, ANC, LDH, NLR, derived NLR	B	Peripheral blood count	P	D	LDH	61 (21– 85)	At least 6 months of follow up	Nivolumab	M	L	M	L	H	M	M
Capone et. al, J. Transl. Medicine 2020 **[18]**	Cohort (Italy)	100	NR	Peripheral CD8+T cells expressing CD73	B	Flow cytometry using PBMCs	P	D	NR	62 (28–90)	11 months	Nivolumab	M	L	M	L	H	M	L
Carlino et. al, European J. Cancer 2018 **[19]**	Prospective RCT	833	All except for ocular melanoma	PD-L1 expression	T	Dako PD-L1 IHC 22C3 pharmDx assay, with a cut-off of >1% PD-L1 expression	P	D	N/A	PDL+ 64.5 (18-89), 63 (18-88) PDL- 58.5 (19-86) and 58 (18-86)	24 months	Pembrolizumab or ipilimumab	M	M	L	M	L	H	H
Cassidy et. al, EBioMedicine 2017 **[20]**	Retrospective study of pro-	97	Cutaneous 171, ocular 13, mucosal 13	NLR	B	Peripheral blood count	P + D	D	M-stage, gender, melanoma type	62.6 (9.8, 91)	54.3 months in IPI group (5–109)	Ipilimumab	L	L	L	L	L	M	L

Paper	Study design	N Melanoma patients	Type of melanoma	Biomarker of interest	Tumor (T) or blood (B)	Type of test	Timing of biomarker	Study phase	Adjustment for relevant confounders	Median age (range)	Median follow-up	Type of treatment	Quality assessment						
													Risk of bias	Participation	Attrition	Prognostic	Outcome	Adjustment	Statistics
	spective database (US)																		
Chakravarti et. al, Melanoma Res, 2017 **[21]**	Cohort (US)	81	Cutaneous 65, mucosal 7, uveal 2, unknown 7	CTLA-4, FasL, PD-1, PD-L1, BRAF-V600E, phospho-Akt, phospho-S6, LDH	T	Avidin–biotin–peroxidase complex method on formalin-fixed parrafrin-embedded tumour	P	D	N/A	NR	At least 24 weeks	Ipilimumab	M	M	M	L	L	M	M
Chasseuil et. al, Acta Dermat. Venereology, 2018 **[22]**	Retrospective cohort (France)	87	Mucosal 11%, choroidal 3% unknown primary 11%	WBC, ALC. leukocyte/ lymphocyte ratio, ANC, NLR, MC, AEC, LDH, CRP.	B	Peripheral blood count	P	D	LDH and CRP	71 (27–92)	227 days	Nivolumab	M	L	M	L	L	M	L
Chat et. al, Cancer Immun. Immunother. 2019 **[23]**	Retrospective (US)	436	NR	25 SNPs associated with autoimmune diseases	B	SNPs from genomic DNA in blood usig Sequenom MassArray System	P	D	N/A	62.85 (19-90.20)	At least 13 weeks	Anti-CLTA-4 Ab N=215, anti-PD1 Ab N=176, ipi/nivo; N=45.	M	H	M	L	L	H	L
Chen et. al, Nature, 2018 **[24]**	Prospective selected from biobank (US)	44	NR	Exosomal circulating PD-L1, Total PD-L1, microvescicle PD-L1	B	Flow cytometry, ultra-centrifugation, exosome isolation	P + D	D	N/A	64 (29-89)	NR	Pembroli-zumab	H	H	H	L	M	H	H
Chen et. al, Frontiers Immunol. 2019 **[25]**	Retrospective selected from WES cohort	332	NR	Lipoprotein receptor-related protein 1B mutation	T	Whole exome sequencing	P	D	Age, sex, stage, TMB	NR	NR	NR	M	M	H	L	M	L	H
Chen et. al, Oncoimmunology 2019 **[26]**	Retrospective selected from 6 databases	332	NR	TMB and several specific mutations; neoantigen load	T	Whole exome sequencing	P	D	Age, gender, stage and hypermutation status	NR	NR	Anti-PD1 Ab and CTLA4 monotherapy	H	L	M	H	M	H	H
Chen et. al, Cancer Discovery, 2016 **[27]**	Prospective cohort (US)	53	Cutaneous 70%, acral 12%, mucosal 2%, unknown 16%	Immune cell infiltrates and GEP	T	12-marker immunohisto-chemistry, nanostring	P + D	D	N/A	57 (27-86)	NR	Anti-CTLA4 Ab, after progression anti-PD1 Ab	M	L	L	L	L	H	M
Chew et. al, Developmental Cell, 2019 **[28]**	Retrospectively selected from 2 chorts	80	NR	DUX4, an embryonic transcription factor	T	RNA-sequencing	P	D	N/A	NR	At least 1 year	Anti-CLTA-4 and anti-PD-1 Ab	H	H	H	L	H	M	H
Cho et. al, Invest. New Drugs 2016 **[29]**	Retrospectively selected (Korea)	37	Acral 17, mucosal 9, other type cutaneous 11	PD-L1 immunostaining status	T	Anti-PD-L1 immunohistoche mistry on formalin-fixed, paraffined tissues	P	D	N/A	58 (21–80)	6.4 months (1.4–11.2)	Pembroli-zumab or nivolumab	H	H	H	L	L	H	L
Chow et. al, Immunity 2019 **[30]**	Prospective manually selected (US)	28	NR	CXCL9 and CXCL10 (CXCR3 chemokines)	B	ELISA + flow cytometry	P + D	D	N/A	Mean NR, only age per patient	42 days	Anti-PD1+ both anti PD1 / anti- CTLA-4	H	H	H	L	L	H	M

Paper	Study design	N Melanoma patients	Type of melanoma	Biomarker of interest	Tumor (T) or blood (B)	Type of test	Timing of biomarker	Study phase	Adjustment for relevant confounders	Median age (range)	Median follow-up	Type of treatment	Quality assessment						
													Risk of bias	Participation	Attrition	Prognostic	Outcome	Adjustment	Statistics
Chowell et. al, Science 2018 **[31]**	Retrospective cohort of prospective databases (US)	419	NR	HLA-I genotype	T	Next-generation sequencing tumor data with targeted gene panel + HLA typing assay	P	D	TMB,	NR	NR	Anti-CTLA-4 or anti-PD-1 therapy	M	M	H	L	M	H	M
Cordonnier et. al, J Extracell Vesicles, 2020 **[32]**	Prospective cohort (France)	100	cutaneous (84%), stage 4 (74%)	circulating exosomal-PD-L1	B	Enzyme-linked immunosorbent assay (ELISA)	P + D	D	N/A	64 (SD 13.7)	16 months	43% anti-PD-1, 10% ipi, 18% BRAF/ MEK inhibitors	M	M	H	L	L	H	L
Cristescu et. al, Science, 2018 **[33]**	Not clearly described, most likely retrospective (US)	89	NR	TMB, T cell–inflamed GEP: CCL5, CD27 -274,- 276CD8, CMKLR1, CXCL9, CXCR6, HLA, IDO1, LAG3, NKG7, PDCD1LG2 PSMB10, STAT1,TIGIT.	T	DNA sequencing (WES) and RNA analysis (gene expression profiling)	P	D	N/A	60 (30 - 88)	NR	Pembroli-zumab	H	H	H	L	L	H	M
Daud et. al, J. Clinical Invest, 2016 **[34]**	Prospective cohort (2 cohorts of 20 pts, US)	40	NR	CD45, CD3, CD4, CD8, FOXP3 + D-1, PD-L1, CTLA-4, and MHC class II	T	Multiparameter flow cytometry	P	D + V	N/A	NR	NR	Anti–PD-1 monotherapy	H	H	M	L	L	H	H
Daud et. al, J. Clin. Oncol. 2016 **[35]**	Keynote 001 study cohort (US, Canada, Australia, France)	451	NR	PD-L1 expression	T	Immunohisto-chemistry assay (22C3 antibody) using MEL scale	P + D	D	N/A	62 (18-94)	NR	Pembroli-zumab	M	L	L	L	L	H	M
de Coana et. al, Oncotarget, 2017 **[36]**	RCT (6 patients) + cohort (37) (Sweden)	43	61% cutaneous, 23% unknown, 9% mucosal, 7% uveal	moMSDCs and CD8 memory T cells	B	Flow cytometry + peripheral blood count	P + D	D	N/A	<65 years = 23, > 65 years = 20	Range 45 and 227 weeks	Ipilimumab	H	H	M	L	L	H	H
Del Re et. al, British J. Cancer 2018 **[37]**	Prospective cohort (Italy)	18	n/a	PD-L1 mRNA expression in exosomes	B	Digital droplet PCR	P + D	D	N/A	71 (45-87)	NR	Nivolumab and pembrolizumab	H	M	H	L	L	H	H
Delyon et. al, Annals Oncol. 2013 **[38]**	Prospective cohort (France)	73	Cutaneous 73%, Uveal 1% mucosal 15%, unknown 11%	Lymphocyte and eosinophil counts, LDH	B	Peripheral blood count	P + D	D	Number of ipilimumab courses	Mean 58.5 (19–86)	At least 12 weeks	Ipilimumab	M	L	H	L	L	H	L
Di Giacomo et. al, Cancer Immun. Immunoth. 2013 **[39]**	Retrospective cohort (Italy)	27	Cutaneous 85%, Uveal 11%, mucosal 4%	ICOS expression on CD4 and CD8 Tcells, ratio of ANC and ALC	B	Flow cytometry	P + D	D	N/A	55 (23–77)	9.6 months	Ipilimumab	M	M	M	L	L	H	L
Diem et. al, Cancer Immun. Immunoth. 2018 **[40]**	Retrospective selected trial patients (Switserland)	9	1 mucosal, 8 cutaneous	CD3,CD4 and CD8 expression on TILs + matched lymph node metastasis	T	Immunohisto-chemistry of formalin-fixed paraffin-embedded full tissues sections	P	D	N/A	63	NR	Ipilimumab	H	H	M	L	L	H	H

Paper	Study design	N Melanoma patients	Type of melanoma	Biomarker of interest	Tumor (T) or blood (B)	Type of test	Timing of biomarker	Study phase	Adjustment for relevant confounders	Median age (range)	Median follow-up	Type of treatment	Quality assessment						
													Risk of bias	Participation	Attrition	Prognostic	Outcome	Adjustment	Statistics
Diem et. al, 2015 European J. Cancer **[41]**	Retrospective cohort (UK)	134	Ocular 11%, acral 3%, cutaneous 69%, mucosal 6%	NLR, albumin, LDH, ECOG PS, BMI,	B	Peripheral blood count	P + D	D	N/A	Mean 58.8 (SD 15)	16.1 months	Ipilimumab	M	L	L	M	L	L	M
Diem et. al, British J. Cancer 2016 **[42]**	Retrospective cohort (UK)	66	NR	LDH	B	Peripheral blood count	P + D	D	N/A	56.2 (IQR 49.1, 66.8)	9 months (95% CI, 6.7–15.4)	Pembrolizumab or nivolumab	M	L	L	M	L	H	M
Diem et. al, J. Immunother. 2019 **[43]**	Prospective cohort (Switserland)	49	Mucosal 14%, uveal 12%	Immunoglobulin G and Subclasses (IgG1, IgG2, IgG3, and IgG4)	B	Immunoturbidimetric methods using Behring nephelometer II	P	D	N/A	68 (30-93)	20.7 months (IQR 17.5–24.3 mo)	Nivo, pembro, ipi or combination nivo+ ipi	L	M	L	L	L	H	L
Dupuis et. al, British J. Cancer 2018 **[44]**	Retrospective cohort (France)	70	Cutaneous	PD-L1+ tumor cells PD-L1+ cells, CD163+ histiocytes, TILs + distribution pattern.	T	Immunolabeling on formalin-fixed, paraffin-embedded (FFPE) tumour block	P	D	N/A	NR	19.3 months (95% CI 16.2–21.4)	Pembrolizumab or nivolumab	H	L	H	L	L	H	H
Edwards et. al, Clin. Cancer Res. 2018 **[45]**	Manually selected (Australia)	13	Cutaneous or unknown primary, stage III	Tumor-resident CD8+ T cells	T	Multiparameter flow cytometry + quantitative multiplex immunofluorescence	P	D	N/A	NR	At least 12 weeks	Pembrolizumab or nivolumab	H	H	H	L	L	H	L
Failing et. al, Melanoma Res. 2017 **[46]**	Retrospective cohort (US)	133	NR	Lymphocyte-to-monocyte ratio	B	Peripheral blood counts	P	D	ANC, Stage, LDH, Brain metastases, PS	61 (18–90)	12 months (0.5–54)	Pembrolizumab	L	L	L	M	L	L	L
Fairfax et. al, Nature Medicine 2020 **[47]**	Retrospective cohort (UK)	55 + 69	Cutaneous	Peripheral CD8+ T cell characteristics	B	Differential expression analysis on CD8+ cell expression profiles + PCR	P + D	D	Type of treatment and age	NR	NR	Anti-PD-1 alone or combined with anti-CTLA-4	H	H	H	L	L	M	H
Fässler et. al, J Immunother. Cancer 2019 **[48]**	2 prospective cohorts (Switzerland)	41	NR	gp100, MelanA/MART1, TRP1/ TYRP1, TRP2/TYPR2 and NY-ESO-1.	B	ELISA assays	P + D	D	N/A	median/mean NR (52-88)	Cohort 1: at least 9-12 weeks, 2: at least 4-6 weeks	Anti-PD1, anti-CTLA4, combination treatment	H	H	H	L	H	H	M
Felix et. al, Intern. Immunopharmacology 2016 **[49]**	Prospective cohort (France)	77	NR	Anti-MICA antibodies, LDH, s100B, MIA	B	ELISA assays	P + D	D	N/A	mean 60.4 (SD 17.4)	28 months	Anti-CTLA-4 Ab	L	L	L	L	L	M	L
Felix et. al, Oncoimmunology 2015 **[50]**	Prospective cohort (France)	77	NR	ALC, CD4, CD8, T-bet, CD122, HLA-DR, granzyme B, CD28, chemokine receptor expression	B	Flow cytometry	P + D	D	N/A	mean 59.8 (SD 16.6)	28 months	Anti-CTLA-4 Ab	M	L	M	L	L	H	M
Ferrucci et. al, Annals Oncol. 2016 **[51]**	Prospective cohort (Italy)	720	Cutaneous 74%, uveal 10%, mucosal 8%	Baseline neutrophils and derived NLR	B	Complete blood count	P	D	Age, ECOG, brain mets, LDH, line of therapy, liver mets	61 (17-88)	16.5 months	Ipilimumab	L	L	L	L	L	L	L

Paper	Study design	N Melanoma patients	Type of melanoma	Biomarker of interest	Tumor (T) or blood (B)	Type of test	Timing of biomarker	Study phase	Adjustment for relevant confounders	Median age (range)	Median follow-up	Type of treatment	Quality assessment						
													Risk of bias	Participation	Attrition	Prognostic	Outcome	Adjustment	Statistics
Ferrucci et. al, British J. Cancer 2015 **[52]**	Retrospective cohort (Italy)	187	Cutaneous 78%, uveal 7%, mucosal 10%	NLR, WBC, ANC, ALC, LDH	B	Complete blood count	P + D	D + V	Age, sex, LDH and ECOG	62 (33–87)	10.6 months,	Ipilimumab	L	L	L	L	L	L	L
Ferruci et. al, Oncotarget 2017 **[53]**	Retrospective cohort (Italy)	128	Cutaneous 78%, uveal 8%, mucosal 9%	LDH, relative lymphocyte counts and eosinophil counts	B	Complete blood count	P	D	LDH, visceral mets, lymphocyte count	60.5 (IQR 53–71)	11 months	Anti-CTLA-4 n=117,combined chemo n =11	M	L	L	L	L	M	M
Flem-Karlsen et. al, Plos One 2020 **[54]**	Cohort (Norway)	53	Cutaneous 53%, acral/ desmoplastic 5%, unknown 42%.	Soluble receptor tyrosine kinase AXL	B	Immunohisto-chemistry staining + ELISA	P + D	D	N/A	65 (25–94)	NR	Ipilimumab	H	L	L	L	H	H	H
Forschner et. al, J. Immunother. Cancer 2019 **[55]**	Prospective cohort (Germany)	35	57% cutaneous, 17% occult, 11% uveal, 9% acral, 6% mucosal	tumor-associated genes (TMB and circulating DNA)	T+B	DNA isolation from both blood and tissue	P	D	N/A	55(IQR 47-70)	213 days (IQR 175–272).	Ipilimumab + nivolumab	H	M	L	L	H	H	H
Frankel et. al, Neoplasia 2017 **[56]**	Prospective cohort (US)	39	NR	Human gut microbiota and metabolites	F	Metagenomic Shotgun Sequencing (MSS) on Fecal gDNA	P	D	N/A	68 (37-92	NR	Ipi, nivo, ipi plus nivo, or pembro	H	H	L	L	L	H	H
Fröhlich et. al, eBioMedicine 2020 **[57]**	Retrospective cohort + case study (Germany, US)	121	Cutaneous 73%, acral 7%, mucosal 7%, unknown 13%	TNFRSF9 DNA methylation and mRNA levels (available in 121 pts)	T	mRNA expression analysis + methylation analysis	P	D	N/A	NR	NR	Anti-PD-1 Ab	M	M	M	L	H	H	L
Fujimura et. al, Frontiers Oncol. 2018 **[58]**	Cohort (Japan)	75	59 cutaneous melanoma and 16 non-cutaneous (NC) melanoma	Serum levels of sCD163	B	ELISA	P + D	D	N/A	Cutaneous 68 (31–9), NC 66 (54–82)	At least 3 months	Nivolumab	M	L	M	M	H	H	M
Fujimura et. al, Frontiers Med. 2019 **[59]**	Prospective cohort (Japan)	46	Mucosal (30%)	Serum levels of CXCL5, CXCL10, and CCL22	B	ELISA	P	D	N/A	67 (33–93)	NR	Nivolumab	H	H	H	M	L	H	H
Galore-Haskel et. al, Oncotarget 2015 **[60]**	NR, cohort from Israel	13	NR	miR222 expression + ADAR1	B	RNA isolation + quantitative PCR	P	D + V	N/A	NR	NR	Ipilimumab	H	H	H	L	H	H	H
Gambichler et. al, J. Euro. Acad. Dermat. Venereol. 2018 **[61]**	Retrospective selected from databases (Germany)	52	Cutaneous 58%, unknown primary 13,5%, other 29%	WBC, AMC, AEC, ALC, LDH, S100, thrombocytes, Vit D + lymphocyte subtypes	B	Flow cytometry + ELISA	P + D	D	LDH, BRAF status	70 (38-88)	NR	Ipilimumab	M	M	M	L	L	M	H
Gao et. al, Cell, 2016 **[62]**	Retrospective cohort (US)	16	NR	Genomic changes of IFNg pathway genes	T	Whole exome sequencing	D	D + V	N/A	NR	NR	Ipilimumab	M	M	L	M	L	H	H
Gebhardt et. al, Cin Cancer Res 2015 **[63]**	Retrospective cohort (Germany)	59	Cutaneous 68%, Mucosal 2%, Uveal10%, Occult 12%, Unclassified 8%	Myeloid cells + related inflammatory mediators. WBC, AEC, ANC, AMC. MDSC subsets.	B	Laboratory analysis and multicolor flow cytometry + ELISA	P + D	D	N/A	65.2 (32–84)	At least 12 weeks	Ipilimumab	H	M	H	L	L	H	H

Paper	Study design	N Melanoma patients	Type of melanoma	Biomarker of interest	Tumor (T) or blood (B)	Type of test	Timing of biomarker	Study phase	Adjustment for relevant confounders	Median age (range)	Median follow-up	Type of treatment	Quality assessment						
													Risk of bias	Participation	Attrition	Prognostic	Outcome	Adjustment	Statistics
				S100A8/A9, HMGB1, CCL11.															
Ghorani et. al, Annals Oncol. 2018 **[64]**	Retrospective selected from 5 cohorts	131	NR	Binding affinity of mutated peptides for MHC class I	T	Neopeptide prediction and differential agretopicity index	P	D	N/A	Different in all cohorts	Different in all cohorts	Different in all cohorts	M	L	M	L	L	H	M
Gide et. al, Cancer Cell, 2019 **[65]**	Retrospective from clinical trial (Australia)	120	NR	Immune profiles of infiltrating T-cells	T	Opal Multiplex Immunofluorescent Staining using CYToff + flow cytometry, subsequent genome profiling	P + D	D	N/A	62 years	Median OS responder: not reached, non - responder 5.5 months.	Anti-PD-1 (n = 63) or combined anti-PD-1 and anti-CTLA-4 (n = 57)	M	L	M	L	L	H	H
Goltz et. al, JCI insight 2018 **[66]**	Retrospective co (Germany)	520	NR	CTLA-4 promotor methylation (mCTLA4)	T	Infinium Human Methylation 450 BeadChip, methylation-specific quantitative real-time PCR in DNA FFPE	P	D	N/A	Mean 69 (45-90)	NR	No immunotherapy (n=470) and ipilimumab with nivolumab (n=50)	M	M	M	L	L	M	M
Gopalakrishnan et al, Science, 2017 **[67]**	Prospective cohort (USA)	89	NR	Gut microbiome	F	Taxonomic profiling via 16S rRNA gene sequencing.	P	D	LDH, stage, prior treatment, sex	mean 65 oral group, 66 for fecal	At least 6 months of follow up	Anti-PD-1 Ab	M	L	H	L	L	M	L
Graves et. al, Frontiers Med, 2019 **[68]**	Prospective cohort (Australia)	16	NR	Exhaustion markers on T-cells including PD-1, CTLA-4, LAG-3, TIM-3	B	Flow cytometry	P + D	D	N/A	Median NR (48-80)	NR	Pembrolizumab	H	H	H	L	H	M	M
Hamid et. al, J. Transl. Med. 2011 **[69]**	Prospective RCT (Europe, US, South America)	82	Ocular melanoma excluded	TIL, FoxP3, granzyme B, perforin, CD4, CD8, CD45RO, IDO. Tumour mRNA, 20 SNPs, deletions in 10 immune-related genes	B	Immunohisto-chemistry + microassay. tumor RNA extraction and analysis, SNP	P + D	D	N/A	55.0 (23.0-87.0)	8.9 months and 8.6 months, respectively.	Ipilimumab (3 mg/kg (n = 40) + 10 mg/kg (n = 42)	M	M	L	L	M	H	H
Hamid et. al, Ann Oncol 2019 **[70]**	Prospective phase I clinical trial	655	Uveal n=8, other types non-uveal	18-gene T-cell-inflamed GEP: CCL5, CD27 -274 , -276 -8A, CMKLR1, CXCL9, CXCR6, HLA, IDO1, LAG3, NKG7, PDCD1LG2 PSMB10, STAT1, TIGIT.	T	Gene expression profiling	P	D	N/A	Mentioned in Ribas, JAMA 2016	Idem	Pembrolizumab	M	M	H	L	L	H	M
Heidelberger et. al, Invest new drugs 2017 **[71]**	Retrospective cohort (France)	63	Superficial Spreading 52%. Nodular 17,5%,	LDH, CRP leucocyte/lymphocyte ratio, BRAF status	B	NR	P	D	Brain metastases, PS, liver metastases,	65 (22–90)	7 months (1-18)	Anti-PD-1 Ab	L	L	L	L	L	L	L

Paper	Study design	*N* Melanoma patients	Type of melanoma	Biomarker of interest	Tumor (T) or blood (B)	Type of test	Timing of biomarker	Study phase	Adjustment for relevant confounders	Median age (range)	Median follow-up	Type of treatment	Quality assessment						
													Risk of bias	Participation	Attrition	Prognostic	Outcome	Adjustment	Statistics
			Mucosal 6,5%, Acral 8% Other 16%						Neutrophil/lymphocytes, treatment, stage, age.										
Hogan et. al, Cancer Immun. Res. 2019 **[72]**	Retrospective cohort + manually selected (Netherlands, Switzerland)	80	NR	TCR Repertoire Profiling (o.a. DE50)	B	N-plex PCR on genomic DNA	P	D	LDH, ALC, sex, eosinophils, S100, NRAS, BRAF, basophils, age, leucocytes	5 pts <40, 25 pts 40-55, 32 pts 56-70, 18 pts >70	11.38 months (median 13.08) for died pts and 32.75 (median=36.49) for alive pts	Anti-CTLA4 (n=42) or anti-PD1 (n=38) Ab	M	M	M	L	L	L	L
Hong et. al, Proc Natl Acad Sci USA 2018 **[73]**	Prospective cohort (DC 33 pts, VC 49 pts) (US)	84	Acral-lentiginous or other	Melanoma CTCs	B	Quantitative 19-gene digital RNA signature (CTC score)	P + D	D	N/A	Mean 63	24 months (11–26 mo)	Ipilimumab / pembrolizumab	M	L	L	L	L	M	M
Huang et. al, Nature, 2017 **[74]**	Cohort + RCT (US)	29 + 18	NR	Immune profiling of exhausted-phenotype CD8 T cells	T	Cytometry, T-cell receptor sequencing, immunohistochemistry and CytoF.	P + D	D	TMB,	57 (29-82)	NR	Pembrolizumab (second line)	M	L	L	M	M	M	H
Hugo et. al, Cell, 2016 **[75]**	Cohort	38	NR	TMB, neo epitopes, nucleotide variants, gene expression	T	WES, RNA seq	P	D	N/A	NR	N/A	Anti-PD-1 Ab	H	H	H	L	L	H	H
Iivanainen et. al, ESMO open, 2019 **[76]**	Retrospective cohort (UK)	69	NR	CRP, NLR, LDH, total leucocytes, total lymphocytes	B	Peripheral blood count	P	D + V	CRP, NLR, number of lymphocytes, LDH	66	NR	anti-PD-(L)1 Ab	M	L	L	L	L	H	H
Indini et. al, Tumori J. 2019 **[77]**	Retrospective cohort (Italy)	173	Cutaneous 75%, mucosal 7%, uveal 8%, unknown 10%	LDH, complete blood count	B	Peripheral blood count	P	D	N/A	62 (18–85)	9 months (1–57)	Pembrolizumab / nivolumab	M	L	L	L	L	H	M
Inoune et al, Oncoimmulol, 2016 **[78]**	Prospective cohort (Japan)	13	Uveal 1, others not clearly described	PD-L1 PD-L2, Granzyme A, HLA-A, CD4, FOXP3, IL-10, TBX21 and GATA3	T	gene expression assays + ctDNA synthesis with TCR amplifying	P + D	D	N/A	NR	NR	Nivolumab	H	M	M	L	H	H	H
Jacquelot et. al, Nat Commun, 2017 **[79]**	Retrospective + prospective from 8 cohorts + RCTs	190	NR	Blood immune parameters + functional immune reactivity of TILs	B	Peripheral blood count + flow cytometry + ELISA	P	D	N/A	NR	30 months (95% CI: 26–34)	Ipi,, ipilimumab + nivolumab or nivolumab	H	H	H	L	M	M	H
Jensen et. al, J. Immunother Cancer, 2018 **[80]**	Prospective cohort (Denmark)	66	NR	Collagen and vimentin turnover	B	C1M, C3M and C4M, and citrullinated and MMP-degraded vimentin with ELISA	P + D	D	Age, LDH, prior systemic treatment	67 (35–83)	473 days (40–1258)	Ipilimumab	L	M	L	L	L	L	L
Ji et. al, Cancer Immun. Immunother 2012 **[81]**	Prospective phase II RCT (US)	45	NR	GEP	T	Gene expression profiling and qPCR analysis	P + D	D	N/A	NR	NR	Ipilimumab	H	M	H	L	H	H	H

Paper	Study design	N Melanoma patients	Type of melanoma	Biomarker of interest	Tumor (T) or blood (B)	Type of test	Timing of biomarker	Study phase	Adjustment for relevant confounders	Median age (range)	Median follow-up	Type of treatment	Quality assessment						
													Risk of bias	Participation	Attrition	Prognostic	Outcome	Adjustment	Statistics
Jiang et. al, Nature Medicine 2018 **[82]**	Retrospective selected from Van Allen/Hugo	67	NR	Genome-wide expression signatures for T cell dysfunction exclusion	T	TIDE prediction score: computional model of genome -wide expression signatures of T-cells	P	D + V	Age, gender stage, treatment line	NR	NR	Anti-CTLA-4 Ab (42), antiPD-1 Ab (25)	H	H	H	L	L	H	H
Johnson et. al, Clin Cancer Res. 2018 **[83]**	Retrospective selected (US)	166	NR	PD1/PD-L1 interactions and HLA-DR/IDO-1 co-expression	T	Digital pathology: fluorescence staining of FFPE	P	D + V	Metastatic stage, LDH, age, previous therapies	DC mean 57,3 (SD 13,9); VC mean 63,4 (SD 14,3)	NR	Anti-PD-1 Ab	M	H	H	M	L	M	M
Johnson et. al, Nature Commun 2015 **[84]**	Development retrospective selected, validation prospective (US)	53	NR	MHC class I and II expression, PD-L1, CD4+ and CD8+ T-cells.	T	IHC	P	D + V	N/A	DC: 56 (27-81)	NR	DC: anti PD-1 Ab (n=28) or antiPD-L1 (n=2). VC: anti-PD-1 Ab	H	H	H	L	L	H	H
Johnson et. al, Cancer Immunol Res 2016 **[85]**	Retrospective selected (US)	55	Cutaneous: 44, non cutaneous: 14, unknown: 7	TMB + TCR	T	NGS and T-cell receptor sequencing	P	D + V	Stage, age, gender, and prior ipilimumab.	DC 55 (33-80); VC 62 (32-85)	NR	DC: anti PD-1 Ab (n=29); antiPD-L1 (n=3). VC anti PD-1 Ab	M	H	M	M	L	M	M
Kalaora et. al, Nature commun 2020 **[86]**	Retrospective analysis of 2 cohorts (US)	47	NR	PSMB8 and PSMB9, two components of the immunoproteasome	T	PSMB8 and PSMB9 gene expression in tumour	P	D	N/A	NR	NR	Anti CTLA4 (ipi) / anti PD1 (pembro/nivo) Ab	H	H	H	L	H	H	H
Karachaliou et. al, Therap.Adv. Medic. Oncology 2018 **[87]**	Retrospective manually selected form 3 cohorts (Spain)	21	NR	IFNG gene expression	T	RNA of STAT3, Rantes, YAP1, CXCL5, DNMT1, RIG1, ET1, EOMES, IFNG PD-L1 CTLA4, IKBKE NFATC1	P	D	N/A	54(49-61)	12,4 months	Pembrolizumab	M	H	H	L	M	M	L
Kasanen et. al, Cancer Immun, Immunother 2020 **[88]**	Prospective manually selected (Finland)	17	NR	T-, NK and NKT cells in blood (markers CD3, CD4, CD8, CD25, CD56, GranzymeB, LAG3 and PD1); cytokines	T	Blood counts, PB immunophenotype, serum cytokine profiles, TILs in TMA blocks with immunohistochemistry	P	D	N/A	68 (53-84)	21 months	Anti-PD-1 Ab (nivo/pembro)	H	H	H	L	H	H	H
Kelderman et. al, Cancer Immun, Immunother 2014 **[89]**	Prospective Expanded excess programme (NL, UK)	230	NR	ALC, S100, ESR, LDH,	B	At baseline, every 3 weeks during treatment and 3 months during follow-up	P + D	D + V	M-stage, WHO performance status	NL55 (22–88) UK 58 (18–84)	NL17,9 UK19 months	Ipilimumab	M	L	M	L	L	L	L

Paper	Study design	N Melanoma patients	Type of melanoma	Biomarker of interest	Tumor (T) or blood (B)	Type of test	Timing of biomarker	Study phase	Adjustment for relevant confounders	Median age (range)	Median follow-up	Type of treatment	Quality assessment						
													Risk of bias	Participation	Attrition	Prognostic	Outcome	Adjustment	Statistics
Keller et. al, Acta DermatologVenereology 2019 **[90]**	Retrospective manually selected (France)	22	NR	ctDNA	B	Digital droplet PCR was used to quantify BRAF and NRAS mutations	P	D	N/A	66 (43-89)	135 days (20-620)	Pembro, ipilimumab, nivo or a combination	H	H	H	L	M	H	H
Khattak et. al, Oncologist 2020 **[91]**	Prospective manually selected (Australia)	40	NR	PD-L1 expression on CTC's	B	Multiparametric flow cytometry (22C3 pharmDx)	P + D	D	PS, age, sex, stage, liver metastasis, line of treatment	Mean 71	25.5 months, (9.4 – 42.4)	Pembrolizumab	M	H	H	L	M	L	L
Khoja et. al, Cancer Med 2016 **[92]**	Retrospective cohort (Canada)	183	NR	NLR, platelet/ lymphocyte + eosinophil/lymphocyte ratios, LDH	B	Blood count and LDH assay	P + D	D	Gender, age, M stage, performance status,	58	7.5 months (0.3–49.5)	Ipilimumab	M	M	M	L	L	L	L
Kirchberger et. al, European J Cancer 2018 **[93]**	Retrospective cohort (Germany, Switserland)	364	NR	NRAS	T	NRAS mutation analysis	P	D	N/A	64 (20-87)	NR	Anti-CTLA-4. anti-PD-1, combination	M	L	M	L	L	H	M
Kondo et. al, Intern J Clin. Oncology 2019 **[94]**	Retrospective cohort (Japan)	39	Mucosal 22, cutaneous 17	CRP to albumin ratio, ANC,ALC, LDH, CRP, NLR< PLR, platelets , albumin	B	Chemistry	P	D	Age, gender, metastatic sites, dose, BRAF status	65 (28-84)	11,9 months (5-36.1)	Nivolumab	M	H	H	L	M	M	L
Krieg et. al, Nature Med 2018 **[95]**	Retrospective cohort (Switserland)	20	NR	Immune celll subsets	T+B	HD single cell mass cytometry	P + D	D	N/A	NR	NR	Anti-PD1	H	H	H	L	H	H	H
Kubo et. al, J Dermatology Scn 2019 **[96]**	Retrospective cohort (Japan)	29	non-CSD 28, CSD 13, acral 43, mucosal 19	Hepatocyte growth factor (HGF)	T	Elisa	P	D	LDH, PS, and metastatic stage	64 (28-83)	NR	Anti-PD-1 Ab (nivo, pembro)	M	H	H	L	L	M	M
Kumpers et. al, Frontiers Med 2019 **[97]**	Retrospective cohort (Germany)	32	NR	TILs	T	Immune cell infiltration IHC staining	P	D	N/A	64	NR	Anti-CTLA-4, anti-PD-1 Ab or both	H	H	H	L	L	M	H
Lee et. al, BMC Cancer 2019 **[98]**	Retrospective cohort (Asia)	152	acral 38%, mucosal 31%, cutaneous 24%, uveal 2%, unknown 5%.	NLR and NGS, BRAF, KIT	T+B	Blood cell count and NGS	P	D	Age, ECOG PS, histologic subtype, M stage, mutations, LDH	61 (21-82)	18,8 months (3-42,3)	Anti-PD-1 Ab (nivo/pembro)	M	L	M	L	L	M	L
Lee et. al, Ann Oncol 2017 **[99]**	2 prospective cohorts (Australia)	all.	BRAF , KIT, or NRAS mutated	ctDNA	B	ctDNA analysis	P + D	D	LDH, PS, tumour stage and disease volume	45% >65, median NR	17,5 months (10,5 -28)	IPI or PEMBRO /NIVO or both	M	H	M	H	L	M	M
Li et. al, Nature commun 2019 **[100]**	Retrospective selected from trials (US + others)	78	NR	Kynurenine/tryptophan ratio and 201 other metabolites	B	Liquid chromatography mass spectrometry	P + D	D	Sex, age, race, region and BRAF	NR	NR	Anti-PD-1 Ab (nivolumab)	H	H	H	L	H	H	M
Liu et. al, Clin Cancer Res 2019 **[101]**	Retrospective selected from Van Allen/Snyder	174	Cutaneous	TMB and copy number alteration	T	Whole genome sequencing	P	D	Gender, age	NR	NR	Ipilimumab	M	H	H	L	L	M	M
Luksza et al. Nature, 2017 **[102]**	Retrospective cohort	167	NR	Fitness model of tumour-immune interactions: neoantigen fitness +	T	Whole genome sequencing	P	D	N/A	NR	NR	Ipilimumab	H	H	H	L	M	H	H

Paper	Study design	N Melanoma patients	Type of melanoma	Biomarker of interest	Tumor (T) or blood (B)	Type of test	Timing of biomarker	Study phase	Adjustment for relevant confounders	Median age (range)	Median follow-up	Type of treatment	Quality assessment						
													Risk of bias	Participation	Attrition	Prognostic	Outcome	Adjustment	Statistics
				presentation by the MHC + recognition by T cells															
Maccalli et. al, OncoImmunology 2017 **[103]**	Retrospective cohort (Italy)	162	Cutaneous 85%, mucoasl 7%, ocular 6%, acral 2%	Soluble activating receptor NK cell group 2 member ligands (MICA, MICB, ULBP 1,2 3)	B	Elisa	P + D	D	Gender, age, performance status, LDH, stage	62	NR	Anti-CTLA-4 Ab or anti-PD-1 Ab or both (pembro, nivo, ipi)	M	H	H	L	M	L	M
Madonna et al, OncoImmunology **[104]**	Retrospective cohort from Expanded Database (Italy)	114	Advanced stage IV melanoma	PD-L1 expression, BRAF, NRAS,	T	PD-L1 expression on FFPE tumour biopsies	P	D	N/A	61 (range 25–90)	NR	Ipilimumab	H	H	H	M	L	H	H
Martens et. al, Clin Cancer Res 2016 **[105]**	Retrospective cohort (US, Europe)	615	Cutaneous (100%)	Peripheral blood biomarkers	B	Celcounts, chemistry, PBMC flow-cytometry	P	D + V	N/A	58	19 months for pts alive, and 5 months for pts who died	Ipilimumab	M	L	M	L	L	M	M
Martens et. al, Clin Cancer Res 2016 **[106]**	Retrospective cohort (US, Europe)	82	Cutaneous (100%)	Circulating immune cell populations	B	Flow cytometry	P + D	D	N/A	60	NR	Ipilimumab	M	L	M	L	M	H	M
Mastracci et. al, Cancer Immun Immunother 2020 **[107]**	Retrospective cohort (Italy)	17	Cutaneous 88%, Mucosal 6%, Unknown 6%	TILs	T	Histology, IHC	P + D	D	Age, sex, brain mets, pre-IPI therapies, tissue localization + time of IPI therapy	62 (33-88)	NR	Ipilimumab	M	H	H	M	L	M	L
Matson et. al, Cancer Immunother 2018 **[108]**	Prospective cohort	42	NR	Stool microbiome	F	DNA sequence–based bacterial identification: integration of ribosomal RNA gene + metagenomic shotgun sequencing, + quantitative PCR	P	D	N/A	NR	NR	Anti-CTLA-4 Ab or anti-PD-1 Ab	H	H	H	L	H	H	L
Mc Granahan, Science 2016 **[109]**	Retrospective cohort	64	NR	Neoantigen intratumor heterogeneity and neoantigen burden	T	neoantigen and clonality analysis (Bioinformatics pipeline)	P	D	N/A	57-66 (18-90)	NR	Anti-CTLA-4 Ab	H	H	H	H	H	H	H
Meyer et. al, Cancer Imm. Immunother 2014 **[110]**	Retrospective cohort (Switzerland)	15	NR	MDSCs	B	Flow-cytometry PBMC's	P + D	D	N/A	65 (37-86)	Range 3–30 months	Ipilimumab	H	H	H	L	M	H	M
Minowa et. al, J Dermat. 2018 **[111]**	Prospective	21	NR	NLR	B	Blood cell count	P	D	N/A	74 (34–91)	NR	Anti-PD-1	H	H	H	L	L	H	H
Moreira et. al, Immunotherapy 2017 **[112]**	Retrospective cohort	86	NR	Eosinophils	B	Blood cell count	P + D	D	N/A	60	NR	Anti-CTLA-4, anti-PD-1 or both (pembro, nivo, ipi)	H	H	H	L	M	H	H

Paper	Study design	N Melanoma patients	Type of melanoma	Biomarker of interest	Tumor (T) or blood (B)	Type of test	Timing of biomarker	Study phase	Adjustment for relevant confounders	Median age (range)	Median follow-up	Type of treatment	Quality assessment						
													Risk of bias	Participation	Attrition	Prognostic	Outcome	Adjustment	Statistics
Morello et. al, J Transl Med 2017 **[113]**	Retrospective cohort (Italy)	37	NR	Soluble CD73 enzyme activity	B	AMP hydrolysing assay (enzyme activity)	P	D	Age, BRAF status, LDH, brain mets, gender, line of treatment	62	24 months	Nivolumab	M	H	H	M	L	L	L
Morrison et. al, J. ImmunotherCancer 2018 **[114]**	Retrospective cohort (US)	77	NR	PD-L1 expression, CD8+ T-cel infiltration, T<B, tumor transcriptomics	T	IHC, whole exon sequencing.	P	D + V	N/A	Mean 61	16,2 months	Anti-CTLA-4, anti-PD-1 or both (pembro, nivo, ipi)	H	H	H	L	H	H	H
Muto et. al, J. Dermatology 2019 **[115]**	Retrospective cohort + clinical trial (Japan)	30	Cutaneous 14% Mucosal 7%, uveal 3%, conjuctival 1%	LDH, CRP, neutrophils, NLR	B	Blood cell count, chemistry	P	D	Performance status	60,5 (17-18)	NR	Ipilimumab after anti-PD-1	H	H	H	L	L	H	H
Nakamura et. al, Oncotarget 2016 **[116]**	Retrospective cohort (Japan)	98	Acral 17.3%, CSD 7.1%, Non-CSD 20.4%, Mucosal 36.7%	LDH, CRP, WBC, monocytes, eosinophils, lymphocytes	B	Cell counts, chemistry	P + D	D	Diameter tumor, PS	66,5 (17-93)	NR	Nivolumab	H	L	H	L	L	H	H
Nakamura et. al, Jap J Clin Oncol 2019 **[117]**	Retrospective cohort (Japan)	45	Mucosal 17, acral 12, superficial spreading 10, lentigo maligna 3, nodular 1	NLR, WBC, monocytes, eosinophils, lymphocytes, LDH	B	Blood cell counts	P	D	Stage, melanoma type	Mean 69 (42-85)	NR	Anti-PD-1 Ab (nivo, pembro)	H	H	H	L	L	M	H
Nie et. al, Aging, 2019 **[118]**	Retrospective selected form TCGA	228	Stage I-IV	Immunoscore = (1.13 × fraction naive B cells) + (1.36 × mem B cells) + (5.92 × eosinophils) + (9.70 × follicular T cells) + (15.34 × of Tregs) - (1.14 × macrophages) - (2.31 × plasma cells) - (4.52 × δT cell)	T	RNA gene expression profiles of tumour infiltrating immune cells	P	D	N/A	50 (38-85)	NR	Anti-PD-1 Ab	H	H	H	L	M	H	L
Nonomura et. al, OncoImmunology 2016 **[119]**	Prospective cohort (Japan)	46	NR	CD4C Tcell (Treg, Th1, Th2, Th9, Th17, Th22), CD8C T cells, cytokines (IFNg,IL-4, IL-9, IL-10, TGF-b)	B	Flow cytometry PBMC, Elisa cytokines	P + D	D	N/A	Mean 66 (34-89)	NR	Nivolumab	H	H	H	L	L	H	L
Nosrati et. al, British J Cancer 2017 **[120]**	Retrospective selected from KEYNOTE (Switserland, US)	315	Uveal melanoma excluded	LDH, leukocytes	B	Chemistry	P	D + V	Age, sex, previous ipi treatment, liver mets	Mean 62.5, SD 14.3	NR	Anti-PD-1 Ab (nivo, pembro)	M	L	L	L	L	L	M
Okuhira et. al, BioScience Trends 2018 **[121]**	Retrospective cohort (Japan)	16	Mucosal 44%, Acral 25%, Non-CSD: 19%, CSD: 6%	WBC, ALC, ANC, AMC, AEC, ABC, LDH, CRP, ESR, and 5-S-CD	B	Blood cell counts, chemistry	P + D	D	N/A	71 (48-87)	NR	Nivolumab	H	H	H	L	M	H	H
Pan et. al, Permanente J 2018 **[122]**	Retrospective cohort (US)	108	NR	Neutrophil count, platelet count, lymphocytes	B	Blood cell count	P	D	Age, Charlson comorbidity index, PS, BRAF status	65 (23-91)	118 days	Anti-PD-1(nivo, pembro)	L	L	L	L	L	L	L

Paper	Study design	N Melanoma patients	Type of melanoma	Biomarker of interest	Tumor (T) or blood (B)	Type of test	Timing of biomarker	Study phase	Adjustment for relevant confounders	Median age (range)	Median follow-up	Type of treatment	Quality assessment						
													**Risk of bias**	Participation	Attrition	Prognostic	Outcome	Adjustment	Statistics
Perrone et. al, J Immunother 2020 **[123]**	Retrospective cohort (Italy)	28	NR	Plasma Cholesterol	B	Routine chemistry	P	D	PS, pleural effusion, previous response, statin	70 (32-89)	21,3 months	Anti-PD-1 86%, anti-PD-L1, anti-CTLA-4, combination.	H	H	H	L	L	H	M
Pirozyan et. al, Frontiers Immun 2020 **[124]**	Retrospective cohort (Australia)	42	NR	PBMC's and their effector molecules	B	Mass cytometry immunophenotyping	P	D	N/A	69-77, (41-91)	NR	Anti-PD-1 Ab	H	H	H	H	L	H	H
Pistillo et. al, Cancer Immun Immunother 2019 **[125]**	Retrospective cohort (Italy)	113	Cutaneous (N = 90, 79.7%), mucosal (N = 12, 10.6%), unknown 11, 9.7%)	Soluble CTLA-4	B	ELISA	P + D	D	Age, gender, time from diagnosis, LDH, brain liver/cutaneous metastases	58 (28-85)	7,3 months (0,1-52,9)	Ipilimumab	M	L	L	L	M	M	L
Postow et. al, J Immunother Cancer 2015 **[126]**	Retrospective cohort (US)	12	NR	TCR repertoire richness (observed V-J rearrangements) evenness (similarity between frequencies of V-J rearrangements)	B	PCR	NR	D	N/A	Clinical benefit: 57 (38-78), no clinical benefit: 67 (52-77)	At least 9 months	Ipilimumab	H	H	H	L	H	H	M
Retseck et. al, J Transl Med 2018 **[127]**	Retrospective cohort (US, other countries)	31	Stages IIIb and IIIc. Cutaneous 81%, mucosal 16% or unknown primary 2%	Treg, MDSC, CD4+ CD8+ T cells specific to tumor-associated antigens (Gp-100, MART-1, NY-ESO-1)	B	Flow cytometry	D	D	N/A	54 (40-87)	NR	Anti-CTLA-4 Ab	H	M	H	L	M	H	H
Riaz et. al, Nature Gen 2016 [128]	Cohort from US and Germany	174	NR	19 recurrently mutated genes in melanoma, discovered in TCGA	T	Whole exome sequencing	P	D	Stage	NR	NR	Anti-CTLA-4	M	H	H	L	L	M	M
Riaz et al, Cell (2016) **[129]**	Data from prospective cohort	68	NR	comprehensive genomic analyses (TMB, NAL, TCR richness and evenness)	T	Whole-exome, transcriptome, and TCR sequencing	P + D	D	N/A	NR	NR	Nivolumab (after IPI or IPI-naive)	H	H	H	L	L	H	H
Ribas et al, Cancer Immunol Res (2016) **[130]**	Data selected from clinical trial	53	NR	TILS and TIL subsets	T	Multicolor flow cytometry using computational approaches	P + D	D	N/A	Mean 68 (R) and 54 (NR)	At least 3 months	Pembrolizumab	H	H	M	L	M	H	H
Rodig et. al, Cancer 2018 **[131]**	Retrospective selected from CHECKMATE 064/069	227	NR	MHC Class I and II expression on tumour cells	T	Immunohisto-chemistry	P	D + V	N/A	NR	NR	Ipi or nivo or combination (only in validation)	M	M	H	L	L	H	L
Roh et al, 2018, Sci Transl Med **[132]**	Prospective cohort Expanded Acces Program + VC from Van Allen	56	Acral 7, cutaneous 39, mucosal 1, unknown 9	TCR, TMB copy number alteration, neoantigen load	T	Whole exome sequencing + formalin-fixed, paraffin-embedded blocks	P	D + V	N/A	57 (27-86)	NR	Anti-CTLA-4 Ab (56), after progression anti-PD-1 Ab (48)	M	L	M	L	L	H	H
Rosner et. al, Cancer Med 2018 **[133]**	Retrospective selected from RCTs	209	Stage IV and unresectable stage III	Lymphocytes, eosinophils, monocytes,	B	Blood cell count	P	D	LDH, metastatic sites	60,5 (22-86,4)	13,1 months	Ipilimumab +nivolumab	M	L	M	L	L	L	M

Paper	Study design	N Melanoma patients	Type of melanoma	Biomarker of interest	Tumor (T) or blood (B)	Type of test	Timing of biomarker	Study phase	Adjustment for relevant confounders	Median age (range)	Median follow-up	Type of treatment	Quality assessment						
													Risk of bias	Participation	Attrition	Prognostic	Outcome	Adjustment	Statistics
	/commercial ICI use (US)			neutrophils, and basophils															
Sade-Feldman et. al, Clin Cancer Res 2016 **[134]**	Retrospective cohort (Israel)	56	Skin (66%), ocular (25%), mucosal (9%)	CD33+ CD11b+HLA-DR- MDSC	B	Flow cytometry	P	D	LDH	60,7	NR	Ipilimumab	M	M	M	L	L	L	M
Sanmamed et. al, Ann Onco 2017 **[135]**	2 retrospective cohorts (US)	44	NR	Serum IL-8	B	ELISA	P + D	D + V	N/A	DC 57.7 (33-79) VC 55 (40-76)	15 months	Pembro, nivo or combination ipi+nivo.	M	L	M	L	L	H	L
Seremet et. al, J. Transl.Med.2019 **[136]**	Prospective selected from RCT (Belgium)	85	NR	ctDNA (BRAF and NRAS mutated), LDH, CRP	B	PCR	P + D	D	ctDNA, LDH, CRP, tumor sites PS	57 (27-82)	84 weeks	Pembrolizumab	M	M	M	L	L	M	L
Shukla et. al, Cell, 2018 **[137]**	DC retrospective (Van Allen), VC prospective (CHEKMATE 064) (US, Germany)	146	Weber cohort: cutaneous (85%), ocular (3%), unknown (4%) , Allen/hugo not described	MageA cancer germline antigens	T	RNA sequencing	P + D	D + V	Age, gender, number of pre-therapies, M-stage, LDH and neoantigen load	NR	NR	Anti-CTLA4 Ab (81); AntiPD-1 Ab (63)	M	H	H	L	M	L	M
Simeone et. al, Cancer Imm Immun 2014 **[138]**	Retrospective (Italy)	95	Cutaneous 80%, ocular 7% or mucosal 7%	LDH, CRP, FoxP3/regulatory t cells, ALC WBC	B	Flow cytometry	P	D	N/A	58 (17-84)	24 months (1-36)	Ipilimumab	M	L	L	L	L	H	H
Simon et. al, Cancer Res 2017 **[139]**	Prospective cohort (France)	9	NR	Melan-A specific T-cell repertoire	B	PBMCs stimulation with Melan-AA27L peptide.	P + D	D	N/A	(54- 89)	At least 2 months	Anti-PD-1 (nivolumab)	H	H	H	L	L	H	H
Simon et. al, OncoImmunology 2020 **[140]**	Cohort (Germany)	32	NR	Circulating eosinophils and related serum inflammatory factors	B	Blood counts, flow cytometry (basophils measured as % within live granulocytes)	P + D	D	N/A	69,5 (15-83)	At least 32 days	Pembrolizumab or nivolumab +ipilimumab	H	H	H	L	L	H	H
Smithy et. al, J. ImmunoTh Cancer 2017 **[141]**	Retrospective cohort (US)	47	Non-ocular melanoma	PD-L1 transcription factor, interferon regulatory factor 1	T	Immunofluoresce nce	P + D	D	Age, race, sex, stage, mutations, prior ICI	62	NR	Nivolumab, pembro or ipi+nivo	M	H	H	L	L	L	M
Snyder et. al, NEJM 2014 **[142]**	Cohort from US	64	Cutaneous (44), uveal (1) acral (5), unknown (7)	Somatic mutations, TMB, candidate neoantigens	B	Whole exome sequencing	P + D	D + V	N/A	57-66 (18-90)	NR	Ipilimumab or tremelimumab (n=3)	H	H	H	L	L	H	M
Subrahmanyam et. al, J. Immunoth Cancer 2018 **[143]**	Retrospective cohort (US)	67	NR	40 surface and intracellular markers on peripheral blood mononuclear cell (PBMC)	B	Mass cytometry (CyTOF) + immune profiling	P	D	N/A	Ipi responders mean 62.3; NR 56.4; anti-pd1 responders 61.3 NR 61.4	At least 180 days	Anti-CTLA-4 (24) or anti-PD-1 (n=40) Ab	M	M	M	L	L	H	H
Tarhini et al, Plos One, 2014 **[144]**	Cohort from USA	35	Unknown 1, mucosal 5, cutaneous 29	(monocytic) MDSC, CD20+, Tregs	T+B	Flow cytometry and	P+D	D	N/A	NR	16.1 months for patients still alive	Ipilimumab	M	M	M	L	M	H	H

Paper	Study design	N Melanoma patients	Type of melanoma	Biomarker of interest	Tumor (T) or blood (B)	Type of test	Timing of biomarker	Study phase	Adjustment for relevant confounders	Median age (range)	Median follow-up	Type of treatment	Quality assessment						
													Risk of bias	Participation	Attrition	Prognostic	Outcome	Adjustment	Statistics
						immunohisto-chemistry													
Tarhini et al, J Immunother Cancer, 2015 **[145]**	Cohort from USA	35	Unknown 1, mucosal 5, cutaneous 29	IFN-γ, IL-12 (p40/p70, IL-15, IL-17, IL-2, IL-7, IP-10, IL-13, IL-5, IL-4 IL-1α, IL-1β, IL-6, TNF-α, IL-1RA, IL-2R, IL-8, CRP, IL-17, IFN-α, TGF-β1, IL10, PGE2, VEGF, G-CSF, EGF, HGF	B	xMAP serum assay	P+D	D	N/A	NR	16.1 months for patients still alive	Ipilimumab	M	M	M	L	M	H	H
Tarhini et al, **[146]**, OncoImmunol, 2017	Retrospective analysis of cohort, USA	23	Unknown 1, mucosal 3, cutaneous 23	Gene expression profiling of 22 genes	T	mRNA expression profiling using U133A 2.0 Affymetrix gene chips	P + D	D	N/A	53 (40–87)	NR	Ipilimumab	H	M	M	L	H	H	H
Tietze et. al, European J. Cancer 2017 **[147]**	Prospective cohort (Germany)	30	NR	Circulating CD45ROþCD8þ memory T cells	B	Flow cytometry	P	D	N/A	61 (35-75)	NR	Ipilimumab 21, pembro 9	H	H	H	L	M	H	L
Tietze et. al, Exp Dermatol 2016 **[148]**	Prospective cohort (Germany)	32	NR	NK-cells	B	Flow cytometry	P	D	N/A	Not readible (Not accessible)	Not readible (supplements not accessible	Ipilimumab (n=23), pembro (n=9).	H	H	H	L	L	H	M
Topalian et al, NEJM 2012 **[149]**	Prospective phase I trial (US)	18	NR	PD-L1 expression on tumour cells in 18/104 pts available	T	Immunohisto-chemistry	P	D	N/A	NR	NR	Nivolumab in different doses	H	H	H	L	L	H	H
Tucci et. al, Onco immunol. 2018 **[150]**	Prospective cohort (Italy)	59	Cutaneous (60), mucosal (2) and uveal (1)	PD-1, CD28, ICOS immune checkpoints expressed by T-cell DC cell derived Exosomes	B	Flow cytometry	P + D	D	N/A	59	8,1 month	Ipilimumab	M	M	M	L	L	H	L
Tumeh et. al, Nature, 2014 **[151]**	Retrospective selected from phase I trials (US)	61	NR	T-cells, PD-1, PD-L1, CD8+ T cell activation markers.	T	Immunohistoche mistry, Flow cytometry, NGS, PCR	P + D	D + V	N/A	DC 64 (36-90). VC 55 (26-73)	NR	Pembrolizumab	M	H	H	L	L	M	L
Urun et. al, J Onco Pharmac Practice 2019 **[152]**	Retrospective cohort (Turkey)	97	NR	LDH, Anemia, lymphocytes, neutrophils, ckit, BRAF	T	Routine blood cell counts, PA	P	D	Mets, grade 3–4 toxicity, age, metastatic sites + line of treatment	58 (27-88)	NR	Ipilimumab	M	L	L	M	L	M	H
Uryvaev et. al, Med Oncology 2018 **[153]**	Retrospective cohort (Israel)	30	Stage IV 10%, stage III 26%, stage II 43% and stage I 17%	TIL's	T	Immunohisto-chemistry	P	D	N/A	65 (32-93)	NR	Anti-PD-1 Ab	H	H	H	L	M	H	H
Valpione et. al, European J. Cancer 2015 **[154]**	Retrospective cohort (Italy)	215	Cutaneous 83%, uveal 4%, mucosal 4%, unknown 9%	b2 micro	B	Cytofluorometry for the cells.	P	D + V	N/A	NR	11,5 months	Ipilimumab	L	L	L	L	L	H	L

Paper	Study design	N Melanoma patients	Type of melanoma	Biomarker of interest	Tumor (T) or blood (B)	Type of test	Timing of biomarker	Study phase	Adjustment for relevant confounders	Median age (range)	Median follow-up	Type of treatment	Risk of bias	Quality assessment					
														Participation	Attrition	Prognostic	Outcome	Adjustment	Statistics
Van Allen et. al, Science 2015 **[155]**	Cohort, biobank protocol	150	92 cutaneous, 4 mucosal, and 14 ocular	Tumor specific neoantigens, Mutational load, tumor microenvironment, gene specific enrichment	T	Whole exome sequencing (DNA) matching germlines (n=110/150), tumor whole transcriptome sequencing (n=40/62 due to quality)	P	D	Prior RAF inhibitor treatment and M stage (metastasis)	61,5 (18-86)	NR	Ipilimumab	M	M	M	L	L	L	L
Varn et. al, OncoIogy Immunology 2019 **[156]**	Retrospective selected from TCGA	70	Cutaneous	Immune cell derived GEP: naive B cells, mem B cells, CD8 + T cells, CD4 + T cells, NK or myeloid cells	T + B	Tumors (gene-expression data from TCGA) searched for Immune cell GEP	P	D	TMB, SCNA	NR	NR	Anti-PD-1 (n=28), anti-CTLA-4 (n=42) Ab	H	H	H	L	L	H	H
Vilain et. al, Clin Cancer Res 2017 **[157]**	Retrospective selected from phase I/II/III trials (Australia)	23	NR	Tumour immune microenvironment	T	IHC	P + D	D	N/A	54 (36-79)	27,1 months (1,9-55)	Anti-PD-1 Ab	H	H	H	L	L	H	M
Wagner et. al, British J Cancer 2018 **[158]**	2 Retrospective cohorts (Germany)	238	NR	S100B and LDH	B	NR	P	D	Cerebral metastasis, visceral metastasis, LDH	67,1%>60 (anti-PD1) 60,5%>60 (combination therapy)	9,9 months IQR 4,8-15,7 anti-PD, 6,4 IQR 3,2-10,6 combi	Anti-PD-1 (pembro) or anti-PD-1+ anti CTLA4 (nivo+ ipi)	L	L	L	M	L	M	L
Wagner et. al, J. Immunother. Cancer 2019 **[159]**	2 prospective cohorts (Germany)	71	Cutaneous melanoma	S100A8/A9 gene expression	B	Elisa	P	D	LDH and M stage	NR	NR	Pembrolizumab	M	H	H	L	L	L	L
Weber et. al, Cancer Immun Res 2017 **[160]**	Retrospective selected from 5 cohorts (US + Italy)	289	NR	209 protein signature	B	Mass Spectometry	P	D + V	N/A	56-69 (16-90)	NR	DC anti-PD-1, VC anti-PD-1, Anti-CTLA4 or both	L	M	L	L	L	H	L
Weber et. al, Cancer ImmunRes.2016 **[161]**	Prospective phase I/II study (US)	289	Cutaneous 85%, ocular 3%, unknown 4%	Monocytic MDSCs (CD11b+,CD14+, HLA-DR low)	B	Flow cytometry	P	D	N/A	60	16 months	Nivo 3 mg/kg 24 weeks, then 12 weeks	M	L	L	M	L	H	M
Weide et. al, Clin Cancer Res 2016 **[162]**	Retrospective cohort from KEYNOTE	616	NR	Routine blood counts, LDH	B	Blood count, chemistry	P	D + V	LDH, visceral involvement	60 (IQR 51-69)	5.5 months (pts alive) and 2.9 (died pts)	Pembrolizumab	L	L	L	L	L	L	L
Wen et. al, Cancer Immun.Immunother 2017 **[163]**	Retrospective case study (China)	52	Acral 42.3%; CSD or non-CSD 42.3%, mucosal 9.6% , uveal 5.8%.	LDH and relative lymphocyte count, plus relative and AWC	B	Blood count, chemistry	P + D	D	Performance status, liver metastasis, LDH	53 (20-78)	NR	Ipi (14), pembro (28), combination (10)	H	H	H	L	H	M	H
Wistuba-Hamprecht et. al, European J Cancer 2016 **[164]**	Retrospective cohort (US and Europe)	109	Cutaneous	Gamma-delta T cells (non MHC restricted T cells)	B	Flow cytometry	P + D	D	Number of metastasis, LDH	58 (28-89)	NR	Ipilimumab	M	L	L	L	M	H	M

Paper	Study design	N Melanoma patients	Type of melanoma	Biomarker of interest	Tumor (T) or blood (B)	Type of test	Timing of biomarker	Study phase	Adjustment for relevant confounders	Median age (range)	Median follow-up	Type of treatment	Quality assessment						
													**Risk of bias**	Participation	Attrition	Prognostic	Outcome	Adjustment	Statistics
Wistuba-Hamprecht et. al, European J Cancer 2017 **[165]**	Retrospective cohort (US and Europe)	137	Cutaneous	CD4 and CD8 T cells	B	Flow cytometry	P + D	D	N/A	58	15.9 months (alive pts), and 7.0 (died pts)	Ipilimumab	M	L	L	L	H	H	M
Wong et. al, J Immunother Cancer 2019 **[166]**	Retrospective cohort (US)	117	Non-uveal melanomas	Cancer associated fibroblasts (markers: Thy1, SMA, FAB)	T	Immunofluoresce nce	P	D	Age, sex, mutation status, stage, treatment, prior ICI	57% <65 years	NR	Anti-PD-1, anti-CTLA4 or a combination (ipi/nivo)	L	L	L	L	L	M	L
Wong et. al, Clin Cancer Res 2019 **[167]**	Retrospective cohort (US)	94	Non-uveal melanomas	TIL quantification (CD4, CD8, CD20) and TIL activation (CD3, GZMB, Ki67)	T	Immunofluoresce nce and cell count	P	D	Age, sex, mutation, stage, treatment, prior ICI	57% <65 years	NR	Anti-PD-1 Ab	L	L	L	L	L	M	L
Wood et. al, Genome Med 2020 **[168]**	Retrospective data from publicly available data	302	NR	TMB, RNA-derived variants and neoepitope burden	T	Whole genome sequencing, RNA variant identification	P	D	N/A	NR	NR	Anti PD-1 and anti-CTLA4 Ab. Dendritic cell vaccine.	M	H	M	L	M	H	M
Woods et. al, JCI Insight 2020 **[169]**	Retrospective trial (checkmate 063) (US)	NR	NR	Immunophenotypes of PBMC	B	Mass flowcytometry computational analysis approach to combine markers	P + D	D	N/A	NR	NR	13 weeks nivo, 13 weeks ipi, nivo OR 13 weeks ipi, 13 weeks nivo	H	M	H	L	M	H	H
Xiao et. al, EBioMedicine 2018 **[170]**	Retrospective selected from TCGA + Van Allen (US)	110	Skin (83.4%), mucosal (3.6%), occult (12.7)	TP53	T	Whole-exome sequencing	P	D	Tumor stage, LDH, TMB	61.5 (18-86)	NR	Ipilimumab	L	L	L	L	L	L	L
Yamazaki et. al, Cancer Science 2017 **[171]**	Prospective phase II single arm study (Japan)	35	Superficial spreading 22.9%, nodular, 12.9%, acral 25.7%, unknown 37.1%	IL, IFN-a2, IFN-y, TNF-B, monocyte chemoattractant proetin-1, stromal cell-derived factor 1A, IP10, MIG, g-CSG, platelet-derived + he-patocyte GF, VEGF-A	B	Human Cytokine Antibody Array	P	D	N/A	64 (28–79)	18 months	Nivolumab	H	H	H	L	L	H	H
Yoshida et. al, J ImmunoTher Cancer 2020 **[172]**	Prospective selected from CHECKMATE-064	95	NR	CRP	B	Magnetic bead-based assay	P	D	N/A	63 (19-84)	NR	Ipilimumab and anti-1 (both monotherapy)	M	M	M	L	L	H	H
Yuan et al, Pro c Natl Acad Sci USA, 2011 **[173]**	2 different cohorts	144	Stage IV (95%), stage III (5%)	NY-ESO-1 antibodies and T cell subsets	B	ELISA and T cell cultures	P + D	D	N/A	61 (32-86)	NR	Ipilimumab	H	L	H	L	L	H	H
Yuan et al, 2014, Cancer Immunol Res **[174]**	Retrospective cohort	176	98.7% stage IV disease.	serum VEGF	B	VEGF singleplex array plate	P + D	D	LDH, ALC	62 (16-91)	NR	Ipilimumab	M	L	H	L	L	M	H
Yusko et. al, Cancer ImmunRes 2019 **[175]**	Retrospective selected from CHECKMATE-064	91	NR	TMB, neo epitopes, TCR repertoire. PD-L1	B	IHC, NGS, immune-sequencing CDR3 regions of	P + D	D	N/A	NR	NR	Nivo followed by ipi or ipi followed by nivo, then nivo.	M	M	M	M	L	H	M

Paper	Study design	N Melanoma patients	Type of melanoma	Biomarker of interest	Tumor (T) or blood (B)	Type of test	Timing of biomarker	Study phase	Adjustment for relevant confounders	Median age (range)	Median follow-up	Type of treatment	Quality assessment						
													Risk of bias	Participation	Attrition	Prognostic	Outcome	Adjustment	Statistics
						human TCRβ chains. Flowcytometry.													
Zaragoza et. al, British J Dermatol 2016 **[176]**	Retrospective cohort (France)	58	Cutaneous, mucosal or choroidal	NLR, lymphocytes, neutrophils	B	Cell count	P	D	LDH, performance status, corticosteroid use	Mean 54,7 (SD 15,6)	931 days (IQR 251-997)	Ipilimumab	L	L	L	L	L	M	L
Zhou et. al, Cancer Immun Res 2017 **[177]**	Retrospective selected from trial for DC, VC not clear (US)	228	NR	Soluble PD-L1	B	Elisa, Immunnoblot	P + D	D + V	N/A	NR	NR	Ipi (with /without bevacizumab or sagramostin) or antiPD-1	M	L	M	L	M	H	M

Quality assessment: H: high, M: medium, L: low.
Abbreviations: AEC; absolute eosinophil count, ALC: Absolut lymphocyte count, ANC: absolute neutrophil count, AMC: absolute monocyte count, CRP: C reactive protein, ctDNA: circulating tumor DNA, CSD: chronic sun damage, CTC circulating tumor cells, D; during treatment, DC: development cohort, ESR: erythrocyte sedimentation rate, GEP: gene expressing profiling test, IL: interleukin, IPI: ipilimumab, LDH: lactate dehydrogenase, MC: monocyte count, MDSC: myeloid derived suppressor cells, MHC: major histocompatibility complex, NIVO: nivolumab, NLR: neutrophil to lymphocyte ratio, NR: not reported, N/A: not applicable, PBMC, peripheral blood mononuclear cells, PEMBRO: pembrolizumab. PLR: platelet to lymphocyte ratio, PS performance score, REC; relative eosinophil count, RLC: relative lymphocyte count, TMB: tumor mutational burden, TIL: tumor infiltrated lymphocyte, VC: validation cohort, WBC: white blood cell count.

Table S2 – Outcomes and interpretation of all included reports in systematic review

Paper	Response	PFS	OS	Interpretation/ conclusion
Amaral et. al. 2020	**Univariate:** P/LP associated with disease control (DC) (*P*=0.10, no OR calculated). Low/intermediate TMB associated with DC (OR 23.27, 95% C.I. 2.61-2077). Elevated S100B not associated with DC (OR 1.72, 95% C.I. 054-5.47), LDH not associated with disease control (OR 2.50, 95% C.I. 0.78-7.98).	**Univariate:** P/LP and TMB associated with PFS (HR 2.16 , 95% C.I. 1.01-4.64, *P*=0.48 and HR 2.88, 95% C.I. 1.12-7.38). S100B and LDH not associated with PFS (HR 0.992, 95% C.I. 0.55-1.81 and 1.26, 95% C.I. 0.71-2.26). **Multivariate:** P/LP and TMB associated with PFS (HR 1.93, 95% C.I. 0.89-4.15 and HR 1.75, 95% C.I. 1.07-7.09)	N/A	P/LP germline variants were predictive for PFS and MSS in multivariate analyses. Mutational burden was associated with PFS. LDH and s100B were associated with MSS but not PFS.
Arce Vargas et. al, 2019	**Univariate:** Higher response rate for high indel burden and the CD16-V158F SNP(*P*= 0.016) as well as nsSNV neoantigens and CD16-V158F (*P* = 0.043).	**Univariate:** Low TMB and high load of somatic mutations and SNP+ versus low load and SNP-, difference *P*=0.016. In patients with high mutational load: no significant difference.	**Univariate:** Low neoantigen burden + SNP-, low neoantigen burden + SNP+, high neoantigen burden + SNP-, high neoantigen burden + SNP+ (HR 0.247, 95% C.I. 0.074-0.826, *P*=0.014)	Germline DNA-mutations CD16a-V158F and CD32a-H131R SNPs in association wit low/high TMB were associated with response and survival, although in a small cohort with limited description of inclusion criteria and statistical analyses
Ascierto et. al, 2019	N/A	**Univariate 2-year PFS:** HR 0.61, 95% C.I. 0.37-1.02, *P*=0.060 for BDX008+ vs negative patients. **Multivariate:** HR 0.51, 95% C.I. 0.28-0.94 after adjustment for BRAF mutation, treatment line and LDH	**Univariate Cox regression for 2-year OS:** HR 0.50, 95% C.I. 0.29-0.88, *P*=0.016 for BDX008+ vs negative patients. **Multivariate:** HR 0.42, 95% C.I. 0.21-0.81 after adjustment for BRAF mutation, treatment line and LDH	BDX008 is associated with overall survival but not progression-free survival in univariate analyses. In multivariate analyses BDX008 and LDH were associated with both outcomes
Auslander et. Al, 2018	**Area under the curve (AUC)** for response was 0.97 for CTLA-4 and 0.81 for antiPD-1. Additional pooled dataset of 297 patients: AUC 0.83.	**Boxplot:** Higher IMPRES scores are associated with improved PFS (*P*=0.0009)	**Kaplan Meier curves:** Significantly different OS between low and High IMPRES (*P*=0.0074)	The IMPRES prediction score (based on transcriptomic data) predicts response and OS to both anti-CTLA and PD1 treatment
Ayers et. al, 2017	**Logistic regression:** Response *P*-values: IFN-y signature 0.047, preliminary expanded immune signature *P*=0.027.	**Cox regression:** PFS: *P*=0.016 for IFN-y signature and 0.015 for expanded immune signature	**Cox regression:** *P*-values 0.090 for IFN-y signature and *P*=0.105 for expanded immune signature.	Both signatures were predictive for response and PFS but not OS. No point estimates or effect sizes are reported in the paper. The

Paper	Response	PFS	OS	Interpretation/ conclusion
				paper performs analyses in other tumor types.
Babacic et. al, 2020	N/A	**Global proteomics**: **Univariate:** pre-treatment levels of 109 proteins were associated with PFS (*P*<0.05, no FDR), **Multivariate:** 22 pre-trm levels were associated with PFS. **Targeted proteomics**: **Univariate:** pre-trm plasma levels of 109 proteins were associated with PFS (*P*<0.05, FDR). **Multivariate:** 7 proteomics associated with PFS (*P*<0.05, 10% FDR;	N/A	109 proteins pre-treatment were associated with PFS, but only 7 proteins remained statistically significant in multivariate analyses.
Balatoni et. al, 2020	**Univariate**: no association between disease progression and any of investigated biomarkers (including LDH, ESR, AEC)	N/A	**Univariate:** diminished OS was associated with LDH > 1.5 × ULN (HR 6.565, 95% CI 2.695–15.996, *P*= 0.0029), ESR > 1 × ULN (HR 2.367, 95% CI 1.884–4.716, *P*= 0.0084), NLR ≥ 4. (HR 1.970, 95% CI 1.035–3.750, *P*= 0.0475), AEC > 0.1 G/L (HR 2.304, 95% CI 1.142–4.648, *P*= 0.0193), ELR > 0.1 (HR 6.105, 95% CI 1.302–25.628, *P*= 0.0401), PS >0 (HR 2.898, 95% CI 1.427–5.886,*P* =0.0153), and metastatic (HR 2.555, 95% CI 1.265–5.159, *P*=0.0300). **Multivariate:** LDH > 1.5 × ULN associated with OS (HR 3.554, 95% CI 1.225–10.306, *P*= 0.0190),	Significant association between high LDH level (>1.5× upper limit of normal) and decreased overall survival was demonstrated in uni- and multivariate analysis
Balatoni et. al, 2018	**Lymph node metastases:** CD4+ (*P* = 0.0274), CD8+ (0.0106), FOXP3+ (0.0009), CD134+ lymphocytes (0.0480), CD20+ B cells (0.0274), and NKp46+ NK cells (0.0274) correlate with response. **Cutaneous metastases:** CD68+ macrophages (0.0094) and CD16+ cells (0.0373) were associated with response. All samples together: association with response was found in NK cell density values (*P* = 0.0182;), NK cells (0.0232), FOXP3+ (0.0371) and CD68+ macrophages (0.0344)	N/A	**Lymph nodes: Univariate analysis:** CD4+, CD8+, CD45RO+, FOXP3+, and CD16+ cell densities associated with OS (*P*=0.0290, *P*=0.0093, *P*=0.0180, *P* =0.0083, and p =0.0047), ECOG status (*P*=0.0009) and LDH (*P*=0.0227). **Multivariate: E**COG (*P*= 0.001) FOXP3+ density (*P*= 0.004) predictors of OS. **Cutaneous: Univariate:** CD16+ and CD68+ cells showed correlation with OS (*P*=0.0197 and *P*=0.0175) **Multivariate:** LDH level predicts OS (*P*=0.002). **All samples: Univariate:** CD4, CD8, CD45RO, FOXP3, CD16, CD68,, CD20 (*P*=0.0348, *P*=0.0136, *P*=0.0113, *P*=0.0121, *P*=0.0055, *P*=0.0168, or *P*=9 0.0372), ECOG (*P*=0.0026) LDH (*P*= 0.0006) associated with OS**.** **Multivariate:** LDH (*P* =0.001), FOXP3+ cells (*P*=0.016) predict OS.	In this small study, infiltration by FOXP3+ cells, CD4+, CD8+, CD134+ T lymphocytes, CD20+ B cells, and NKp46+ NK cells in lymph node metastases, as well as the prevalence of CD16+ cells and CD68+ macrophages in cutaneous/subcutaneous metastases could be considered as candidate predictive markers
Barak et. al, 2015	Higher levels of S-100β pre-therapy, (5.1+0.7 µg/L) that decreased post- therapy (1.3+0.4 µg/L) were seen in responding patients (No statistical analyses)	N/A	Patients with high S-100b levels: 17% was alive after 2 years, compared to 58% of patients with low S100-B levels.	Higher levels of S-100β pre-therapy, that decreased post- therapy, were seen in responding patients, but no formal statistical analyses were reported.
Bartlett et. al, 2020	**2-Year TTF: Univariate:** NLR associated with prolonged TTF (HR 1.65, 95% CI 1.19-2.29, *P*= .003), **Multivariate:** NLR associated with TTF (HR 1.73, 95% CI 1.24-2.41, *P*=.001**).** **Landmark analysis:** Baseline NLR <5 and increase <30% during first 2 cycles associated with prolonged TTF.	N/A	**Univariate**: elevated baseline NLR (+ mucosal melanoma, ECOG, AJCC, metastatic sites) associated with OS (HR 2.05, 95% CI 1.42-2.97, *P*=<.001). **Multi-variate**: mucosal melanoma, metastases, NLR ≥5 associated with OS (HR 1.95, 95% CI 1.33-2.86, *P*=0.001).	Baseline elevation in the NLR was independently associated with shorter TTF and shorter OS
Bence et. al, 2020	N/A	N/A	**Whole group: Multivariate:** PD-L1 AND CD8+TILs: PD-L1-/CD8+TILS+ had better OS (*P*=0.041). OS differed 72% for PD-L1-TIL+ group vs 50% in other three groups combined. CD28 not predictive for OS. Ki67 was poor prognostic for OS *P*=0.02, no effect size). **ICI alone: Multivariate:** PD-L1+/ TILs+ status combined were associated with OS (PD-L1+/TILs+ status; HR, 0.138; 95% CI, 0.024–0.779; *P* = 0.022). OS differed 66% for PD-L1-	Combination of lacking PD-L1 expression on tumors with CD8+TILS was favorable for OS. Ki67 was a poor prognostic factor for OS. CD28 was not associated with OS.

Paper	Response	PFS	OS	Interpretation/ conclusion
			TIL+ group vs 53% in other groups combined. (66%, 8/ 12 of patients alive vs. 53%, 21/39; *P* = 0.034)	
Bjoern et. al, 2016	**Baseline:** MDSC's (Lin-HLA-DRlow/CD14+CD11+CD33+ correlated with response (no *P*). Trend towards lower IL6 in responders (*P*=0.09). **During treatment:** ALC increased in responders but not non-responders (*P*=0.008). No changes in CD4/CD8 distribution. No changes in NK cells or cytokines or other changes in responders.	N/A	**Kaplan Meier:** Only IL6 was tested in association with survival and was not significantly associated.	During treatment, changes in ALC were associated with response. Baseline MDSC's and IL-6 were predictive of response. No validation was performed. The other marker were not predictive of response.
Bochem et. al, 2019	**Univariate:** CD56+ was not associated with response. PD1 was predictive of response within patients with CD56+ T-cells (*P*<0.01).	**Univariate:** CD56 was not associated with PFS. PD1 was predictive of OS within CD56+ T-cells (*P*=0.041).	**Univariate:** CD56 not associated with OS. PD1 was predictive of OS within CD56+ T-cells (*P*=0.004). **Multivariate**; PD1+CD4+T cells and PD1+CD8+T-cells) PD1 was predictive for OS within CD56+ T cells (HR 2.39 *P*=0.028). PD-1+CD56+ frequencies (HR, 2.39; *P*= 0.028) were predictive for OS.	CD56 on T-cells was not associated with response, PFS or OS. Within CD56+ T-cell populations, PD1 was predictive for these three outcomes.
Bruggemann et. al, 2017	**Univariate:** PD-L1 on mRNA level was not associated with response (*P*=0.458)	N/A	N/A	PD-L1 on mRNA level was not associated with response to ipilimumab.
Cabrita et. al, 2020	N/A	N/A	**Cox regression:** TLS-high tumors associated with OS after CTLA4 blockade (*P*=0.05, n = 37; *P*=0.045, *N*=40) and anti-PD1 (*P*=0.0012, *N*=69; *P*=0.014, *P*=0.014)	Tertiary lymphoid structures were associated with OS in anti-CLTA4 and anti-PD1 in a univariate analysis.
Campesato et. al, 2015	**Mann-Whitney**: There was no significant association between mutational loads and durable clinical benefit (P = 0.37 and p=0.24).	N/A	N/A	CGP-mutational load is not associated with clinical benefit to CTLA-4 blockade therapy
Capone et. al, 2018	N/A	**Univariate:** LDH (2.16 (1.18–3.99) *P* = 0.01), NLR (2.53 (1.53–4.18) *P* < 0.0001) and dNLR (2.50 (1.48–4.23) *P* = 0.001) were prognostic for PFS. **Multivariate:** associations observed for NLR (HR = 2.10; 95% CI: 1.23–3.59; *P* = 0.007) and LDH (HR = 1.74; 95% CI: 1.03– 2.94; *P* = 0.04)	**Univariate:** increasing ANC (2.04 (1.17–3.57), *P* = 0.01), LDH (3.06 (1.69–5.57), *P* < 0.0001), NLR (3.53 (2.02–6.16), P < 0.0001) and dNLR (2.70 (1.55–4.69), *P* < 0.0001) associated with OS. **Multivariate:** NLR (HR 2.85; 95% CI 1.60-5.08; *P* <0.0001) LDH (HR 2.51; 95% CI 1.36-4.64; *P*<0.0001) associated w. OS.	Baseline neutrophil-to-lymphocyte ratio (NLR) and derived NLR could predict overall survival and PFS in patients with advanced melanoma treated with nivolumab
Capone et. al, 2020	Patients with clinical benefit showed lower pre-treatment median frequency of circulating CD8+PD-1+CD73+lymphocytes (0.85%) than patients who progressed to nivolumab treatment (3.02%) (*P*=0.02). However, during treatment there were no differences in changes of these cells.	Pre-treatment frequency of CD8+PD-1+CD73+lymphocytes was significantly associated with progression-free survival (PFS), but formal analyses were reported in supplemental files that were not available.	**Univariate:** CD8+PD-1+ lymphocytes < 9.8% (1.86 (1.17–2.98) *P*< 0.009) and CD8+PD-1+CD73+ lymphocytes < 2.3% (2.25 (1.39–3.63) *P*< 0.001) associated with OS, CD8+T cells positive only to CD73 not associated with OS (1.55 (0.97–2.48) *P*=0.07). **Multivariate:** CD8+PD1+CD73+ lymphocytes < 2.3% associated with OS [HR 2.17 (1.34– 3.51); *P*=0.002].	Pre-treatment frequency of CD8+PD1+CD73+T cells was associated with OS and clinical benefit to nivolumab.
Carlino et. al, 2018	**PD-L1–positive tumors:** ORR was 39.9% (95% CI, 35.3%–44.6%) with pembrolizumab and 13.8% (95% CI, 9.6%–19.0%) with ipilimumab. **PD-L1–negative population:** ORR was 24.3% (95% CI, 16.4%–33.7%) with pembrolizumab and 12.8% (95% CI, 4.8%–25.7%) with ipilimumab	**PD-L1–positive tumors**: median PFS was 6.6 months (95% CI, 4.2–9.7 months) for pembrolizumab and 2.9 months (95% CI, 2.8–3.0 months) for ipilimumab. **PD-L1–negative tumors: PFS is** 2.8 months (95% CI, 2.8–3.7 months) for pembrolizumab and 2.8 months (95% CI, 2.6–3.0 months) for ipilimumab	**PD-L1–positive tumors:** median OS was 35.6 months (95% CI, 29.3 months to NR) with pembro and 17.1 months (95% CI, 13.0–26.2 months) with ipi. **PD-L1–negative tumors:** OS was 17.2 months (95% CI, 10.3–25.9 months) for pembro and 13.7 months (95% CI, 7.5–22.6 months) for ipi.	PD-L1 might be associated with a lower response PFS and OS to pembrolizumab, but "sample size was not powered to detect a difference for this subgroup." No formal analyses between groups were presented.
Cassidy et. al, 2017	**Univariate:** NLR ≥ 5 at any timepoint, either before or during treatment, was significantly associated with lack of clinical benefit at 12 weeks. **Multivariate:** remained significant (*P*<0.001)	**Univariate:** NLR ≥ 5 at all-time points plus stage IV M1C disease, mucosal melanoma and Karnofsky performance status score < 90 associated with worse PFS. **Multivariate:** NLR ≥ 5 at every timepoint associated with poor PFS (HR 1.81–2.51).	**Univariate**: NLR ≥ 5 at all time points + stage IV M1C disease, mucosal melanoma and Karnofsky performance < 90 associated with significantly worse OS. **Multivariate:** NLR ≥ 5 at every timepoint associated with poor OS (HR 2.03–3.37)	A high NLR, whether measured prior to or during treatment with ipilimumab, is associated with worse OS, PFS, and clinical response. An increasing NLR from baseline during treatment was correlated with worse OS and PFS.
Chakravarti et. al, 2017	**OS after relapse: Univariate:** High CTLA-4 expression in peritumoral lymphocytes had high risk of death (HR=3.695, 95% CI=1.047–13.041; *P*=0.042). **Multivariate:** High CTLA-4	**Univariate:** High % of cells positive for CTLA-4 (HR =2.172, 95% CI =1.111–4.249; *P* =0.023) had shorter PFS + high FasL expression had poor PFS (HR =4.176,	**Univariate:** Poor OS was seen in high CTLA-4 expression (HR =2.255, 95% CI=1.134–4.485; *P*=0.02) + high counts of CTLA-4 immunopositive peritumoral lymphocytes (HR	High CTLA-4 and p-Akt expression in tumor cells or peritumoral lymphocytes

Paper	Response	PFS	OS	Interpretation/ conclusion
	and FasL had poor OS (HR=6.145, 95% CI=1.073–35.209; *P*=0.041) + peritumoral lymphocytes expressing both p-Akt and FasL had shortest OS (HR=5.797, 95% CI=1.05–32.017; *P*=0.044).	95% CI= 1.275–13.676; *P*= 0.018). **Multivariate:** CTLA-4 and p-Akt had worse PFS (HR =4.842, 95% CI=1.383–16.959; *P*=0.014) + immunopositivity for p-Akt and p-S6 had shorter PFS (HR =3.058, 95% CI=1.079–8.669; *P*=0.035).	=2.436, 95%CI=1.129–5.255; *P*=0.023). **Multivariate:** high CTLA-4 and p-Akt correlated with poor OS (HR = 3.664, 95% CI =1.068–12.571; *P* =0.039).	correlated with a poor response to ipilimumab and a worse PFS and OS.
Chasseuil et. al, 2018	N/A	**Univariate:** leukocyte count (1.16 [1.07; 1.24] *P*=0.01), LLR (1.2 [1.1; 1.30]*P*=0.01), neutrophil count (1.18 [1.1; 1.27] *P*=0.01), NLR (1.09 [1.03; 1.14] *P*=0.01), monocyte count (6.33 [2.4; 16.69] *P*=0.01), LDH (1.25 [1.13; 1.38] p=0.01) and CRP (1.01 [1.01; 1.02] *P*=0.01) were associated with decreased PFS. **Multivariate:** increased MC (*P*=0.04; HR 3.5; 95% CI, 1.01–12.1) was associated with PFS.	**Univariate:** WBC (*P*=0.01; HR 1.13; 95% CI, 1.06–1.22), LLR (*P*=0.01; HR 1.11; 95% CI, 1.04–1.17), ANC (*P*=0.01; HR 1.16; 95% CI, 1.08–1.26), NLR (*P*= 0.01; HR 1.08; 95% CI, 1.02–1.15), AMC (*P*=0.01; HR 4.31; 95% CI, 1.46–12.74), LDH (*P*=0.01; HR 1.31; 95% CI, 1.18–1.45) and CRP (*P*=0.01; HR 1.01; 95% CI, 1.01–1.02) associated with decreased OS. Elevated AEC (*P*=0.04; HR 0.01; 95% CI, 0.01–0.86) associated with increased OS. **Multivariate:** LLR (*P*=0.02; HR 1.11; 95% CI, 1.02–1.21), NLR (*P*=0.02; HR 1.12; 95% CI, 1.02–1.23) and MC (*P*=0.01; HR 6.31; 95% CI, 1.5–26.59) were associated with poor OS.	Increased AMC, leukocyte/lymphocyte ratio and NLR were associated with decreased OS after bi- and multivariate analyses. Increased AMC was significantly associated with decreased PFS.
Chat et. al, 2019	**Anti-CTLA-4:** Dominant logistic model: for rs1893217 carriers of at least 1 copy of minor allele G were more likely non-responders, compared to homozygous reference genotype (OR 2.79, 95%CI= 1.36-5.73; *P*=0.005), but adjusted for Bonferroni or Holm-Bonferroni methods not significant (*P*-adjusted = 0.09) **Anti-PD-1:** Dominant logistic model: rs17388568 associated with response (OR=0.26; 95%CI=0.12-0.53; *P*=0.0002;) adjusted for Bonferroni. **Combination therapy:** No significance. **Pooled analysis:** no significance	N/A	N/A	rs17388568, a risk variant for allergy, colitis and type 1 diabetes, was associated with increased anti-PD-1 response, with significance surpassing multiple testing adjustments.
Chen et. al, 2018	High circulating exoPD-L1 prior to treatment associated with poor clinical outcome (*P*=0.0018) Responders had greater increase of exosPD-L1 after 3–6 weeks (*P*=0.000001). Total PD-L1, microvesicle PD-L1, extracellular vesicle-excluded PD-L1 did not differ between response /no response. ORR for high and low pre-treatment exoPD-L1:14/18 responders in high levels and 7/26 responders in low levels (*P*=0.0018).	N/A	N/A	Higher circulating exosomal PD-L1 before treatment was associated with poor response, an increase in circulating exosomal PD-L1 during treatment was associated with response.
Chen et. al, 2019	N/A	**Univariate**: MSS for LRP1B mutations vs wild type: *P*=0.0051. **Multivariate**: HR 0.63 (0.40-0.97,*P*=0.037) for LRP1B mutation vs wildtype	N/A	LRP1B mutations were prognostic for melanoma-specific survival after adjusting for age, sex, stage ant tumor mutational burden
Chen et. al, 2019	Neoantigen load was not associated with response. Higher somatic tumor mutation load was associated with a better response (*P*=0.0347).	N/A	Neoantigen load was associated with overall survival (HR 0.56, 95% C.I. 0.38-0.82, *P*=0.003) in multivariate cox regression.	Neoantigen load was associated with OS in multivariate analysis. A high tumour mutational burden was associated with better response.
Chen et. al, 2016	**Pre-treatment**: CD8, CD4 + myeloid markers not predictive of response to anti-CTLA4. CD8, CD3 + CD45RO+T-cells associated with response in patients treated with anti-PD1 (*P*=0.03, 0.03 and 0.02). GEP not associated with response. **Early on-treatment:** Association with response for CD8 (*P*=0.001), CD4 (p=0.001) and CD3 (*P*<0.001) on T-cells, and PD-1 (*P*<0.001), PD-L1 (*P*=0.007) and LAG3 (*P*<0.0001), FOXP3 (*P*<0.001) and granzyme B (*P*=0.02). 411 differentially expressed genes associated with response (*P*<0.05). 17 shared DEGs upregulated for CTLA4 and PD-1 Ab, with 56	N/A	N/A	Early on-treatment immune markers including CD8, CD3, CD45RO, PD1, PD-L1, LAG3, FOXP3 and granzyme B were associated with response to anti-PD1 but not CTLA4. Early on-treatment GEP associated with response to CLTA-4 Ab.

Paper	Response	PFS	OS	Interpretation/ conclusion
	upregulated DEGs unique to CTLA4 Ab and 253 to PD-1(FDR-adjusted *P* < 0.05;)			
Chew et. al, 2019	**Anti-CTLA-4:** non-responders to anti-CTLA-4 had higher DUX4 transcriptional activity relative to responders. (*P*=0.029). **Anti-PD-1:** No significance.	Stratifying patients according to DUX4 transcriptional activity revealed differences in PFS following anti-CTLA-4 (*P*=0.012). No significance in anti-PD1 cohort.	Stratifying patients according to DUX4 transcriptional activity showed differences in OS for anti-CTLA-4 therapy. (*P*=0.039) No significance in anti-PD1 cohort.	DUX4 is associated with failure to respond to anti-CTLA-4 therapy but no association was found to anti-PD1. There was no correction for confounders.
Cho et. al, 2016	**Positive PD-L1 immunostaining (cutoff 1%):** ORR was 20 % (2/10 patients) and DCR was 80 %. **Negative PD-L1 immunostaining (cutoff 1%):** ORR was 12.5 % (1/8 patients) and DCR was 37.5 % (3/8 patients). **Positive PD-L1 immunostaining (5 % cutoff):** ORR was 33.3 % (2/6 patients) and the DCR was 83.3 % (4/6 patients). **Negative immunostaining (5% cutoff):** ORR was 8.3 % (1/12 patients) and the DCR was 50 % (6/12 patients) (*P*=.180, p.171)	PFS was 6.2 months (range, 0.7–8.5 months) in positive group and 1.6 months (range, 1.4–9.5 months) in negative group (*P* = 0.137), **cutoff 1%**. PFS was 6.8 months (range, 0.7–8.5 months) in positive group and 1.9 months (range, 1.4–9.5 months) in negative group, (*P* = 0.149), **cutoff 5%**.	N/A	The immunohistochemical PD-L1 expression seemed to be correlated with better clinical outcomes of anti-PD-1 in limited cases, but PD-L1 expression cannot be considered a definitive biomarker for the response to ICIs.
Chow et. al, 2019	**Wilcoxon:** Responders had high CXCL9 + CXCL10 within first months after ICI (*P*<0.001, *P*<0.001 resp)	N/A	N/A	An increase in CXCL9 and CXCL10 plasma levels early after IC is a positive indicator of response.
Chowell et. al, 2018	N/A	N/A	**Cox regression:** HLA- B44 superfamily had better OS (*P*=0.01, HR 0.61, 95% CI 0.42-0.89) and patients with B62 alleles had reduced survival (*P* = 0.0007, HR = 2.29, 95% CI 1.40 to 3.74). **Cohort 1:** HLA-B44 types better OS in univariate (HR 0.5 (0.32-0.76, *P*=0.001) ad multivariate (0.23,0.13-0.41, *P*<0.001). HLA-B*15:01, HLA-B*07:02, and HLA-B*53:01-, associated with decreased OS (HLA-B*15:01: (HR 2.21, 1.33-3.67, *p*=0.002).**Cohort 2:** HLA-B44 supertype alleles better OS survival on univariate (P = 0.054, HR = 0.32, 95% CI 0.09- 1.1) and multivariable analysis (*P*=0.023, HR=0.13, 95% CI 0.02-1.03).	Melanoma patients with the HLA-B44 supertype had extended survival, whereas the HLA-B62 supertype (including HLA-B*15:01) or somatic loss of heterozygosity at HLA-I was associated with poor outcome
Cordonnier et. al, 2020	In complete and PR (n = 27), ΔExoPD-L1 decreased (mean S1: 121.06 ± 26.65 vs mean S2: 104.78 ± 17.11,*P* = 0.8607). In patients experiencing progression (n = 9), ΔExoPD-L1 increased significantly (mean S1 85.90 ± 24.46 vs mean S2 344.20 ± 70.30 *P* = 0.0002)	**Univariate**: ΔExoPD-L1 was associated with PFS (HR: 1.003, CI 95% = 1.001 − 1.006; *P* = 0.006)	**Univariate:** ΔExoPD-L1 was associated with OS (HR: 1.004, CI: 95% = 1.001–1.008, *P* = 0.034)	ExoPD-L1 can be used as a predictor of treatment response in melanoma patients receiving immunotherapy or targeted therapy.
Cristescu et. al, 2018	**Univariate:** TMB and T cell inflamed GEP scores associated with BOR (P < 0.05). **Multivariate:** GEP remained significant (TMB *P* = 0.1644 and GEP *P* = 0.026).	Median PFS for TMB-high vs TMB-low 502 vs 85 (0.48; 0.28 to 0.84); for GEPhi vs GEPlo were 418 vs 90 (0.73; 0.40 to 1.31); those for TMBhi GEPhi vs TMBlo GEPlo or TMBlo GEPlo were 504 vs 123 (0.63; 0.36 to 1.09).	N/A	Limited clinical responses to pembro occurred in patients with low levels of TMB and T cell inflamed GEP, whereas best response rates were seen in patients with high levels of biomarkers.
Daud et. al, 2016	**Development cohort:** ORR was 0% (0 of 6) in low CTLA-4hiPD-1hi CTL group vs 85.7% (12 of 14) in >20% CTLA-4hiPD-1hi CTL group. **Validation:** ORR was 78.6% for high CTLA-4hiPD-1hi CTL group vs 0% (0 of 6) for low CTLA-4hiPD-1hi group.	**DC:** For >20% CTLA-4hiPD-1hi CTLs, PFS was 31.6 months vs 9.6 months for 20% or fewer CTLA-4hiPD-1hi cells within the CTL gate (*P*=0.017). **VC:** patients <20% tumor-infiltrating CTLA-4hiPD-1hi CTLs had PFS of 9.9 months vs15.9 months for high CTLA-4hiPD-1hi CTL	The increased proportions of CTLA-4hiPD-1hi CTLs also correlated with OS; however, this did not achieve statistical significance during the relatively short study period	Relative abundance of partially exhausted tumor-infiltrating CD8+ T cells might predict response and PFS to anti-PD-1 therapy in a development and validation cohort.
Daud et. al, 2016	An association between a higher MEL score for PD-L1 expression and ORR was observed (*P*= .001). Highest and lowest ORRs were observed in patients with tumors with a MEL score of 4 (ORR, 57%) and 0 (ORR, 8%).	Associations between higher PD-L1 MEL score and PFS were observed (HR 0.76; 95% CI, 0.71-0.82; *P*=.001). PD-L1 expression categorized as positive (i.e., MEL score 2 -5) or negative (HR 0.51 (95% CI, 0.40-0.65) for PFS.	Associations between higher PD-L1 MEL score and OS (HR, 0.76; 95% CI, 0.69 to 0.83; *P*=.001) were observed. PD-L1 expression categorized as positive or negative HR 0.50 (95% CI, 0.37- 0.67) for OS.	PD-L1 expression in pretreatment tumor biopsy samples was correlated with response rate, PFS, and OS; however, patients with PD-L1–negative tumors may also achieve durable responses

Paper	Response	PFS	OS	Interpretation/ conclusion
de Coana et. al, 2017	**Monocytic MDSCs:** Patients with clinical benefit showed reduction of MoMDSCs to the first ipilimumab dose, at the three-week time point. **CD8 T cells:** patients with clinical benefit had significantly higher effector memory cells at the end of treatment when compared to patients with progressive disease	N/A	**CD8:** High CD8 EM cell frequencies above 30.05% at 9 weeks had a MOS of 80 weeks vs 34 weeks in low CD8 EM (HR 0.21 (0.08-0.56), *P*=0.000063). **MoMDSCs:** No significant differences in OS observed at baseline and 9 weeks (data not shown). At 3 weeks, MoMDSCs showed to be inversely correlated with OS (HR 2.89, (1.59-6.99, *P*=0.000023).	Changes in MoMDSCs and CD8 EM T cells during treatment are associated with response to treatment and OS.
Del Re et. al, 2018	**T-test**: mRNA copies/ml of PD-L1 in plasma-derived exosomes decreased in CR/PR (mean 793.5 vs 231, time 0 vs 2 months), while it increased in patients with PD (mean 143.3 vs 326.7, time 0 vs 2 month,).	N/A	N/A	PD-L1 levels in plasma-derived exosomes decreased in patients responding to treatmen and increased in subjects with disease progression, but no significance was found.
Delyon et. al, 2013	N/A	N/A	**Univariate:** An increase >100/mm3 in AEC associated with longer OS (median OS 11.3 months vs 6.8 months, *P*=0.012) + increase >100% in AEC between first 2 ipi courses (*P*=0.062). Normal LDH level before first ICI associated with better OS (*P*=0.003). **Multivariate:** number of ipi courses and ALC at start of 2nd course associated with OS (HR 2.0; 95% CI 1.1–3.9), (HR 3.7; 95% CI 2.0–6.8).	ALC >1000/mm3 at the start of the second course and an increase in the AEC >100/mm3 between the first and second infusion and normal LDH correlated with improved OS.
Di Giacomo et. al, 2013	Among patients who achieved disease control, the increase in both CD4+ ICOS+ and CD8+ICOS+ T cell counts at weeks 7 and 12 was more than fivefold.	N/A	**ICOS expression:** Better OS were observed in increase at W7 in CD4+ICOS+T cells [HR 0.14; 95 % CI 0.02–1.25; *P* = 0.009] or CD8+ICOS+ Tcells (HR 0.11; 95 % CI 0.01–1.01; *P* = 0.02). **N/L ratio:** N/L< median values at W7 and W10 had better OS compared with N/L > median value (HR 2.81 at W7: 3.86, 95 % CI 1.43–10.41, *P*=0.004; HR 3.38 at W10: 6.57, 95 % CI 2.22–19.50, *P*=0.0001).	In a limited sample size, changes in the number of circulating ICOS T cells or N/L ratio during ipilimumab treatment may represent early markers of response and OS.
Diem et. al, 2018	**Fisher's exact test**: Correlation between the presence and the absence of TILs in responders vs. non-responders (*P* = 0.008).	PFS shows a trend in favor of the patients having TIL rich lymph node metastases (6.8 vs. 3.3 months, *P*= 0.09).	OS shows a trend in favor of the patients having TIL rich lymph node metastases (all alive at last follow-up vs. 8.2 months, respectively = 0.08).	In 9 patients, TILs in lymph nodes may predict response to ipilimumab but not OS o PFS. TILs in the primary tumor did not predict response.
Diem et. al, 2015	N/A	N/A	**Univariate**: LDH, ECOG, NLR, albumin, NOI and metastasis associated with OS. **Multivariate:** Elevated LDH (HR1.03, 95% CI 1.02-1.04, *P*=0.000), ECOG status >0 (HR 1.91, 95% CI 1.10-3.30, *P*=0.06), and n. organs involved (HR 1.51, 95% CI 1.22-1.86, *P*=0.005) associated with OS.	LDH, ECOG performance status and NOI were identified as the most relevant significant independent prognostic factors for OS.
Diem et. al, 2016	Almost all patients showing PD (*N*=15) had LDH increase compared with baseline (mean change þ 38.9%, SD ±44.1, range 8.5-131.1%), whereas patients with SD (*N*=3) had a mean LDH change of 8.0% (SD±5.1, range □13.9-4.8%) (ANOVA: *P*=0.0001).	N/A	Elevated baseline LDH (N 1⁄4 34) had a shorter OS normal LDH (N=32; 6-month OS: 60.8% vs 81.6% and 12-month OS: 44.2% vs 71.5% (log-rank *P*=0.0292). Relative increase over 10% from elevated baseline LDH had a shorter OS compared with patients with p10% change (4.3 vs 15.7 months, log-rank *P*=0.00623).	Increasing LDH during first weeks of anti-PD-1 can predict disease progression before the first scan and is associated with decreased survival. Elevated LDH at baseline is associated with a shortened survival.
Diem et. al, 2019	IgG2 levels before initiation of CI therapy were higher in responder group vs nonresponder-group (*P*=0.011).	PFS was better in patients with high levels of total IgG [>9.66 g/L, HR=0.43, CI: 0.18–0.98, *P*=0.038], IgG1 (>6.22 g/L, HR=0.32, CI: 0.11–0.92, *P*=0.025), IgG2 (>2.42 g/L, HR=0.41, CI: 0.19–0.88, *P*=0.019), and IgG3 (>0.21 g/L, HR=0.45, CI: 0.21–0.96, *P*=0.034)	OS was significantly prolonged in patients with IgG2 levels >2.42 g/L (HR=0.41, CI: 0.17–1.00, *P*=0.043)	Baseline serum immunoglobulin levels may serve as predictive markers for response to CI therapy. Furthermore, a prognostic association of IgG subclass levels and survival was demonstrated.
Dupuis et. al, 2018	Better ORR associated with metachronous metastases (P = 0.04), PD-L1 tumour- and immune-cell status (*P* = 0.01), CD163+ histiocytes at advancing edges (*P* = 0.009) NRAS mutation (P = 0.019).	**Univariate:** CD163+ histiocytes at advancing edges (*P* = 0.04) and metachronous metastases (*P* = 0.049) were associated with PFS	**Univariate:** Metachronous metastases is associated with longer OS (*P* = 0.02).	PD-L1 status alone cannot be used as a reliable biomarker of response, but could be combined with metachronous metastases, >

Paper	Response	PFS	OS	Interpretation/ conclusion
				10% CD163+ histocytes at advancing tumour edges or NRAS.
Edwards et. al, 2018	A trend toward a greater magnitude of expansion of these cells in responding patients (*P* = 0.07) compared with nonresponder (*N* = 8).	N/A	N/A	Tumor-resident CD8+ T cells are better predictors of MSS than total CD8+ T cells in patients who are immunotherapy naive
Failing et. al, 2017	N/A	Median PFS was 5.5 months [95% CI 4.1–7]. Median PFS for patients with LMR> 1.7 was 6.3 months (95% CI: 4.8–9.7) vs 2.8 months (95% CI: 2.2–5.4) for LMR<1.7. **Multivariate:** LMR> 1.7 correlated with improved PFS [HR 0.55; 95% CI: 0.34–0.92; *P*=0.024]	Median OS for patients with LMR of at least 1.7 was 28.2 months (95% CI: 20.8 months – not reached) vs 12.4 months (95% CI: 4.7–18.9) for LMR of less than 1.7. **Multivariate:** LMR of at least 1 correlated with improved OS (HR= 0.29; 95% CI: 0.15–0.59; *P*=0.0007	LMR above 1.7 was significantly associated with increased PFS and OS in multivariate analysis
Fairfax et. al, 2020	Responders had more circulating large CD8+ clones (*P* healthy control=0.003, *P* non-responders=0.037). **Random effects model**: Responders had on average 5.7 more large clones on day 21 (95% CI 2.6–8.87, *P*=4.8×10−4;)	PFS was significantly different between patients with day21 large clone count below and above the median for the cohort. (*P*=0.003)	OS was significantly different between patients with day21 large clone count below and above the median for the cohort. (**P**=0.019)	Responders have more large clones (>0.5% of repertoire) post-treatment than non-responders. 6-month clinical response is associated with large CD8+ T cell clone count 21 days after treatment and agnostic to clonal specificity.
Fässler et. al, 2019	**Wilcoxon:** Better response with antibodies at baseline: NY-ESO1 (*P*=0.005), TRP1 (*P*=o.048), MelanA (*P*=0.015), TRP2 (*P*=0.047). GP100 not associated. Changes during treatment in antibodies not associated.	High melanA and gp100 at baseline were associated with longer PFS (*P*=0.11 and 0.014 respectively), the other markers were not associated with PFS	**Univariate:** OS was better for patients with the following biomarkers in baseline samples: NY-ESO1 (*P*=0.037), MelanA *P*=0.001), anti-gp100 (*P*=0.031) but not for TRP1 and TRP2.	High MelanA at baseline was predictive for response, PFS and OS, although there was no correction for confounders. The other markers showed discrepant results for these outcomes.
Felix et. al, 2016	**Univariate:** Low LDH before+ during treatment associated with better response (*P*=0.003 for baseline, *P*=0.003 after 3 wks and *P*=0.004 after 4wks). Low s100 at baseline was associated with response (*P*=0.03), after 3wks (*P*=0.05) and 6wks (*P*=0.001). Other biomarkers not associated. **Multivariate:** only LDH predictive.	N/A	**Univariate**: LDH and s100 predictive for OS before and during treatment (LDH: *P*=0.001, 0.050 and 0.001 at 0, 3 and 6 wks, s100: *P*=0.011, *P*=0.007 and *P*=0.028 respectively). **Multivariate:** Only LDH remained significant (*P*=0.03). MIA and anti-MICA were not associated with OS.	LDH was predictive for response and overall survival. s100 was not independently associated with these outcomes, and MIA and anti-MICA antibodies were not predictive for outcome.
Felix et. al, 2015	**Univariate**: Baseline ALC predictive for DC (*P*=0.04). Increase in ALC associated with DC (*P*=0.013). Rise in CD4+ TCM and TEM was higher in patients with DC.	N/A	Baseline ALC was predictive for OS in **univariate** (*P*=0.003) and **multivariate analyses** (HR 0.49 95% C.I. 0.28-0.86).	Baseline and changes in ALC were associated with disease control and overall survival in both univariate and multivariate analyses.
Ferruci et. al, 2016	N/A	**ANC**: Median PFS 4.0 (95% CI 3.5–4.5) months for patients with normal ANC compared with 1.7 (95% CI 1.0– 2.3) months for elevated ANC (P < 0.0001). **Multivariate**: ECOG PS 1-2, brain/liver mets and neutrophilia associated with worse PFS. Raised ANC associated with increased risk of progression (HR = 2.52; 95% CI 1.97–3.21; *P* < 0.0001). **dNLR:** Median PFS 4.3 (95% CI 3.8–5.2) months for patients with dNLR < 3, and 2.4 (95% CI 2.1–2.8) for patients with higher dNLR (*P*< 0.0001). **Multivariate:** ECOG PS > 0, brain/ liver mets and raised dNLR associated with worse PFS. dNLR ≥ 3 associated with risks of death (HR=2.29; 95% CI 1.86–2.82; *P*<0.0001) + progression (HR = 2.03; 95% CI 1.66–2.47; *P* < 0.0001).	**ANC:** Median OS 8.7 (95% CI 7.7–9.8) months normal ANC, compared with 2.3 (95% CI 1.5–3.1) months for elevated ANC (*P*< 0.0001). **Multivariate:** ECOG PS 1-2, brain and liver mets and neutrophilia associated with worse OS. Raised ANC associated with increased risks of death (HR = 3.38; 95% CI 2.62– 4.36; *P* < 0.0001). **dNLR:** Median OS 9.2 (95% CI 8.2–10.4) months for patients with dNLR < 3, and 2.7 (95% CI 2.3–3.4) months for higher dNLR (P < 0.0001). **Multivariate:** ECOG PS > 0, brain and liver metastasis and raised dNLR associated with worse OS. Baseline dNLR ≥ 3 remained associated with double-increased risks of death (HR=2.29; 95% CI 1.86–2.82; *P*<0.0001)	Neutrophilia and elevated dNLR are associated with an OS and disease progression. Simultaneous elevation of ANC and dNLR was associated with a 5.7-fold increased risk of death and a 4-fold increase risk of progression.
Ferruci et. al, 2015	NLR, WBC and ANC were associated with response (*P*=0.0002, *P*=0.0006, *P*<0.0001 respectively). ALC was not associated with response.	**Univariate**: LDH (*P*=0.03), ECOG PS 0–1 (*P*=0.0001) and NLR<5 (*P*<0.0001) associated with improved PFS. WBC and ANC (*P*<0.0001 and *P*=0.0004) associated with PFS. **Multivariate:** NLR was associated with disease progression (HR=0.38; 95% CI: 0.22–0.66; *P*=0.0006)	**Univariate:** prolonged OS associated with ECOG PS 0–1 (*P*<0.0001), NLR<5 (*P*<0.0001), WBC<UNL (*P*<0.0001), ANC<ULN (*P*<0.0001) and ALC>LLN (*P*=0.04). **Multivariate:** NLR was associated with OS (HR1⁄40.24; 95% CI: 0.13–0.46; *P*<0.0001),	Patients with baseline NLR< 5 had a significantly improved PFS and OS in a multivariate analysis. Associations of low NLR were confirmed in three validation cohorts.

Paper	Response	PFS	OS	Interpretation/ conclusion
Ferruci et. al, 2017	N/A	**Univariate:** LDH-ratio ≤ 2.5, absence of visceral metastasis and RLC ≥ 17.5% associated with delayed progression. REC ≥ 1.5% was not significant in **multivariate analysis** (HR = 0.73; 95% CI: 0.48–1.10).	**Univariate:** LDH- ratio ≤ 2.5, absence of visceral metastasis and RLC ≥ 17.5% associated with improved OS. **Multivariate:** RLC ≥ 17.5% had a reduced risk of mortality (HR 0.31; 95% CI: 0.19–0.51) + REC ≥ 1.5% had an improved OS compared to REC < 1.5% if they received anti-CTLA-4 (HR=0.56; 95% CI 0.34–0.93)	Baseline REC is a potential predictive biomarker for OS in both uni- and multivariate analyses
Flem-Karlsen et. al, 2020	N/A	Mean sAXL expression increased from 26.6 ng/mL (95% CI = 24.3–28.9 ng/mL) in patients at lymph node resection to 54.1 ng/mL (95% CI = 50.7–57.6 ng/mL) in patients at the start of ipi treatment (*P* < 0.0001)	In patients at week 7 of ipilimumab, sAXL levels were increased in patients who died within 2 years (71 ng/mL, 95% CI 61.4–81.2 ng/mL) compared to those still alive (58.1, 95% CI 51.8 ng/mL-64.4 ng/mL) (*P* = 0.03).	High sAXL levels were linked to a higher number of metastases and lower survival at week 7 of treatment. Plus, higher sAXL levels were observed in late-stage melanoma patients compared to patients at an earlier stage.
Forschner et. al, 2019	**Univariate**: Tumor mutational burden (*P*=0.002) detectable ctDNA (*P*=0.0011) and increasing ctDNA (*P*=0.008) were associated with response.	N/A	**Univariate**: 50% increasing cell-free DNA, detectable or increasing ctDNA at first follow-up were negatively associated with OS (*P*=0.006,*P*=0.03). High TMB showed a trend towards prolonged OS (*P*=0.06)	Response and OS could be associated with high TMB, >50% decreasing cell-free DNA or undetectable ctDNA at first follow-up.
Frankel et. al, Neoplasia a 2017	**Microbiomes: r**esponder microbiomes were enriched with B. caccae (*P* = .032) and Streptococcus parasanguinis (*P* = .048). Among IN patients, responder microbiomes were enriched with prausnitzii (*P* = .032) and Holdemania filiformis (*P*=.043) and tBacteroidetes Bacteroides thetaiotamicron (*P* = .046). Among PEM, responders were enriched with Dorea formicigenerans (P = .045). **Metabolics:** 3 metabolites were different when comparing the responder group to progressive group (49 increased; 34 decreased; *P* = .05,	N/A	N/A	Specific gut microbiota species and numerous gut metabolites were associated with response to ICT therapy, however no multivariate analysis was performed.
Fröhlich et. al, eBioMedicine 2020	No significance for mean methylation levels in samples from non-responding tumors (62.7% methylation) in comparison to samples from responding (53.4% methylation) tumors (*P*=0.17). n mRNA levels were not associated with response either (*P*=0.025)	Continuous log2-transformed mRNA levels associated with PFS (HR 0.92, 95% CI: 0.850.99, *P*= 0.022). Methylation of CpG site targeted by bead 12 correlated with PFS (HR = 8.34, 95% CI: 1.24-56.1, *P* = 0.029). Elevated TNFRSF9 mRNA expression + TNFRSF9 hypomethylation correlated with superior OS. In patients receiving anti-PD-1), TNFRSF9 hypermethylation and reduced mRNA expression correlated with poor PFS.	OS analyses were not performed in treated melanoma patients.	TNFRSF9 mRNA expression is correlated with OS and PFS in a univariate analysis. TNFRSF9 Methylation of CpG site targeted by bead 12 correlated with PFS but not response after anti PD-1 therapy in univariate analysis
Fujimura et. al, 2018	sCD163 levels at day 42 were increased in group showing objective response (*P*<0.0001) in cutaneous melanoma, whereas no difference in sCD163 levels was seen among mucosal melanoma	N/A	N/A	sCD163 was increased after 6 weeks in responders after start of nivo for cutaneous melanoma. No differences between responders and non-responders was seen among non-cutaneous melanoma.
Fujimura et. al, 2019	**Univariate:** High baseline CXCL5 correlated with objective response (*P*=0.016). No relationships between serum CXCL10 and CCL22 and objective response (CXCL10: *P*= 0.674, CCL22: *P*= 0.360).	N/A	N/A	Baseline serum levels of CXCL5 were higher in responders than in non-responders. No significant differences in baseline CXCL10 and CCL22 between responders and non-responders.
Galore-Haskel et. al, 2015	**T-test**: Expression of hsa-miR222 of no benefit patients was 2.3-x higher (*p*=0.001) than in clinical benefit patients. **Validation**: high miR222 expression remained associated (CB *N*=7 and NB *N*=15)	N/A	N/A	In this small cohort, expression of miR-222 in melanoma tissue specimens was associated with response for ipilimumab.
Gambichler et. al, 2018	**Univariate:** elevated S100B levels (*P*=0.0049), increased LDH levels (*P*=0.015), positive BRAF status (*P* = 0.049), high CRP levels (*P* =0.025) associated with worse ORR. PD-1 Ab before	**Univariate:** prolonged PFS associated with low-LDH levels (*P* = 0.019), BRAF mutation (*P* = 0.009) and >2 ipilimumab cycles (*P* = 0.043). **Multivariate:** low-LDH	N/A	In multivariate analyses, low serum LDH before ipilimumab treatment was a predictor for improved PFS, low serum S100B is an

Paper	Response	PFS	OS	Interpretation/ conclusion
	ipi resulted in improved ORR (P=0.015). **Multivariate**: PD-1 predictive for ORR (*P*=0.028, OR 7.7 (95% CI:1.3–47.2).	levels and >2 ipilimumab cycles turned out to be a predictor for prolonged PFS (*P* = 0.0041 and *P* < 0.0001, respectively)		independent predictor for MSS and the number of ipilimumab cycles (>2) with prolonged PFS.
Gao et. al, Cell, 2016	Non-responders had more somatic mutations including CNAs and SNVs of IFN-γ pathway genes: 184 mutations detected in 12 non-responders (142 CNAs and 42 SNVs); 4 mutations in responders (SNVs). **Permutation test**: enrichment of mutations in non-responders not observed (*P*=0.015). CNAs of IFNg pathway associated with lack of response in DC (response 4/16) *P*=0.019 and VC (18/88) *P*= 0.035.	N/A	Survival data not for melanoma patients receiving ICIs	Copy number alterations of the IFNg pathway associated genes are associated wit response to ipilimumab.
Gebhardt et. al, 2015	**Univariate:** Increase in AEC (*P* < 0.0001), low ANC (*P*=0.003), low AMC (*P* < 0.05), a low WBC (p=0.0487) associated with response. **Multivariate:** AEC between baseline and point 1 predicts response (*P*=0.017; OR of 23.2) + moMDSC levels in nonresponders higher (*P* < 0.05;) + moMDSCs in responders reduced after first infusion. In nonresponders, elevation upon second Ipi infusion (*P* < 0.05, *P*<0.01) + intracellular NO elevated in moMDSCs from nonresponders (*P* < 0.05) + S100A8/A9 and HMGB1 nonresponses after first Ipi infusion (*P*< 0.05)	N/A	N/A	Higher neutrophil and monocyte count at baseline and early elevation of moMDSC frequencies and serum levels of S100A8/A9 and HMGB1 indicated a lack of response. Early increase in eosinophil counts and reduction in moMDSCs, S100A8/A9, and HMGB1 were associated with response.
Ghorani et. al, 2018	N/A	N/A	Neither neoantigen DIA nor mutational burden correlated with OS in the melanoma cohorts	Neoantigen DIA and mutational burden wer not associated with OS in patients with melanoma
Gide et. al, 2019	**Selection of most relevant outcomes: Monotherapy:** High expression of IFN-related genes, including TBX21, TBET, STAT1, IRF1, TNF, IFNG, and T cell genes (CD8A, CD8B, ITGAE CD103, PDCD1 PD-1, CCL5, CXCL13, IL2), associated with better outcomes. Responding patients expressed higher proteins in tumors, including TIGIT, TNFRSF9 (CD137), IDO, and LAG3. **Combi:** T cell-related genes (CD8A, CD247, CD5, CD6, and CD69) and genes for NK cell-mediated cytotoxicity (CD96) T cell cytotoxicity (GZMK, CD274 [PD-L1], CD2 and ITGAL) cytokine signaling (CXCL13, CCL4, CCR5, CCL5, CXCL9) and TIGIT/CD226 axis upregulated in responders + Nonresponders showed upregulation of IX (CA9) (log2 fold change = 1.21, adjusted *P*=0.0026) + In EDT biopsies, intratumoral PD-1 and PD-L1 densities higher in responders to monotherapy + combined immunotherapy. Intratumoral CD8 associated with ICIs at EDT in combined ICIs.	**Selection of most relevant outcomes:** PFS was longer for monotherapy-treated patients with high expression of the CD8+/CD4+EOMES+CD69+CD45RO+ memory T cell phenotype compared with those with low expression (24 vs 3 months for both CD8 and CD4, *P*=0.027). While a longer PFS (19 vs 6 months) was observed in combined immunotherapy-treated patients with high expression, the results did not reach significance.	N/A	T-cell immune profiles of infiltrating tumou cells and underlying gene profiles, could predict response to anti-PD-1-based therapies. Mainly, higher gene expression profile of EOMIES+CD69+CD45RO+ T cells was associated with greater tumor shrinkage in anti-PD and combination treatment
Goltz et. al, 2018	mCTLA4 expression was higher in patients with progressive disease (*P*=0.050)	N/A	**Cox regression:** mCTLA4 expression was predictive for OS (*P*<0.001 and HR 2.06, 95% C.I. 1.29-3.29).	mCTLA4 expression is predictive for diseas progression and OS in ipilimumab + nivolumab.
Gopalakr ishnan et al, 2017	Rare low abundance OTUs associated with response, with enrichment of Clostridiales in R and Bacteroidales in NR in gut microbiome (*P*<0.01). No association for oral microbiome. **Univariate**: alpha diversity [HR 3.60, 95% C.I.1.02-12.74] abundance of Faecalibacterium (HR 2.92, 95% C.I.=1.08-7.89) and Bacteroidales (HR 0.39, 95% CI 0.15-1.03) in fecal microbiome associated with response.	Patients with high Faecalibacterium abundance had a significantly prolonged PFS versus those with a low abundance (*P*=0.03). Patients with a high abundance of Bacteroidales had a shortened PFS compared to those with a low abundance (*P*=0.05).	N/A	gut microbiome (e.g. high diversity and abundance of Faecalibacterium/ Bacteroidales) were associated with respons and PFS in multivariate analyses. Oral microbiomes were not associated with response or PFS.

Paper	Response	PFS	OS	Interpretation/ conclusion
Graves et. al, 2019	PD-1 on CD4+ cells higher in non-responders (*P*=0.009) but not on CD8+ T-cells. TIM-3 higher on both CD4+ and CD8+ T cells in responders (*P*=0.017 and p=0.023). LAG-3 not associated with response.	N/A	N/A	PD-1 expression on T-cells and TIM-3 on both CD4+ and CD8+ T cells were associated with response to pembrolizumab.
Hamid et. al, 2011	Baseline TIL scores not associated with clinical activity. Association observed between baseline change in TILs and response (*P*= .005). Associations between clinical activity and pretreatment FoxP3 and IDO expression (*P*= 0.014 and 0.012). No associations between clinical activity and total infiltrate; CD4, CD45RO, CD8, granzyme B or perforin; or amount of normal tissue, viable tumor, necrotic tumor, fibrotic regression or peritumoral immune cells. Genetic polymorphisms did not predict clinical activity. No association between HLA allele or allele *0201 at locus HLA-A and clinical activity.	N/A	N/A	Baseline expression of immune-related tumor biomarkers FoxP3 and IDO expression and a post-treatment increase in TILs may be positively associated with ipilimumab clinical activity. 40 biomarkers tested in 82 patients.
Hamid et. al, 2019	Association between GEP and response to pembro in treatment-naive (AUC 0.74, 95% CI 0.61-0.87 *P*=0.0013) and treatment-exposed patients (AUC 0.65, 95% CI 0.58-0.73 *P*=0.0002) and in ipi-naive (AUC 0.7, 95% CI 0.61-0.79 *P*=0.0001) and ipi-exposed patients (AUC 0.66, 95% CI 0.56-0.76 *P*=0.003).	Estimated 5-year PFS rates were 21% in all patients and 29% in treatment-naive patients; median PFS was 8.3 months (95% CI, 5.8–11.1) and 16.9 months (95% CI, 9.3–35.5), respectively	Estimated 5-year OS was 34% in all patients and 41% in treatment-naive patients; median OS was 23.8 months (95% CI, 20.2–30.4) and 38.6 months (95% CI, 27.2–not reached)	GEP score was associated with overall response to pembrolizumab in treatment-naive and exposed patients and in ipilimumab-naive and exposed patients
Heidelberger et. al, 2017	70% of responders were PS 0 vs. 36% of non-responders (*P* = 0.04).	**Univariate**: PFS associated with normal LDH levels (5.2 vs. 2.6 months in elevated LDH, *P*=0.05), wild-type BRAF (4.9 vs. 2.3 months in mutated BRAF, *P*=0.02) **Multivariate**: PS 0[1.19–4.8]), normal LDH levels (p=0.016, HR 2.33 [1.17–4.6]) wild-type BRAF (p=0.02, HR 2.38 [1.11–5.1]) associated with longer PFS	N/A	Normal LDH levels and wild-type BRAF status were predictors of PFS in a multivariate analysis.
Hogan et. al, 2019	**anti–CTLA cohort**: DE50 is significant in the **univariate** model, and only independent factor in the **multivariate** model (HR 2.4 95% CI 0.191 - 7.29 *P*=0.03). **anti–PD1 cohort**, **univariate**: LDH and DE50 correlated with response. **Multivariate**: DE50 is a predictive factor (HR 3.66 95% CI 1.318 - 8.581 *P* = 0.001) Baseline DE50 level below 20.4% associated with good response at 12 weeks (*P*= 0.0016)	**Univariate:** S100 (b-coeff=.814, 95% CI 3.027 to 0.601, *P*=0.003) and LDH (b-coeff=0.945, 95% CI 1.761 to 0.129, *P*=0.023) correlated with PFS. **Multivariate:** DE50, ANC/ALC, ALC, and S100 were associated with PFS	N/A	Low DE50 values were predictive of a longer PFS and good responses to PD-1 blockade, but, on the other hand, predicted a poor response to CTLA4 inhibition.
Hong et. al, USA 2018	N/A	Decreased CTC score between pretreatment and 6–7 weeks of treatment had improved PFS, compared with patients who had increased CTC (HR), 0.17; 95% CI, 0.05–0.62; *P*= 0.008	There was an association between the on-treatment reduction in CTC score and improved OS (multivariable HR, 0.12; 95% CI, 0.02–0.91; *P* = 0.04)	A decrease in CTC score within 7 weeks of therapy correlates with progression-free survival and overall survival, although it wa not clear how the results were validated in this study.
Huang et. al, 2017	A Ki67+CD8+ T-cells to TMB ratio > 1.94 at 6 weeks associated with better outcome by ORR (*P*=0.004). This was confirmed in the second independent cohort.	Patients with longer PFS had low TMB. A Ki67+CD8+ to TMB ratio greater than 1.94 at 6 weeks associated with better PFS (*P*=0.03)	Ki67 to tumour burden ratio of 1.94 was associated with overall survival by 6 weeks after treatment. (*P*=0.06)	The magnitude of reinvigoration of circulating T(exhausted) cells (Ki67) (in relation to pretreatment TMB) correlated with response
Hugo et. al, 2016	BRCA2 mutations were more frequent in responders (OR 6,2 *P*=0,002). Between responders and non-responders, no difference in non-synonymous single nucleotide variants, HLA class I or II neoepitope load, tetrapeptide signatures, TMB, other specific mutations	N/A	Higher mutational load was associated with OS (*P*=0.005)	High mutational load correlates with OS, bu not with response. No multivariable analysis or correction for known prognostic factors. Too few events to study many variables. Participants not specified.
Iivanainen et. al, 2019	N/A	Median PFS was months shorter in patients with CRP > 10 (17 vs 3 months, *P*=0.03). NLR <2.65 associated with better PFS (7.0 vs 2.0 months *P*=0.02). Both outcomes were confirmed in the validation cohort.	**Univariate**: Median OS was different between patients with CRP ≤10 and CRP >10 (*P*=0.0001). **Validation:** there was a statistically significant difference in OS (*P*=0.002) according to CRP. NLR was associated with OS (*P*=0.009).	CRP and NLR associated with PFS and OS in anti-PD-1 in a development + validation cohort. This cohort included melanoma and other tumour types. Not clear whether these

Paper	Response	PFS	OS	Interpretation/ conclusion
				outcomes were in uni- or multivariate analyses
Inoune et al, 2016	PD-L1 PD-L2, Granzyme A, decreased TCR repertoire and HLA-A higher in pre-treatment tumors of responders (n=5) than non-responders (*N*=8) (*P* = 0.03, *P*= 0.04, *P*=0.01, *P*=0.006). CD4, FOXP3, IL-10, TBX21 and GATA3 not significant.			In a small group of patients, TCR repertoire, CD4/CD8 ratio, HLA-A and Granzyme A were associated with response. Response no defined, no confounder adjustment.
Indini et. al, 2019	N/A	Median PFS was 16.4 and 4.5 months for patients with <3 and ⩾3 metastatic sites (*P*= 0.001); 16.0 and 2.68 months for LDH ⩽ and > ULN, (*P*< 0.001); 3.7 and 15.8 months RLC <17.5% and >17.5%, (*P* = 0.004). Changes in monocyte and eosinophil counts had no impact on PFS	Median OS was 21.9 and 10.8 months for patients with <3 and ⩾3 metastatic sites, (*P* = 0.014) ; 28.9 and 4.1 months for low/high LDH > ULN, (p < 0.001); and 5.0 and 33.6 months for patients with RLC <17.5% and >17.5% (*P*<0.001). AMC + AEC did not impact on OS	Metastases burden, LDH levels, and RLC are factors associated with outcome in patients with melanoma receiving anti-PD-1
Jacquelot et. al, 2017	**Univariate:** Regarding clinical response at 12 weeks, CD95 on blood CD4+ T cells associated with OS (*P* = 0.023). **Multivariate;** neither CD95 nor PD-L1 on CD4+ and CD8+ T cells observed to be significant. **Combi therapy:** Detectable expression CD137 on blood+ tumor CD8+ T lymphocytes (and to a lesser extent CD4+ T cells) correlated to response (*P*=0.04)	**Univariate:** the highest prognostic clinical covariates on PFS was history of protein kinase inhibition (PKI). **Multivariate:** PD-L1 expression on circulating CD4+ T cells and to a lesser extent on CD8+ T cells were prognostic on PFS after CTLA-4 blockade: *P* = 0.009 for PD-L1+CD4+ and p = 0.056 for PD-L1+CD8+.	**Univariate:** LDH and previous chemotherapy/radiotherapy or PKI were associated with OS. **Multivariate:** PD-L1+CD8+ T cells was observed to be prognostic on OS (*P*= 0.011).	PD-L1 expression on peripheral T cells was prognostic on OS and PFS. Detectable CD137 on circulating CD8+ T cells was associated with response.
Jensen et. al, 2018	**Continuous:** PRO-C3 (*P*=0.011), C1M (p=0.003), C3M (*P*=0.013) and C4M levels (*P*=0.027) at baseline were elevated in patients with progressive disease. **Dichotomous:** High levels (Q4) of PRO-C3, C1M, C3M and C4M resulted in ORs for being in the PD group of 6 (95% CI = 1.2–28, *P*= 0.019), 14 (95% CI = 2.2–151.6, *P* = 0.003), 6 (95% CI = 1.2–28, *P* = 0.019) and 14 (95%CI = 2.2–151.6, *P* = 0.003), respectively.	High C1M levels were predictive of PFS by the univariate analysis (HR = 2.13, 95%CI = 1.17– 3.88, *P* = 0.013) and borderline significant with the multivariate analysis (HR = 1.84, 95%CI = 0.97–3.51, *P* = 0.064)	**Dichotomous;** high pre-treatment PRO-C3 (HR 2.13, 95%CI = 1.12–4.04, *P*=0.021) and C4M (HR 2.43, 95%CI 1.26–4.70, *P*=0.008) predictive of poor OS. High VICM (Q3 + Q4) at baseline predictive of survival benefit (HR 0.54, 95%CI 0.29–0.99, *P*= 0.044). High LDH (> 250 IU/L) associated with poor OS (HR 2.02, 95%CI 0.99–4.12, *P*=0.052). **Multivariate:** high PRO-C3 and C4M (Q4) predicting poor OS (HR 2.04, 95%CI 1.00–4.16, *P*=0.049 and HR 2.18, 95%CI 1.01–4.70, *P*=0.046). High VICM (Q3 + Q4) predictive of survival benefit (HR 0.49, 95% CI = 0.26–0.92, *P*=0.026) + High C3M/PRO-C3 ratio at baseline (Q2 + Q3 + Q4) associated with longer OS (HR 0.47, 95%CI 0.24–0.95, *P*= 0.034).	High baseline levels (Q4) of the collagen biomarkers PRO-C3, C1M, C3M and C4M were associated with poor response to IPI. In addition, high PRO-C3 and C4M (Q4) were associated with shorter OS.
Ji et. al, 2012	**Summary:** 193 genes associated with response, mostly immune-related to T-cell surface markers such as CD8A, CD2, CD247, CD27, CD38, and CD3, and members of TNF receptor family; cytokines and chemokines (CXCL9, CXCL10, CXCL11, CCL4, and CCL5,) immune-receptors(IL10RA, IL1RB2, IL15RA, IL21R, CXCR6, and CCR5) cytotoxic factors (perforin 1and various granzymes) and various types of T cell receptors, MHC molecules and immunoglobulin genes.	N/A	There was an apparent survival advantage in patients with higher post-treatment expression of the potential biomarkers. (CXCL11 and CXCR3)	There was an apparent survival advantage in patients with higher post-treatment expression of the potential biomarkers.
Jiang et. al, 2018	N/A	**Multivariate:** High SERPINB9 level correlates with short PFS after ICB treatment (coeff 0.437, SD 0.167, Z =2.618, *P*=8.850E-03)	**Multivariate:** High SERPINB9 level correlates with short OS (coeff 00.446, SD=0.175, Z=2.551, *P*=1.073E-02). TIDE prediction score is associated with survival after anti PD-1 Ab (*P*=0.011) and anti CTLA-4 Ab (*P*=0.001) therapy.	TIDE prediction score is associated with survival after anti PD-1 Ab and anti CTLA-4 Ab therapy. SERPINB9 expression is associated with worse overall survival and PFS.
Johnson et. al, 2018	PD-1/PD-L1 interaction score associated with response (DC *P* =0,02; VC *P* =0.06). IDO-1/HLA-DR score is associated with response (DC *P* = 0.0006; VC *P*=0.0002). A biomarker signature composed of two scores associated with response: One/ both scores positive vs both negative (DC *P*=0.0005, VC *P*=0.0096). Individual biomarkers (PD-1, PD-L1, IDO-1, HLA-DR) not associated with response.	**Development:** patients with PD-1/PD-L1 and/or IDO-1/HLA- DR experienced improvement in PFS (HR=0.36; *P*=0.0004) and OS (HR =0.39; *P* = 0.0011) **Validation cohort:** patients with high PD-1/PD-L1 and/or IDO-1/HLA-DR experienced improved PFS (HR = 0.36 95% CI 0,2-0,65; *P* = 0.0004). PD-L not associated with PFS	**Development:** patients with PD-1/PD-L1 and/or IDO-1/HLA- DR experienced improvement in OS (HR=0.39; *P*=0.0011) **Validation:** patients with high PD-1/PD-L1 and/or IDO-1/HLA-DR experienced improved OS (HR = 0.39 95% CI 0,21-0,70 ; *P* = 0.0011). PD-L1 expression not predictive of survival. **Multivariate**: OS not confounded by metastatic stage or LDH levels.	PD-1/PD-L1 interaction and IDO-1/HLA-DR score are associated with response to and PFS and OS after anti-PD-1 Ab therapy.

Paper	Response	PFS	OS	Interpretation/ conclusion
Johnson et. al, 2015	**Univariable:** MHC Class II expression on tumour associated with response. Cohort 1; 79% of patients with positive MHC class II staining (>5%) had CR or PR vs 38% with negative MHC class II staining. *P*=0.033, Cohort 2 p= 0.025. HLA-A, CD4+, CD8+ Tcells, PD-L1 not associated with response.	Development and validation cohorts together: PFS was associated with MHC Class II expression: median not reached for those with positive expression versus 3,2 months for those with negative expression *P*= 0.02)	Development and validation cohorts together: OS was associated with MHC Class II expression: median not reached for those with positive expression versus 27,5 months for those with negative expression *P*= 0.003)	MHC-II positivity on tumour cells is associated with response, PFS and OS after antiPD-1Ab therapy. HLA-A (MHC-Class I), CD4+ or CD8+ T cell infiltration or PD-L1 are not associated with response.
Johnson et. al, 2016	Responders to anti–PD-1/PD-L1 had higher TMB in DC (median 45.6 vs. 3.9 mutations/MB; *P*=0.003) and a VC (37.1 vs. 12.8 mutations/MB; P=0.002) compared with non-responders. TCR clonality did not predict response. ORR was greatest in high (85%) intermediate (29%) and low (14%) TMB groups (*P* < 0.001)	**Univariate: s**uperior PFS was observed in high TMB compared with the intermediate and low groups (median not reached vs. 89 days vs. 86 days, *P* < 0.001; **Multivariate:** High mutation load was also associated with superior PFS (high vs. low HR, 0.14, *P*< 0.001)	**Univariate**: Superior OS was observed in high mutational load group compared with the intermediate and low groups (median not reached vs. 300 days vs. 375 days; *P* < 0.001). **Multivariate:** High TMB associated with superior OS. (high vs. low HR, 0.09, *P*< 0.001).	Tumour mutational load is associated with response, progression-free survival, and overall survival in uni- and multivariate analysis.
Kalaora et. al, 2020	High levels of PSMB8 and 9 had more durable response (*P*<0.006). TMB associated with response *P*<0.03).	N/A	N/A	High PSMB8 and 9 were associated with prolonged response compared to healthy controls.
Karachaliou et. al, 2018	19 patients evaluable for response. Disease control rate was 71.43% for those with high IFNG expression compared with 20% for patients with low IFNG expression (1 out of 5 patients had stable disease),	Univariate analyses: median progression-free survival was 1.9 months (95% CI 0.0–5.1) and 5.0 (95% CI 1.5–14.1) months for patients with low and high IFNG mRNA, respectively (*P*= 0.0099), (HR for disease progression, 3.77; 95% CI 1.23–11.16; *P* = 0.0164). In Multivariate analyses: no significant effect	Univariate analyses: Median OS 3.1 months (95% CI 0.0–11.8) for patients with low IFNG mRNA while it was not reached (95% CI 2.6–NR) for patients with high IFNG mRNA expression (*P*= 0.0183), (HR for death, 3.50; 95% CI 1.16–10.60; *P* = 0.0265). Multivariate analyses: ns effect	IFNG was associated with response and survival, but this effect disappeared after adjustments for relevant confounders
Kasanen et. al, 2020	**Baseline:** CXCR increased in the responders' CD8−CD3+ T cells. Responders had lower NKp30-expressing CD3bright NKT cells (d =−1.70, *P*=0.02), NKG2C and CD25-expressing CD8+ T cells (d =− 0.66, *P*=0.02, d =− 1.24, *P* =0.02). **During treatment:** ALC higher in responders (Rmean: 1.9 109/L vs. NRmean: 1.2 109/L, P = 0.04) after 3 months. Responders have high PB NKT cells, mean total NKT cells (R: pre 11.9% vs. 1mo 13.2%, *P* = 0.01) and CD3bright NKT cells (R: pre 2.4% vs 1mo 3.3%, *P* = 0.007) increased in the responders, but not in the nonresponders. CD25 and CD45RO on responders' CD56dim NK cells increased after 1 month (CD25: pre 23.0% vs.1mo 28.6%, p=0.03, CD45RO: pre 21.1% vs. 1mo 27.6%, *P*=0.04). Chemokine ligands increased in responders after 1 month (NPX CXCL9: pre 430 vs.1mo 1010, p = 0.001, CXCL10: pre 1300 vs.1mo 2600, p = 0.03, CXCL11: pre 50 vs. 1mo 78, *P* = 0.03). After 3 months, responders had higher IL-12B (42 vs. 20, *P* = 0.02), MCP-4, OPG, IL-10RB, IL-15RA, HGF [:d =0.52, *P* =0.01; d =0.42, *P* =0.02; d =0.27,*P*=0.02; d=0.11, p=0.03; d=0.35, p=0.03), and higher proportion of PB PD1-expressing CD56bright NK cells (d = 0.55, p = 0.03) and naïve CD8+ T cells (d=−0.44,p=0.03)	Age 70 years or older was predictive for PFS (HR 0.36, 95% C.I. 0.1-1.3, *P*=0.03). Lower proportion of PB-naive T cells before therapy was associated with longer PFS (HR 0.36 95% C.I. 0.1-1.2, *P*=0.04). High pretreatment levels of serum OPG and MCP-4 were associated with longer PFS (HR 0.28, 95% CI 0.1-1.0, *P*=0.008). Negative predictors were not reported	Not available	In this small and selected cohort, baseline CXCR3 in CD8−CD3+ T cells, NKp30-expressing CD3bright NKT cells, NKG2C and CD25-expressing CD8+ T cells are associated with response.<br>During treatment ALC, NK, CD25 + CD45RO on NK cells and CXC levels were increased in responders. High age was predictive for PFS.
Kelderman et. al, 2014	RR in patients with LDH above normal was 9 % compared to 23 % in the ≤1× Uln group (ns).	NL median PFS 2.9 months (95 % CI 2.8–3.2).	Baseline ALC and week 6 ALC s showed difference in OS when stratifying high vs low (*P* = 0.01 and *P* = 0.001). Value> 1.35-fold compared with baseline after two cycles had higher OS than lower increase (*P* = 0.02;). low baseline LDH, S100 and ESR associated with improved OS (all *P* < 0.0001**)**. **Multivariate:** ESR and LDH negative correlation (*P* < 0.01). **Validation:** stratification on 1×ULN baseline LDH did not influence OS. Difference in OS when stratifying patients by 2×ULN LDH. Median OS in LDH-high group was 3.2 months, 5.0 months in LDH-low group (*P* = 0.004).	Baseline LDG, ESR, S100 and ALC and ALC at 6 weeks are univariable associated with OS. In a multivariable model, only ESR and LDH remained associated in development cohort. In a validation cohort, LDH > 2x maximum normal value were associated with worse survival.

Paper	Response	PFS	OS	Interpretation/ conclusion
Keller et. al, 2019	ctDNA-decrease from baseline to week 2-6 detected all responders + early ctDNA increase detected 5/6 responders. Association between early ctDNA and tumour response at first evaluation (*P*=0.0046).	The median PFS was 21 days for patients with increased ctDNA vs. 145 days for patients with decreased ctDNA. PFS was significantly better for patients with decreased ctDNA (*P*= 0.001)	N/A	Changes in ctDNA quantities in the first weeks of immunotherapy may be associated with response or PFS in univariate analyses.
Khattak et. al, 2020	**Univariate**: patients with PD-L1+ CTCs were more likely to be responders compared with patients with undetectable PD-L1+ CTCs (OR, 8.67; 95% CI, 1.19–342.96; *P* = .017;	Patients with detectable PD-L1+ CTCs (14/25, 64%) had longer PFS compared with patients with PD-L1 CTCs (26.6 vs.5.5 months; p = .018). **Univariate:** D-L1+ CTCs had longer PFS compared with patients with PD-L1− CTCs (HR 0.20, 95% CI 0.05–0.77, *P*=0.02) **Multivariate:** CTC PD-L1 positivity was associated with PFS (HR 0.11; 95% CI, 0.01–0.81; *P* = .03;	N/A	PD-L1 expression on CTCs may be associated with response to pembrolizumab and longer PFS.
Khoja et. al, 2016	**Univariate:** LDH at all time points associate with response; SD, PR, CR (baseline LDH P=0.0003, post cycle 2 *P*=0.027, end of treatment *P* = 0.001), as was change in LDH post cycle 2 to end treatment (*P* = 0.023). PLR at all time points significant (baseline PLR *P* = 0.023, post C2 *P* = 0.034, and end of treatment *P* = 0.003). NLR at end of treatment was significant for response *(P* = 0.019). ELR not associated with response.	**Univariate:** LDH at all time points (p<0.0001), NLR (p 0,02), and PLR at baseline (p<0.0001) and at the end of treatment (p 0.0008) and change in LDH during treatment (p<0.0001) were associated with PFS. Median PFS was 2.8 months (95% CI: 2.8–3.2).	**Univariate:** LDH at all time points (p<0.001), NLR (p 0,003), and PLR at baseline (p<0.001) and at the end of treatment (p 0.01) and change in LDH during treatment(p<0.001) were associated with OS. Median OS was 9.6 months (95% CI: 7.9–13.2). **Multivariate:** Baseline LDH (HR = 1.00 95% CI: 1.00–1.00, P < 0.0001), and baseline NLR (HR = 1.04 95% CI: 1.00–1.07, P = 0.04).	Baseline LDH and NLR are associated with OS in uni- and multivariate analysis. PLR, LDH and NLR were associated with PFS and response in univariate analysis.
Kirchberger et. al, 2018	For all treatment categories, ORR was similar for NRAS mutant and NRAS wildtype	The median PFS was not associated with NRAS genotype.	**Kaplan Meier:** Decreased OS for NRAS mutant vs NRAS wild type: median 21 vs 33 months (*P*=0.034). Ipi: NRAS-mutants had median OS of 12 months, NRAS wildtype a median OS of 27 months (*P*=0.046).	Immune checkpoint inhibition shows comparable response rates in NRAS-mutated and NRAS wildtype melanoma even though OS is less favorable for NRAS mutation.
Kondo et. al, 2019	ORR 18,4% (cutaneous 29%, mucosal 10%). **Univariate:** CAR>0.057: OR for early disease progression: (OR 0.11, CI 0.02–0.50). LDH ≥ 227 IU/L and platelets ≥ 25 × 104/μL associated with EPD. **Multivariate:** CAR ≥ 0.057 remained associated with EPD (OR 0.053, 95% CI 0.0057-0.49, *P*=0.010)	A CRP to albumin ratio (CAR) ≥ 0.057 is associated with shorter PFS (1.67 months vs 5.45 months, HR 3.32, 95% CI 1.60–6.90, *P*= 0.001)	Median OS17,1 month. No rates for association with CAR-OS given. Patients in high-risk group (MII = 2) experienced shorter OS than patients in the low- (MII = 0) or intermediate-risk (MII = 1) groups (MII 2 vs MII 1 vs MII 0: 2.9 vs 18.0 vs 27.6 months, HR 21,0 vs 1.76 vs reference, respectively).	A high CRP to albumin ration is associated with early progression of disease during nivolumab treatment.
Krieg et. al, 2018	**Blood**: High expression of HLA-DR, CTLA-4, CD56 and CD45RO, and low CD3, CD27 and CD28 in responders before therapy (no *P*). In responders, CD4+ T cells and CD8+ T cells were lower and CD19−HLA-DR+ myeloid cells higher (adjusted *P*= 1.55 ×10−5, 1.74 x 10−3, and 1.74 ×10−3) (before + after start of treatment). NKT cells higher and lower γδ T cells (adjusted *P* = 3.07 ×10−3 and 2.52 ×10−3) in responders. **Tumor:** responders had higher infiltrating CD4+ and CD8+ T cells. Before and after therapy CD4+T-cells of responders had higher expression of CTLA-4, HLA-DR, CD69,and BTLA.CD8+T-cells had higher CD45Ro, CTLA-4, CD62L, CD69, CD11a and CCR4 in responders before and after therapy + lower circulating CD4+ EM T cells, lower CD8+ naive T cells at baseline and after start treatment (adjusted *P* = 8.21 ×10−3, 6.95 ×10−3). CD8+ T cell subpopulation of responders had higher CM T cells before + after treatment. After start, higher CD4+ T cells expressing PD-1, IL-4, IFN-γ, IL-10, IL-17A and Grz-B in responders. For CD8+ T cells, high CTLA-4 and granzyme B detected in responders. lower T cells (*P* = 1.59 ×10−3) + high CD14+ myeloids in responders (*P*= 5.82 × 10−3)	**Multivariate:** Classical monocytes and immature granulocytes were associated with PF: HR 1.180, 95% CI −2.020 to -0.344, p=0.006 and HR 1.180, 95% CI 0.237 -2.14, *P*=0.014.	N/A	In a small group of patients, many differences were shown between responders and non-responders regarding immune cell subsets and cytokine production at baseline and during treatment with anti-PD-1 antibodies.
Kubo et. al, 2019	Non-responders displayed higher serum concentrations of HGF than the responders (*P* = 0.00124).	**Univariate:** Patients with low HGF showed longer PFS (N = 24, P = 0.0068; HR 0.2087, 95% CI 0.06525–	Patients with low HGF showed longer OS (N = 28, P = 0.039; HR 0.3125, 95% CI 0.1036–0.9427). **Multivariate:**	Hepatocyte growth factor is associated with PFS and OS after anti-PD-1 therapy.

Paper	Response	PFS	OS	Interpretation/ conclusion
		0.6676). **Multivariate:** HGF was associated with PFS (HR 1.02, 95% CI 1.01-1.04, p= 0.0021)	HGF was associated with OS (HR 1.02, 95% CI 1.01-1.04, p= 0.0028)	
Kumpers et. al, 2019	N/A	Trend in increased PFS of patients with diffuse TIL infiltration compared to patients with focally infiltrated TIL's infiltrate (*P* = 0.093)	Increased OS in patients with diffusely infiltrated TIL's compared to focally infiltrated TIL's in primary tumors (p = 0.024). No association between TIL pattern in metastases and OS. Subtyping lymphocytic infiltrate using CD8, CD4, CD56, FoxP3, CTLA-4, PD-1, PD-L1, or PD-L2 not significant for OS and no association between BRAF or of PD-L1 expression on OS or PFS.	Diffuse infiltration as opposed to focal infiltration of TIL's in the primary tumour is associated with improved OS after checkpoint inhibition.
Lee et. al, 2019	ORR and disease control rate (DCR) were 17% (95% CI 11–22%) and 60% (95% CI 52– 68%). NGS: NRASmut, CDKN2A del, CCND1 ampl, MYC ampl, CDK4 ampl, BRAF v600, DCB, DCR and KIT were not associated with response	Median PFS was 4.2 months (95% CI, 1.8–6.6 months). **Univariate:** Low NLR < 2.10, observed in 73 patients (48%), showed superior PFS (median 6.9 vs. 2.4 months,*P* = 0.015). **Multivariate:** NLR was associated to PFS. (HR 1.4, CI 1.16-2.97, *P*=0.009).	Median OS 32.9 months (95% CI, 20.0–45.7). Low NLR (< 2.10), observed in 48%, showed superior OS (median not reached vs. 10.4 months, *P*<0.001). Low LDH associated with superior OS. **Multivariate:** NLR/LDH were significant (NLR HR 4.583 (2.121–9.907), *P*<0.001)	Mutations recovered with NGS (BRAF, KIT, NRAS) are not associated with response or OS/PFS in patients treated with anti PD-1. High NLR was associated with OS and PFS in uni- and multivariate analysis.
Lee et. al, 2017	**Total cohort:** RR was 53%. 72% of patients with undetectable ctDNA before + during therapy had favorable response, 77% with detectable before and during and 6% of patients who had detectable ctDNA before + during therapy had favorable response.	**Between groups:** Median PFS not reached for patients without detectable ctDNA at baseline(grA) or with disappearing ctDNA(grB) but was 2.7 months for patients with persistent ctDNA (grC). [HR 0.09; P<0.001 for group A vs C, and 0.16*;* *P*<0.001 for group B vs C. **Multivariate**: poor outcome measures associated with group C remained significant**.**	**Between groups:** Median OS not reached for patients without detectable ctDNA at baseline (A) or disappearing ctDNA(B), 9,8 months for patients with persistent ctDNA(C) (HR 0.02; P<0.001 for group A vs C and 0.14; *P*<0.001 for group B vs C**). Multivariate:** poor outcome measures associated with group C remained significant	Detectable levels of ctDNA at baseline that remain detectable during therapy are associated with lack of response, and short PFS and OS after anti-CTL-4 Ab therapy in uni- and multivariate analysis.
Li et. al, 2019	N/A	N/A	Compared to all other metabolites, increases Kyn/Trp ratio (week 4 or 6 vs baseline) associated with greater risks for death (p=1.2 ×10−4 ,HR 2.71, 95% CI 1.63–4.51; p = 2.5 ×10−4, HR= 2.26, 95% CI 1.46–3.50. Baseline kyn/trp ratios not associated with OS. **Validation:** at week 4, Kyn/Trp associated with OS in the nivolumab arm (*P* = 4.7 ×10−4, HR 2.81, 95% CI 1.57–5.01; Cox model)	Increase of serum kynurenine/tryptophan ratio during treatment is associated with reduced overall survival in a development and validation cohort.
Liu et. al, 2019	N/A	N/A	**Univariate:** lower CNA levels had longer OS (HR 2.06 (1.34–3.15), *P* < 0.001), but TMB not associated with treatment outcome. **Multivariate:** lower CNA associated with OS (HR 2.06 (1.35–3.15), *P*=0.001). Prolonged OS in TMBhigh-CNAlow compared with TMBlowCNAlow, TMBhighCNAhigh, andTMBlowCNAhigh patients with SKCM treated with anti-CTLA4 (not reached vs. 13.99 vs. 14.6 vs. 10.43 months; *P*= 0.003)	Low CNA is associated with increased OS after antiCTLA-4 therapy in uni- and multivariate analysis. TMB was not associated with treatment outcome.
Luksza et al. 2017	N/A	N/A	Tumour fitness predicts survival (*P* = 0.004, *P*=0.00026)	An immune fitness model based on predicted MHC presentation and recognition by T-Cells of tumour neoantigens predicts OS after anti-CTL-4 therapy
Maccalli et. al, 2017	Baseline ULBP-1 levels are associated with disease control (*P*=0.002)	N/A	**Univariate**: undetectable serum sMICB and sULBP-1 associated with long-term OS (median OS = 21.6, 25.3 months, *P* = 0.02 and 0.01) vs median OS = 8.8 and 12.1 months for patients with detectable levels. **Multivariate:** LDH (*P* <0.0001), sULBP-1 (*P* = 0.02), and sULBP-2 (*P*= 0.02) associated with OS.	Absence of serum sNKG2DLigands ULBP1 and ULBP2 at baseline are associated with response and OS after immunotherapy in uni- and multivariate analysis. LDH is associated with OS.
Madonna et al, 2018	Using a 5% PD-L1 tumor cell expression threshold value to define status, negative PD-L1 expression had 10.2% CR, 2.3% PR, 11.4% SD and 76.1% PD, while PD-L1-positive patients had 9.5% CR, 9.5% PR, 0% SD and 81% PD (*P*= 0.18).		Median OS was 11 months for positive PD-L1 expression and 12 months (*P* = 0.79) for negative PD-L1 expression using a 5% expression cut-off. Using a 1% threshold, median OS for PD-L1 positivity was 12 months, vs 13 months for negative PD-L1 (*P* = 0.56).OS was 17 months	

Paper	Response	PFS	OS	Interpretation/ conclusion
			for BRAF-WT, 11 months for BRAF V600E and 16 months for NRAS-mutant patients (*P*= 0.05). Low CD8+ T cells had longer median OS than higher CD8+ T cells (18 vs 10 months; *P* < 0.04). Above median CD163+ macrophages expressing PD-L1+ had improved OS compared to lower density of PD-L1+ macrophages (16 vs 14 months; *P* <0.05)	
Martens et. al, 2016	N/A	N/A	**Univariate:** Low baseline LDH, AMC, Lin- CD14+HLA-DR-/low-MDSC and high AEC, RLC and CD4+CD25+FoxP3+-Tregs associated with better OS. **Validation:** only routine parameters available: LDH, AMC, AEC and RLC associated with OS. **Multivariate:** LDH, AMC, RLC, Lin- CD14+HLA-DR-/low-MDSC and AEC remained significant.	A baseline signature of low LDH, AMC, and MDSCs as well as high AEC, Tregs, and RLC is associated with favorable outcome following ipilimumab.
Martens et. al, 2016	Increasing frequencies of CD8+ T cells 8 to 14 weeks after starting treatment were observed in patients experiencing clinical benefit, but not significant (*P*=0.005). No other significance found.	N/A	**Cox regression:** Increases in ALC 2- 8 weeks (*P*=0.003) and in % CD4+ andCD8+ T cells 8-14 weeks (*P* =0.001 and *P* = 0.02) after the first dose of ipi correlated with improved OS. Not significant when adjusted for multiple testing	Changes ALC and CD4+ and CD8+ T cells occur during ICIs. No significant association with response or OS where be demonstrated after adjustment for multiple testing.
Mastracci et. al, 2020	**Linear mixed models;** High TIL score, density CD3+, CD8+ T cells and CTLA-4+ immune cells associated with better response to IPI (*P*= 0.002, 0.023, 0.007, and 0.001, for responders vs non-responders. 95% CI 0.08–0.84, 0.10–0.69 and 0.04–0.43.	N/A	N/A	High TIL score and density of CD3+, CD8+ T cells, and CTLA-4+ immune cells in tumor are associated with response to ipilimumab
Matson et. al, 2018	Relative abundances of 10 bacterial species correlated to operational taxonomic units (OTUs) with differential abundance in responders vs non responders (*P*< 0.01).	N/A	N/A	The commensal microbiome may be associated with anti tumour response in patients treated with immunotherapy
Mc Granahan 2016	N/A	N/A	**Clonal architecture analysis**: improved OS in tumors with low neoantigen intratumor heterogeneity (ITH) (= homogeneous tumors) and high clonal neoantigen burden. OS without ITH threshold [HR 0.51 (0.23–1.11), *P* =0.083] or with ITH threshold of 0.01 [HR 0.29 (0.11−0.77), log-rank *P*=0.008], 0.02 [HR 0.34 (0.14−0.81), log-rank *P* = 0.011].Relationship between neoantigen burden and OS not significant without ITH (*P* = 0.083). More homogenous tumor in its neoantigen expression has lower ITH.	Low neoantigen intratumor heterogeneity in combination with high clonal neoantigen burden may be associated with improved survival after antiCTLA4 Ab therapy
Meyer et. al, 2014	Clinical responders to ipi showed less lin− CD14+ HLA-DR− cells compared to non-responders at baseline (*P*<0.05)	N/A	N/A	Lin− CD14+ HLA-DR− cell frequency at baseline associated with response after ipi.
Minowa et. 2018	Baseline NLR was not associated with response.	N/A	**Univariate:** patients with NLR <3.4 had poorer OS (*P*=0.0021)	High NLR is associated with poor OS in patients treated with anti-PD-1 therapy.
Moreira et. al, 2017	N/A	N/A	Median OS 19 months for patients with >5 % eosinophils (n = 59) compared with 10 months for patients with <%5 eosinophils (*N* = 27) p < 0.05; Median OS for patients with >20% eosinophils (*N* = 7) was 35 months, compared with 16 months for patients <20% eosinophils (*N* = 79; *P* = 0.01). Not clear if this is measured at baseline or during therapy.	Eosinophilia during immunotherapy is associated with survival
Morello et. al, 2017	CR, PR and SD, were significantly associated with low pretreatment sCD73 enzyme activity (< 27.8 pmol/min/mg protein) (*P* = 0.001)	Median PFS 2.6 months [95% CI 1.9–3.3] in patients with high sCD73 enzyme activity (> 27.8 pmol/min/mg), and 14.2 months (95% CI 4.6–23.8) in patients with lower CD73 enzyme activity. **Univariate**: low sCD73 associated with PFS (HR 4.24 (1.64–10.93) *P* = 0.003)). **Multivariate:** sCD73 associated with PFS (HR 4.24 (1.64–10.93) *P* = 0.003)).	Median OS not reached in patients with low sCD73 activity (< 27.8 pmol/min/mg protein) compared with 6.1 months (95% CI 0–14.8) in patients with higher sCD73 activity. **Univariate:** low sCD73 was associated with OS (HR 6.27 (2.17–18.11) *P* = 0.001)). **Multivariate:** sCD73 remained associated with OS (HR 6.27 (2.17–18.11) *P* = 0.001)).	The activity of sCD73 in the blood is associated with response, PFS and OS after nivolumab.

Paper	Response	PFS	OS	Interpretation/ conclusion
Morrison et. al, J. 2018	PD-L1 positivity (>=1%) associated with 55.6% ORR, 37. 9% of PD-L1-negative tumors had clinical response. High TMB associated with 33% ORR, 21% of patients with lower TMB achieved response. Response associated with Immune signature (transcriptomics): ORR inflamed tumors (51%), borderline tumors (35%) or immune desert tumors (31%). Tumor genomics not associated with immune signature. No significance levels were revealed for the response results.	N/A	PD-L1 positivity was associated with improved survival (*P*=0.000036). Mutational burden had no association with OS. CD8+ T cell infiltration was associated with OS (*P*<0.018). In the transcriptomic analysis results, the inflamed tumors showed a trend towards improved OS (*P*= 0,063)	PD-L1 positivity and CD8+ T cell infiltration in the tumour was associated wit[illegible] improved survival. Mutational burden had no association with OS.
Muto et. al, 2019	ORR 6,7%	N/A	ALC was not associated with OS in uni- and multivariate analysis. ANC, NLR, LDH, CRP, were not associated with OS	No conclusions can be drawn. No association with biomarkers and outcomes for patients treated with ipi after anti-PD1.
Nakamura et. al, 2016	N/A	N/A	**Univariate:** Elevated LDH and CRP associated with poor OS, HR 0.29 [*P*<0.001], HR 0.42 [*P*=0.004]). **Multivariate:** LDH remains associated. Patients with ALC) ≥ 1000/µl (Week3: HR 0.40 [*P*=0.004], Week6: HR 0.33 [*P*=0.001]) and ANC <4000/µl (Week3: HR 0.46 [*P*=0.014], Week6: HR 0.51 [*P*=0.046]) had better OS.	Baseline LDH is associated with OS after nivolumab treatment in multivariate analysi[illegible] Absolute neutrophil and lymphocyte count i[illegible] the first 6 weeks after start of treatment are associated with OS.
Nakamura et. al, 2019	**Univariate:** no factors associated with response. **Multivariate:** Increased baseline NLR associated with poor response (OR: 2.638, 95%CI 1.145–6.076, *P*= 0.0227, cutoff value = 2.8)	**Univariate:** NLR at baseline and ANC associated with shorter PFS. **Multivariate:** Increased NLR at baseline associated with PFS (HR 1.343, 95%CI 1.067–1.594, *P* = 0.0095)	N/A	In multivariate analysis, increased NLR at baseline was associated with OS and response.
Nie et. al, 2019	ORR was 53.8% (28/52) in the high immunoscore group and 17.7% (14/82) in the low-immunoscore group (*P* < 0.001)	1-year PFS was 42.4% (95% CI 20.6-87.2) in high-immunoscore group and 14.3% (95% CI 4.0- 51.5) in low-immunoscore; PFS difference showed a trend toward significance (HR 0.39, 95% CI 0.14-1.06; *P*= 0.059)	High-immunoscore group had a longer OS trend than did those in the low-immunoscore group (3-year OS: 41.5% [23.7%-72.7%] vs 31.6% [22.9%-43.5%]; HR 0.59, 95% CI 0.34-1.02; *P* = 0.057)	An immunoscore model of RNA gene expression profiles of immune cells can predict anti-PD1 response and the neoadjuvant anti-PD1 response of resectable melanoma, but not PFS or OS.
Nonomura et. al, 2016	Early increase in Th9 cell during treatment associated with improved response (*P*<0.05). Baseline elevated TGF-b associated with response (*P*<0.05). No difference in WBC, ALC, CD8C T cells, CD4C T cells, Tregs, subsets of Th1, Th2, Th17, Th22 cells, LDH levels between responders and nonresponders.	N/A	N/A	In a relatively small number of patients, many immune cells and cytokines were studied and of these, baseline TGF-beta and TL-9 cells in the first weeks after start of nivolumab were associated with response.
Nosrati et. al, 2017	**Univariate:** Elevated LDH is associated with response (OR = 0,38 (0,21-0,69) *P*<0.001). BRAF status and WBC not associated with response. **Multivariate:** elevated LDH remained associated with response (OR=0.48 (0.25, 0.90), *P* = 0.02)	N/A	N/A	Elevated LDH is associated with response i[illegible] uni- and multivariate analyses.
Okuhira et. al, 2018	**Univariate**: Responders showed higher baseline ESR (p=0.006) and CRP(*P*=0.014) and lower ALC level (*P*=0.023). Treatment decreased CRP (*P*=0.012), ESR (*P*=0.018), and ANC (*P*=0.018), while it increased ALC (*P*=0.027) in responders.	N/A	N/A	In a rather small group of patients' univariat[illegible] analysis showed that baseline ESR, CRP an[illegible] ALC are associated with response to nivolumab.
Pan et. al, 2018	**Multivariate:** baseline ANC and platelets associated with progression. HR for ANC ≥ 5501/µL vs ≤ 3900/µL was 2.3 (95% CI = 1.2-4.6, *P* < 0.05). For platelet counts ≥ 304,000 vs ≤ 215,000/µL, HR was 2.0 (CI = 1.0-3.9, *P*< 0.05). For ALC ≥ 1716/µL vs ≤ 1120/µL, the HR was 0.5 (CI = 0.2-1.0, *P*= 0.05)	N/A	N/A	For patients treated with nivolumab or pembrolizumab, higher neutrophil or platele[illegible] counts, or lower lymphocyte counts, are associated with higher risk of progression in multivariate analyses.
Perrone et. al, 2020	N/A	**Univariate:** PFS better in patients with high plasma cholesterol: median PFS was 6.1 vs 2.4 months (*P*= 0.002). **Landmark analyses after 6 months;** PFS was not associated with plasma cholesterol levels	OS was better in patients with high plasma cholesterol levels (>200 mg/dl): the median OS was 19.4 versus 5.5 months (*P*= 0.001). **Multivariate analysis** confirmed this.	In a mixed cancer cohort (15% melanoma), OS after ICI is associated with plasma cholesterol at baseline. Comorbidity and statin intake (27%) are not corrected for.
Pirozyan et. al, 2020	Total and classical CD14+CD16−monocytes higher and subset of NK cells (CD16hiCD56+) smaller in primary resistance group compared with responders. T cell exhaustion markers (CD4 or	N/A	N/A	In a relatively small number of patients many immune cells and cytokines were studies. Only frequencies of AMC, classical

Paper	Response	PFS	OS	Interpretation/ conclusion
	CD8 T cells expressing PD-1, CD39, TIGIT, TIM-3, or KLRG1) do not distinguish between responders and non-responders. No associations between response and subpopulations of CD8 T cells (CCR7 and CD45RO: naïve (CCR7+CD45RO−), central memory (TCM, CCR7+CD45RO+), effector memory (TEM, CCR7−CD45RO+) and terminally differentiated effector memory (TEMRA, CCR7−CD45RO−). No difference in expression of T-bet, Eomes, T-betdim/Eomeshi, or T-bethi/Eomesdim in responders. No differences in granzyme B, IL2, Ki-67, perforin, and TNFα by CD8 and CD4 T cells.			CD14+CD16− monocytes and the major subset of NK cells (CD16hiCD56+) were associated with response after anti-PD-1 antibody therapy.
Pistillo et. al, 2019	Patients with CR or PR more often had baseline sCTLA-4 > 200 mg/ml *(P*=0.02) compared to progressive disease. Response associated with sCTL-4 above 200pg/ml (*P*=0.034) during ICIs	N/A	**Multivariate±** sCTLA-4 > 200 pg/ml had a better prognosis showing a 40% lower risk of death (HR = 0.61, 95%CL = 0.39–0.98, *P*=0.039) compared to sCTLA-4 ≤200 pg/ml	sCTLA-4 levels at baseline are associated with response and OS in patients treated wit ipilimumab
Postow et. al, 2015	Difference in richness (*P* = 0.033) and evenness (*P* = 0.028) of the T-cell repertoire between patients with and without clinical benefit (continuous value).	N/A	There was no association with OS and richness or evenness of the T-cell repertoire	There was no association between richness or evenness of the T-cell repertoire with OS but there was an association with response.
Retseck et. al 2018	N/A	Lower baseline circulating Treg (CD4+CD25hi+ CD39+) associated with better relapse free survival (*P*= 0.04). Circulating monocytic HLA-DR+/loCD14+ MDSC lower at baseline in the relapse-free group(ns).	N/A	Baseline levels of circulating Treg (CD4+CD25hi+CD39+) are associated with relapse free survival (RFS) (p = 0.04).
Riaz et. al, 2016	Mutations in either SERPINB3 or SERPINB4 (SERPINB3/B4) were associated with clinical benefit from anti-CTLA4 therapy. TMB was associated with response (*P*= 0.0047)	N/A	**Multivariate**: SERPINB3/B4 mutations associated with OS in cohort 1 (HR 0.34, 95% CI0.11-0.98*, P*=0.05) and 2 (HR 0.32 95% CI 0.13-0.76, *P*=0.01). No other genes were significant.	Mutations in SERPIN B3/4 associate with response and survival after antiCTLA-4
Riaz et al, 2017	**Pretreatment:** SERPINB3/4 was not associated with response (*p* 0.21). CNA not associated with response nor copy alteration in IFN genes. An immunologically "hot tumor" environment was observed in all Ipi-P patients with CR/PR, immunological activity was observed in Ipi-N patients with CR/PR. Increase in TILs upon Nivo was greater among benefiting Ipi-N but not Ipi-P patients (TCR-seq *P*=0.040; IHC *P*=0.023).No difference in CDR3 (TCR richness) between response status. **Posttreatment:** response was associated with reduction in mutation and NAL in both IPI-N and IPI-P. CDR3 richness associated with benefit in Ipi-P but not Ipi-N (*P* = 0.016, *P*= 0.489;). T-cell evenness associated with benefit in Ipi-N but not Ipi-P (p=0.036,p=0.594).	N/A	Tumor mutation load associated with OS in Ipi-N (*P*= 0.048) but not Ipi-P patients. Ipi-P patients tended to have lower numbers of clonal mutations (*P* = 0.08) and clonal mutations in IPI-N was associated with OS *(P* = 0.003).	In pretreatment samples, SERPINB3/4, CNA or TCR richness were not associated with response to NIVO, but an immune-related active GEP and increase in TILS was associated with response. On-treatment, response was associated with NAL and TCR richness (IPI-P) or TCR evennes (IPI-N). TMB and clonal mutation load was associated with OS in IPI-N patients. No patient characteristics available.
Ribas et al, 2016	Increase in frequency of T cells in responders (*P*= 0.02). On-treatment: B cells (*P*= 0.04) and moMDSCs (*p* 0.04) increased on treatment. Tregs (*P*= 0.54), monocytes (*P*= 0.48), and NK cells (*P*= 0.47) did not change. CD8+ memory T cells expanded in responders (*P*= 0.006). CD4+ effector memory T cells decreased, and CD4+ effector T cells increased in nonresponders.	N/A	N/A	During pembro treatment, responders had more TILs, but no other differences in TIL subsets between responders and non-responders. In a small group of patients, there was a decrease in CD4+ TEM in tumors on pembrolizumab in both responders and nonresponders, and more CD4+ effector T cells only in non-responders.
Rodig et. al, 2018	**MHC class I expression** (≤30%) identified patients more likely to have progressive disease than CR, PR, SD at week 13 after IPI (*P* = 0.02). MHC I expression not associated with response to NIVO. **MHC class II expression** (>1%) associated with patients more likely to have CR/PD/SD progressive disease at week 13 after NIVO (*P* = 0.0517 ns), not associated with response to IPI.	N/A	**MHC class I expression** (≤50%) associated with inferior OS for patients initially treated with 13 weeks IPI, (HR 0.38; 95%CI 0.18-0.82; *P*=0.01), but not with NIVO (HR, 0.70; 95% CI, 0.27 -1.18; *P* = 0.46). OS after treatment in opposite sequence not associated with MHC class I expression. **MHC class II expression** (>1%) associated	Low MHC class I expression on tumour is associated with low response to Ipilimumab, but not to nivolumab or combination therapy. Low MHC class I expression was associated with OS in patients initially treated with ipi. Low MHC class II

Paper	Response	PFS	OS	Interpretation/ conclusion
	**Validation:** association between MHC I expression and response were confirmed (*P*=0.01). MHC I or II expression are not associated with response to combination therapy		with better OS for patients initially treated with NIVO then IPI (NIVO→IPI HR, 0.11; 95% CI, 0.02-0.83). MHC I or II not associated with OS after combination.	expression was associated with OS in initially treated patients with NIVO.
Rosner et. al, 2018	No individual variables correlated with objective response (CR/PR vs. SD/PD).	N/A	**Multivariate;** favorable OS was seen for high relative eosinophils (REC) (*P*=0.007), high relative basophils (RBC) (*P*=0.08), low AMC (*P*=0.01), low LDH (*P*<0.0001) and low NLR (*p*=0.02).	High REC, RBC, low AMC, LDH, and low NLR are associated with OS in uni- and multivariate analyses. No individual variables correlated with objective response.
Sade-Feldman et. al, 2016	Low levels of MDSCs before CTLA-4 therapy correlated with an objective clinical response (*p*<0.008). LDH was not associated with response.	N/A	Low MDSCs before CTLA-4 Ab correlated with OS (inverse correlation (*P* < 0.0022) > 55.5% circulating MDSC's (CD33+CD11b+) of HLA-DR-cells associated with short OS (*P* < 0.003). LDH predicts OS (*p*<0.0001)	Low levels of MDSC's are associated with response and OS after ipilimumab therapy. LDH was only associated with OS.
Sanmamed et. al, 2017	After anti-PD1 Ab, responders' IL-8 levels decrease between baseline and best response (*P* <0.001) and increase upon progression (*P*= 0.004). In non-responders, IL-8 levels increased between baseline and progression (*P*= 0.013). Changes in IL-8(2–4 weeks after treatment initiation) associated with response (*P* <0.001). Validated in 15 pts treated with nivo + ipi (*P* <0.001).	N/A	Early decreases in IL-8 levels associated with longer OS (*P*= 0.001). Early changes in IL-8 levels (2–4 weeks after treatment initiation) were associated with response (*P* <0.001). These observations were validated in 15 patients treated with nivolumab plus ipilimumab (*P* <0.001)	Changes in IL-8 serum levels are associated with response to and survival after checkpoint inhibitor therapy (monotherapy and combination)
Seremet et. al, 2019	**Mann Whitney U**: pretreatment ctDNA in CR/PR-patients was 0 copies/ml of plasma (IQR 0–69) vs 31 copies/ml (IQR 0–647) for SD/PD patients (*P* = 0.0345)	**Univariate:** LDH, CRP, ctDNA, ECOG and number of metastatic sides were associated with PFS. **Multivariate analysis:** Undetectable ctDNA at baseline is associated with better PFS (*P* < 0.001). LDH (*P*<0.001) and CRP(*P*=0.017) are associated with PFS	**Univariate:** LDH, CRP, ctDNA, ECOG and n of metastatic sides associated with OS. **Multivariable analysis:** Undetectable ctDNA at baseline associated with better OS (*P*<0.001) HR was lower after ctDNA became undetectable during follow-up (adjusted HR: 0.16 (95% CI 0.07–0.36), *P*<0.001). Baseline LDH associated with OS (*P*<0.001)	Levels of circulating tumor DNA (BRAF/NRAS mut) and LDH are associated with PFS, OS and BOR after anti-PD-1 Ab therapy.
Shukla et. al, 2018	**DC** 8 genes (MAGEA3, CSAG3, CSAG2, MAGEA2, MAGEA2B, CSAG1, MAGEA12, MAGEA6) that encode cancer germline antigens associated with lack of clinical benefit to CTLA4 Ab (*P*=0.002) and were increased in patients with PD (median 57-fold increase, range 24–159; maximum *p*= 0.075). **VC**: (median 108-fold increase, 5–236*;* *P*= 0.044	**Kaplan-Meier**: high CRMA expression had PFS than those with low CRMA expression (log-rank *P*=0.006)	**Kaplan-Meier**: high CRMA expression had poorer OS than low CRMA expression (log-rank *P*=0.007). **Multivariate:** CRMA expression predicted poor outcome after ipi (*P*=0.018). CRMA expression was not associated with outcomes of antiPD-1 therapy.	8 genes (MAGEA3, CSAG3, CSAG2, MAGEA2, MAGEA2B, CSAG1, MAGEA12, MAGEA6 are associated with low response and survival after anti-CTLA4 therapy but not antiPD-1 therapy in multivariate analyses.
Simeone et. al, 2014	Disease control associated with decreasing LDH(*P*<0.0001), CRP (*P*<0.0001) and FoxP3/regulatory T cells (*P*=0.003) + increasing ALC (*P*<0.009) between baseline and the end of dosing (Week 12)	N/A	**Univariable:** OS associated with decreasing LDH (*P*<0.0001), CRP (*P*<0.0001) and FoxP3/Tregs (*P*=0.03) + increasing ALC (*P*<0.001) between baseline and Week 12. Survival not associated with BRAF or NRAS status.	Response and OS are associated with decreasing levels of LDH, CRP, FoxP3/Tregs and increasing levels of ALC during treatment with ipilimumab.
Simon et. al, 2017	For 3 patients exhibiting CR and 1 patient with SD, a single emerging Vß subtype represented almost the entire Melan-A–specific repertoire after PD-1 therapy. Amplification of Melan-A–specific Vß subfamilies were undetectable before therapy (thereafter called emerging Vß subfamilies) in responding patients, with predominant expansion in CR. 1 pt with PR, 1 pt with SD and 3 with PD did not show a single Subtype pattern	n/a	n/a	No conclusions can be drawn from this very small study
Simon et. al, 2020	**Baseline:** Differences between responders and non-responders in RLC (*P* = .0322), RNC (*P* = .0247) and RBC (*P*=.0403). **After ICI treatment**: increase of REC (*P*=.0122) and AEC (*P*= 0.0015) seen in responders. RNC decreased *(P* = .0392) in non-responders after first ICI, RBC increased (*P* =0 .0197). Increase of eosinophils after ICI in responders was found (*P* = .0322). AMC and NLR not associated with response.	N/A	N/A	Baseline relative lymphocyte count, relative neutrophil count and relative basophil count are associated with response to immunotherapy. Relative and AEC, relative neutrophil, relative basophil and eosinophils frequency after immunotherapy is associated with response.
Smithy et. al, 2017	Nuclear IRF-1 expression higher in patients with PR or CR than in patients with stable or progressive disease (SD/PD) (*P*=	PFS was higher in the IRF-1-high group than the IRF-1-low group (*P*= 0.017), while PD-L1 expression had no	N/A	Nuclear IRF-1 expression was associated with response and PFS after immunotherapy

Paper	Response	PFS	OS	Interpretation/ conclusion
	0.044). There was a trend toward higher PD-L1 expression in patients with PR/CR (*P*= 0.085)	effect on PFS (*P*= 0.83). In subset analysis, association with PFS is seen in combination ipi / nivo (*P*= 0.0051)		PDL-1 expression had no association with outcomes
Snyder et. al, 2014	Difference in TMB between patients with long-term clinical benefit and minimal benefit or no benefit, both in **DC** (*P*=0.01 Mann–Whitney test) and in **VC** (*P*=0.009 Mann–Whitney test). Specific tetrapeptide neoantigens associated with response (p=0.002). No single mutation associated with response.	N/A	**DC**: high TMB correlated with improved OS (*P*=0.04 by the log-rank test) and there was a trend toward improved survival in the validation set. Specific tetrapeptide neoantigens (*N*=101) were associated with survival (*P*< 0.001) in the discovery and validation sets.	High mutational load is associated with response to anti-CTLA4 Ab therapy. Specific tetrapeptide neoantigens are associated with response and survival.
Subrahmanyam et. al, 2018	**anti-CTLA-4:** responders had lower frequencies of CD45RA+ cells in both CD4+ (*P* = 0.038) and CD8+ (*P* = 0.019) T cells. The converse was also true for CD45RA− cells. **Multivariate**: CD45RA+ and CD45RA− CD4+ and CD8+ T cells + CD8+ T mem cells as predictive markers based on AUC. **anti-PD-1:** No apparent differences in CD45RA expression between responders and non-responders. Responders had higher CD69+ NK cells than non-responders. **Multivariate:** no potential predictive markers were found based on AUC.	N/A	N/A	For anti-CTLA-4, differences were found in CD4+ and CD8+ memory T cells subsets between responders and non-responders. For anti-PD1, responders and non-responders differed in CD69+ and MIP1β+ NK cell populations.
Tarhini et al, 2014	Low pre- and post-treatment tumor CD20+ B cells and change in Treg tended to be associated with worse clinical response (*P* = 0.07 and *P*=0.09).	Greater decrease in moMDSC Lin1−/HLA-DR−/CD33+/CD11b+ associated with improved PFS (*P*= 0.03). Increase in Treg (CD4+CD25hi+Foxp3+) associated with improved PFS (HR = 0.57; *P*= 0.034). CD20+ B cells almost significant (*P*=0.06)		Decrease in monocytic MDSC and increase in Tregs were associated with better PFS but not corrected for confounders. A non-significant association with response for CD20+ and Tregs in ipilimumab.
Tarhini et al, 2015		**Individual analysis:** no significance found. **Modeling analysis**: at baseline, linear combination of [TGF- β1 (ρ = 0.19) and IL-10 (ρ = -0.34)] associated with PFS (HR 2.66; p = 0.035). No correlations with clinical outcomes found in week 6 cytokines.		Combination of baseline TGF- β1 and IL-10 levels were associated with PFS after neoadjuvant ipilimumab.
Tarhini et al,	Pathway analysis identified immune related pathways enriched with genes that associated with clinical outcome at baseline in relation to RFS and early on-treatment. RFS: HR = 2.70, 95% CI (1.1, 6.8). Log rank *P*= 0.029.	N/A	N/A	Pathway analysis identified immune related relevant pathways enriched with genes that associated with outcomes early on-treatment in relation to OS
Tietze et. al, 2017	Patients with baseline lower/equal 25% of CD45RO+CD8+ T cells did not respond to treatment with ipilimumab (*P*<0.01). Levels were not associated with response to pembrolizumab.	N/A	Patients with a normal CD45ROþCD8þ T cells survived longer than low baseline CD45RO+CD8+ T cells (HR 0,29, 95% CI0.1-0.95, *P*=0.05). No correlation with the baseline levels of CD45RO+CD8+ T cells and OS was found.	Low baseline levels of CD45RO+CD8+ T cells are associated with lack of response to ipilimumab. Levels were not associated with response to pembrolizumab.
Tietze et. al, 2016	Low baseline levels of NK cells (*P*<0.01) and CD56dim NK cells (*P*<0.05) and normal baseline levels of CD56bright NK cells(*P*<0.05) correlated significantly with a positive response to ipilimumab but not to pembrolizumab	N/A	Normal baseline levels of CD56bright NK cells were correlated with longer OS compared to high baseline levels (HR 0.2, *p*<0.001).	Low baseline NK cells and CD56dim NK cells are associated with response to ipi but not pembro. Baseline CD56bright NK cells associated with response and OS after ipi.
Topalian et al, 2012	25/42 patients had PD-L1 expression. 36% of PD-L1 expressing patients had PR or CR. None of the patients who did not express PD-L1 had PR or CR. (*P*=0.006)	N/A	N/A	The PD-L1 expression was associated with response rates in a small, non-random subset of patients within a phase I study
Tucci et. al, 2018	mean D-Exo levels calculated before (31.8 8.8%) and after treatment (31.6 10.4%) were not statistically different between responders and nonresponders.	Increment of PD-1 (HR 0.42; 95% C.I. 0.24-0.72; *P*=0.001) and CD28 (HR: 0.51, 95% C.I. 0.3-0.88; *P*= 0.01) in T-cell derived exosomals associated with improved PFS. Increased expression of CD80 and CD86 on DC-derived Exo at end of treatment associated with long PFS (2.73 months, 95% C.I. 2.03-3.57; HR: 0.51, 95% C.I. 0.30-0.89; *P*=0.01).	Increment of PD-1 (HR 0.51; 95%C.I. 0.28-0.91; *P*=0.02) and CD28 (0.48, 95% C.I. 0.27-0.86, *P*= 0.01) expression in T-cell derived exosomes was associated with improved OS.	PD-1 and CD28 T-cell derived exosome expression is associated PFS and OS. CD80 and CD86 DC-derived Exome expression at the end of ipilimumab treatment was associated with PFS. No multivariate analysis.
Tumeh et. al, 2014	At stromal tumor edge and tumour center, responders had higher baseline density of CD8+ cells (*P*<0.0001), PD-1 (*P*=0.0002), PD-L1 (*P*=0.006). CD4 not associated with response. Proximity	N/A	N/A	CD8+ T cell density in the tumour is associated with response to pembrolizumab therapy

Paper	Response	PFS	OS	Interpretation/ conclusion
	of PD-1 and PD-L1 associated with response (*P*=0.005). **Baseline**: expression of phosphostat-1 (pSTST-1) increased in responders (*P*=0,007) as is granzyme B expression on CD8+ cells (*P*<0.0001). **During treatment:** CD8+ cells increase at stromal tumor edges and tumour centers. More restricted TCR beta chain usage (= less diverse T-cell repertoire) associated with response (*P*=0.004). **Logistic regression:** stromal tumor edge CD8+ density predict response, next CD8+ in the tumour center. The predictor was tested in the **validation cohort**: AUC .91 (0.81, 1.00) (*P*<0.0001)			
Urun et. al, J 2019	N/A	N/A	**Univariate**: ALC and LDH (*P*= 0.035) related to OS. **Cox regression**: absolute lymphocyte counts an independent prognostic factor for better survival (*P*<0.005).	Absolute lymphocytes count is associated with survival after ipilimumab in a multivariate analysis.
Uryvaev et. al, 2018	Ratios of CD8+/CD4+ lower than 2 predicted lack of response to treatment (0%) *(P*= 0.006), while CD8+/CD4+ ratios higher than 2.7 had an 81.3% response rate P = 0.0001).	N/A	N/A	Ratios of CD8+/CD4+ are associated with response to antiPD-1 Ab therapy
Valpione et. al, 2015	N/A	N/A	Higher baseline LDH (HR=1.36, 95%CI 1.16–1.58, *P* < .001) and ANC (HR 1.76, 95% CI 1.41–2.10, *P* < .001) associated with worse prognosis. Model performance upon internal (Dxy = 0.42) and external validation (Dxy = 0,4).	Baseline levels of LDH and neutrophils were associated with survival
Van Allen et. al, 2015	**Univariate:** TMB (*P*=0,0076), neoantigen load (*P*=0,027) expression of granzyme A and perforin in immune microenvironment (*P*= 0.042) associated with CB. CTLA4 (*P*= 0.033) and PD-L1 (*P*=0,041) expression associated with response. No recurrent neoantigen peptide sequences predicted response. No genes were enriched for nonsynonymous mutations, including BRAF and NRAS, in the patient subgroups that had CB/no benefit. **Multivariate**: High neoantigen or TMB (>100) more likely to have CB from ipi (*P* = 0.0371 and *P* = 0.0169). Response did not correlate with expression or mutations of HLA, or with BRAF or NRAS	Median time to progression 84 days (range 15-1487)	median OS 272 days (range 34-1632)	Mutational load, neoantigen load and expression of cytolytic markers granzyme A and perforin in the immune microenvironment were associated with clinical benefit in univariate analysis. Clinical factors were not associated with clinical benefit. In multivariable mutational load remained significant.
Varn et. al, 2019	Patients with clinical benefit to anti-PD-1 higher memory B cell-like (MBL) scores (*P*=0.03). MBL scores were higher in clinical benefit after anti-CTLA-4 therapy (*P*=0,02). MBL score was predictive of response at **univariate** and **multivariate** model in both antiPD1 and antiCTLA4 (*P* = 0.04 and 0.03). Multivariate: TBM, CTLA, PD-1, PD-L1 not associated with response.	N/A	MBL score was not associated with prolonged survival in melanoma patients with antiPD1 (*P* = 0.57). In multivariate analysis, higher memory B-cell like scores were only predictive of survival after anti CTLA4 Ab therapy.	Memory B-cell like gene expression signature is associated with clinical benefit after antiPD-1 Ab therapy and clinical benefit and survival after antiCTL4-Ab therapy.
Vilain et. al, 2017	Baseline intratumoral and peritumoral PD-1+ T-cell densities were sevenfold (*P*= 0.006) and fivefold higher (*P*= 0.011) in responders compared with nonresponders. PD-L1 expression at baseline not associated with response, nor CD3+, CD+þ, CD68+, CD20+, and CD8+ intratumoral and peritumoral T-cell counts. PD-L1 in tumor (*P*=0,025) and macrophages (*P*=0,033) and intratumoral CD8+ lymphocytes (*p*=0,046) and CD68+ macrophages (*P*=0,046) elevated in biopsies early during treatment (median 11 days) in responders.	Baseline intratumoral and peritumoral PD-1+ lymphocytes associated with improved PFS. [HR = 0.996; 95%CI, 0.991–1; *P* = 0.062 and HR = 0.996; 95% CI, 0.992–1; *P* = 0.055]. PRE tumor samples exhibiting higher than median intratumoral (>46 cells, PFS 10.3 months vs. 2.7 months, log-rank *P* = 0.015) or peritumoral (>81 cells, PFS 10.2 months vs. 2.7 months, log-rank *P* = 0.039) PD-1+ cell counts associated with PFS.	Baseline intratumoral (*p*=0,021) and peritumoral (*p*=0,009) PD-1+ lymphocytes are associated with improved OS. PRE tumor samples exhibiting higher than median intratumoral (OS not reached months vs. 6 months, log-rank *P* = 0.021) or peritumoral (median OS not reached vs. 6 months, log-rank *P* = 0.009) PD-1+ cell counts were associated with OS.	Baseline intratumoral and peritumoral PD-1+ T-cell densities were associated with response, PFS and OS. PD-L1, CD8+ lymphocytes and CD68+ macrophages elevation in biopsies early during treatment are associated with response.
Wagner et. al, 2018	**Pembrolizumab:** changes in LDH and S100B levels during the first 6 weeks of treatment were different between responders and patients with PD (LDH*: P* = .00088, S100B*: P* = .00091). **Combined ICB:** changes in LDH significantly differed between responders (PR/CR; median: 3.2%, IQR: −15.3% to 25.4%) and	N/A	**Pembrolizumab, multivariate:** elevated baseline S100B or LDH exhibited impaired OS compared with normal S100B (1-year OS: 51.1% vs 83.1%, log-rank *P* < .0001) and normal LDH (1-year OS: 44.4% vs 80.8%, *P*= .00022). **Multivariate:** S100B (HR 2.54, 95% CI 1.20–5.37,	Baseline S100B is associated with OS after antiPD-1 Ab therapy and after combination therapy with anti-PD-1 Ab and anti-CTLA-4 Ab. LDH and S100B increases are also associated with OS for both therapy types.

Paper	Response	PFS	OS	Interpretation/ conclusion
	non-responders (PD; median: 14.2%, IQR: 0.4–58.3%) (*P* = .036). S100B was associated with responders (PR/CR; median: -32.0%, IQR: −60.2% to −6.7%), *P* < .00000.		P = .014), LDH (HR 2.39, 95% CI 1.02–5.59, *P* = .045), visceral metastasis (HR 4.04, 95% CI 1.56–10.45, P = .0039) remained associated. LDH increases >25% S100B increases >145% compared to baseline associated with impaired OS (both *P* < .0001). **Ipi + nivo, univariate:** baseline S100B and increasing S100B levels of >145% + baseline LDH associated with impaired OS (*P* < .0001, *P*= .00060, and P= .0050) increasing LDH of >25% not (*P*= .64). **Multivariate**: baseline S100B remained significant(*p*<0,0001), LDH not for both types of treatment.	S110B and LDH changes were associated with response in both pembrolizumab and combination cohort.
Wagner et. al, 2019	N/A	N/A	**Univariate**: Elevated S100A8/A9 showed impaired OS compared to patients with lower S100A8/A9 treated with pembro (cohort 1 (OS): *P* = .0051; cohort 2 (PFS): *P* < .0001). **Multivariate:** Elevated S100A8/A9 remains significant including LDD >2.5xULN, AJCC M stage.	Serum S100 A8/A9 is associated with survival after pembrolizumab therapy in uni and multivariate analysis
Weber et. al, 2017	N/A	The 'sensitive group' did not have a better PFS [HR=0.63 (95% CI: 0.29-1.36), *P*=0.239]. The ipilimumab-treated validation set showed a significant difference in OS between sensitive and resistant groups (HR=0.40, *P*= 0.004)	**Multivariate:** A signature consisting of 209 proteins/ peptides associated with PFS and OS. A pooled analysis, stratified by set, demonstrated a better OS for patients with a "sensitive" relative to "resistant" protein signature, HR = 0.15 (95%CI 0.06–0.40, *P* < 0.001). Ipi-treated validation set showed difference in OS between sensitive and resistant groups (HR = 0.40, *P* = 0.004). For combination therapy, the signature was not associated with OS.	209 plasma protein signature is associated with OS after anti-PD-1 and anti-CTLA-4, but not combination. A sensitive signature was associated with PFS in ipilimumab.
Weber et. al, 2016	A significant association was observed between response (CR, PR) and stable disease and fewer pretreatment MDSCs (*P* = 0.003).	**Univariate:** There was a significant association between the proportion of MDSCs in peripheral blood before treatment and median and PFS (*P* = 0.002)	**Univariate:** Pretreatment M-MDSCs and OS were inversely associated (*P* = 0.0007) with proportion of MDSCs separated at the median value of 12.6%.	Univariable: in ipilimumab refractory patients treated with nivolumab, fewer MDSC's are associated with better response and survival.
Weide et. al, 2016	N/A	N/A	**Cox regression**: LDH (HR 2.5 95%CI 1.7-3.6, *P*<0.001), relative lymphocyte count (HR 1.9 95%CI 1.3-2.8, *P*<0.001) and relative eosinophil count (HR 2.2 95%CI 1.5-3.1, *P*<0.001) associated with OS in DC and VC combined.	High relative eosinophil count, relative lymphocyte count and low LDH are associated with favorable OS of patients with melanoma treated with pembrolizumab
Wen et. al, 2017	**Pembrolizumab only:** median RLCs and RECs were significantly higher in patients with controlled disease compared to those with progressive disease (29.1 vs. 18.8%, *P*=0.037 for RLC; 3.0 vs. 1.3%, *P*= 0.039 for REC, respectively)	**Multivariate analysis:** baseline LDH (HR 3.2, *P*=0.029) and RLC (HR 2.1 0.034) were independent prognostic factors for PFS (no statistics) after pembrolizumab	**Multivariate analysis: Pembrolizumab:** Baseline LDH (HR 6.4, *P*=0.025) and REC (HR 3.2, *P*=0.016) were independent prognostic factors for OS. (no statistics).	Baseline LDH, RLC and RECs were associated with OS or PFS in uni- and multivariate analyses. Median RLC's and RECs were associated with response.
Wistuba-Hamprecht et. al, 2016	N/A	N/A	**Univariate**: Higher Vdelta1+ cells (>30%) had poorer OS (*P* = 0.043). Higher Vdelta2+ cells (>39%) associated with longer OS (*P* = 0.031). Patients with decreasing frequencies of Vdelta2+ cells under ipi had worse OS (intermediate alterations *p*<0,039; late alterations *P*<0,001). **Multivariate:** for Vδ2+ cells, LDH (HR 1.76, *P*=0.025 and M category (HR 1.68, *P*=0.048) were prognostic for OS. for Vδ1+ cells, LDH predictive of OS (HR 1.83, *P*=0.015).	High frequencies of Vdelta1+ cells and Vdelta2+ cells were associated with OS after ipilimumab therapy in univariate analysis. Decreasing frequencies of Vdelta2+ cells under ipilimumab treatment had worse OS
Wistuba-Hamprecht et. al, 2017	Frequencies (>13%) of CD8 effector-memory type 1 (EM1) T-cells at baseline correlated with higher response rates (*P*= 0.01). Decrease of PD-1 expression on C8 EM1 during 1st, 3rd and after 3rd administration of ipi correlated with BORR (*P*=0.045; *P*=0.007 and P=0.045).	N/A	Frequencies (>13%) of CD8 EM1 T-cells at baseline correlated with longer OS (*p* = 0.029). High baseline frequencies of late stage differentiated EM CD8 cells (>23.8%) negatively associated with OS (*P*= 0.034)	Frequencies of CD8 EM1 T-cells at baseline are associated with survival and response after ipi. Decrease of CD8+ PD-1+EM1 cell during therapy is associated with response.
Wong et. al, 2019	CAF parameters Thy1, SMA and FAB did not correlate with best overall response.	**Univariate**: Thy1 and FAP cell counts had significant positive associations with PFS (all *P* < 0.05). **In**	**Univariate**: Thy1 and FAP cell positive associations with OS (all *P* < 0.003). SMA cell had negative associations with outcome in anti-PD-1 treated patients. **Multivariate**:	Cancer associated fibroblasts (defined by Thy1, smooth muscle actin, and fibroblast activation protein). FAP cells were not

Paper	Response	PFS	OS	Interpretation/ conclusion
		**multivariable analysis** with Thy1, FAB and SMA, only FAB remained associated with PFS (p=0,0031)	revealed associated with OS for CAF parameters, particularly for FAP	associated with response but were associated with improved PFS and OS.
Wong et. al, 2019	Pretreatment infiltrating lymphocytes higher in CR or PR than in SD or progressive disease, particularly for CD8 ($P < 0.0001$). Neither TIL activation nor dormancy was associated with outcome. Predictive performance of CD8 cell count (and QIF) had an area under the ROC curve above 0.75 (ORR/DCR), which reached 0.83 for ipilimumab plus nivolumab.	**Multivariate:** CD8 associations with progression free survival (HR > 3) were significant and accounted for similar CD3 associations in anti–PD-1-treated patients.	**Univariate:** High CD3 cell associated with prolonged OS ($P = 0.0002$). Better OS for immune infiltration independent of absence or presence of TIL activation (all $P < 0.0015$). High CD8 cell count associated with OS of anti–PD-1 ($P < 0.0001$). **Multivariate**: significant CD8 associations (HR > 3; $P < 0.0025$) with OS.	Pretreatment lymphocytic infiltration is associated with response and PFS after immunotherapy. High CD3 and CD8 were associated with OS. TIL activation is not associated with response.
Wood et. al, 2020	TMB, burden of neoepitopes and RNA variants burden are not associated with response (P= 0.267, 0.378 and 0.79 respectively (both therapies combined in one group).	N/A	TMB, Neoepitope burden or its bulk derivatives, including RNA-derived sources of neoepitopes are not significantly associated with OS in melanoma patients treated with diverse immunotherapies.	TMB, Neoepitope burden or its bulk derivatives, including RNA-derived sources of neoepitopes are not associated with response or survival in patients treated anti-PD1 and anti-CLTA4.
Woods et. al, 2020	61 immunophenotypes elevated at baseline in responders formed 5 clusters. **Cluster 1**: CD8+CD4+CD45RA+ CD127+HELIOS–CD73–CD49B–CD38– T cells. **Cluster 5:** CD4+CD95+PD1–CD25– T cells. **Clusters 2 and 4**: CD8+ Tcells expressing LAG3.199 immunophenotypes lower in responders formed 8 clusters, **Clusters 1, 3, 7**: CD4+CD38+ CD39+ CD127– GARP– T cells. **Clusters 2, 4, 5, 6, 8:** CD95+-expressing CD4+ T cells.	N/A	Not clearly reported	In these sub analyses of a randomized trial, 260 immune signatures were associated with response to therapy in patients treated with nivo>ipi and ipi>nivo.
Xiao et. al, 2018	Response was 35% in TP53 wild-type vs 10% in TP53 mutated patients (P=0.049), but this was no longer significant in **multivariate analyses**.	PFS was worse in patients with TP53 mutation vs wild type in both univariate (P=0.017) as **multivariate** analyses (HR 3.49, 95% C.I. 1.68-7.24, P=0.001).	OS was poorer in patients with a TP53 mutation compared to wild type (median 5.5 vs 9.6 months, P=0.04), and remained significant in **multivariate analyses** (HR 3.27, 95% C.I. 1.52-7.02, P=0.002)	TP53 was associated with PFS and OS in patients treated with ipilimumab, but it is questionable if this was mediated through response to treatment.
Yamazaki et. al, 017	IFN-y, IL-6 and IL-10 levels were higher in responders than non-responders (P<0.0001, P=0.0007 and P<0.0001 respectively).	N/A	N/A	In this small study investigating many single biomarkers, IL-6, IL-10 and IFN-y were associated with response to nivolumab.
Yoshida et. al, 2020	N/A	N/A	Overall survival was better in patients with a high CRP (>15.4) in patients treated with nivolumab (P=0.001) and in patients with ipilimumab (P=0.0002).	CRP <15.4 is associated with overall survival in patients treated with ipilimumab or nivolumab monotherapy.
Yuan et a, 2011	**Fisher test**: Patients with CD were 55% of NY-ESO-1–seropositive patients compared with 30% of NY-ESO-1–seronegative patients [P = 0.02, RR 1.8 (1.2–2.8)]. NY-ESO-1–specific CD4+ or CD8+ T-cell response was observed in 16 of 20 (80%) and 13 of 20 (65%) of the NY-ESO-1–seropositive patients	N/A	Trend between measurable NY-ESO-1 response and improved OS (ns) (P = 0.10 and P = 0.06). Seropositive NY-ESO-1 + detectable NY-ESO-1–specific CD8+ T-cell response showed better OS compared with seropositive patients without detectable CD8+ T-cell [P = 0.01, HR 0.18 (0.05–0.71)]. No significance for CD4+T cell/NY-ESO	Serum antibody to NY-ESO-1 is associated with clinical benefit in patients treated with anti–CTLA-4. NY-ESO-1–seropositive patients, with peripheral CD8+ T-cell responses to NY-ESO-1 correlated with clinical benefit and survival.
Yuan et al, 2014	**N/A**	N/A	**Multivariate:** Pretreatment VEGF ≥ 43 pg/mL associated with decreased OS (median OS 6.6 vs. 12.9 months, P = 0.006; 7.4 vs. 14.3 months, P=0.037 for 3 mg/kg; and 6.2 vs. 10.9 months, P=0.048 for 10 mg/kg). No significance for changes during treatment.	Pretreatment levels op VEGF are associated with overall survival in multivariate analysis
Yusko et. al, 2019	Within arm A (nivo/ipi), TMB at baseline trend towards significant best response (N=30, P=.06) and neoantigen burden at baseline associated with best response at week 33 (N=30, P=.05). No associations for TMB or neoantigen load seen for arm B (ipi/nivo), for which a lower response / shorter OS observed compared to arm A. For arm A (nivo/ipi), pretreatment TIL clonality associated with response (P=.04). T-cell fraction correlated with response in combined data of both arms (P=.02, N=89); due to reduced statistical power, not significantly correlated with response in arm A (P= .21, N = 39) or B (P =	N/A	Patients with increased T-cell fraction post-treatment at week 13 had a 30-fold increased likelihood of survival (P = .002). Mutational and neoantigen load, and T-cell infiltrate within the tumor, were (however not significantly) associated with outcome of sequential checkpoint inhibition using nivolumab then ipilimumab, but not when ipilimumab was administered before nivolumab.	Baseline T cell infiltration is marginally associated with response after nivolumab. PD-L1 expression on tumour is associated with response to nivolumab.

Paper	Response	PFS	OS	Interpretation/ conclusion
	.07, *N* = 50). PD-L1 correlated with CB (PD-L1 ≥ 5%, *P* = .009, OR = 14.7 Fisher's exact test). At week 13, individuals with increased T-cell fraction and TIL clonality were 30c more likely to achieve best clinical response at week 33 (*P*=.002; OR =30).			
Zaragoza et. al, 2016	N/A	N/A	**Univariate:** high NLR associated with poor OS (HR 1.17, 95% 1.09-1.26) and remained significant in **multivariable** (HR = 1,21, 95% C.I. 1,07–1,36. LDH 2x above normal associated with reduced OS (HR = 4,86; 95% CI 1,98–11,93, *P* < 0,001, but not in multivariate analysis	The neutrophil to lymphocyte ratio is associated with survival after ipilimumab therapy
Zhou et. al, 2017	High pretreatment sPD-L1 associated with PD in patients treated by CTLA-4 (DC *P*= 0.0015, VC *P*=0.04). No association between levels of PD-L1 and response to anti PD1. Changes in circulating sPD-L1 early after treatment could not distinguish responders from PD, but 1.5x increases in sPD-Ll after 5 months of treatment with ipi associated with PR, compared with patients with <1.5-fold increases (75% vs. 27%, *P*= 0.039. Results for anti-PD-1 cohort: All 8 patients with 1.5-fold increases in sPDL1 after 5 months experienced PR, *P*= 0.007.	N/A	N/A	Soluble PD-L1 levels at baseline are associated with response to anti-CTLA-4 an perhaps also in patients with anti-PD1 but these patient numbers were small. Changes in soluble PD-L1 levels might be associated with responses but these analyses were not clearly explained.

Abbreviations: ALC: Absolut lymphocyte count, ANC: absolute neutrophil count, AEC; absolute eosinophil count, AMC: absolute monocyte count, BOR®: best overall response (rate), CAF: cancer associated fibroblasts, CB; clinical benefit, CR: complete response, CRP: C reactive protein, ctDNA: circulating tumor DNA, CTC: circulating tumor cells, DC: development cohort, ESR: erythrocyte sedimentation rate, GEP: gene expression profiling test, HR: hazard ratio, IL: interleukin, IPI: ipilimumab, LDH: lactate dehydrogenase, MDSC: myeloid derived suppressor cells, MNR: not reported, MSS: Melanoma-Specific Survival N/A: not applicable, NIVO: nivolumab, NLR: neutrophil to lymphocyte ratio, ORR: overall response rate, OS, overall survival, PFS, progression free survival, PD: progressive disease, PEMBRO: pembrolizumab, P/LP Pathologic or likely pathologic**,** PR: partial response, PS performance score, RBC: red blood cell count, REC; relative eosinophil count, RLC: relative lymphocyte count, SD: stable disease, SNP: single nucleotide polymorphism, TIL: tumor infiltrating lymphocyte; TMB: tumor mutational burden, VC: validation cohort, WBC: white blood cell count,

Table S3 - Summary of peripheral blood biomarkers that were studied for anti-CLTA-4 therapy and assessed risk of bias per article.

Anti-CLTA4	Biomarker in blood	Studies	Patients	Response	PFS	OS	Quality assessment
**Blood count / cytology**	White blood cell count (WBC)	6	698	WBC was associated with response in 1/5 studies but not associated with response in 4/5 studies.	WBC was associated with PFS in 1/2 studies, but not associated in 1/2 studies.	WBC was associated with OS in 1/3 studies, but not associated in 2/3 studies	1/6 high risk, 3/6 moderate risk, 2/6 low risk of bias.
	Lymphocyte count (LC)	16	1995	LC was associated with response in 3/9 studies, not associated with response in 6/9 studies.	LC was not associated with PFS in 2/2 studies.	LC was associated with OS in 6/11 studies, not associated for OS in 5/11 studies.	2/16 high risk, 10/16 moderate risk 4/16 low risk of bias.
	Eosinophil count (EC)	7	1020	EC was associated with response in 1/4 studies, not associated in 3/4 studies.	EC was not associated with PFS in 1/1 studies.	EC was associated with OS in 3/5 studies, not associated in 2/5 studies.	2/7 high risk, 5/7 moderate risk 0/7 low risk of bias.
	Absolute neutrophil count (ANC)	9	1455	ANC was associated with response in 2/4 studies, not associated with response in 2/4 studies.	ANC was associated with PFS in 2/2 studies.	ANC was associated with OS in 3/6 studies, not associated with OS in 3/6 studies.	2/9 high risk 3/9 moderate risk, 4/9 low risk of bias.
	Absolute monocyte count (AMC)	2	674	AMC was not associated with response in 1/1 studies.		AMC was associated with OS in 1/1 studies.	1/2 high risk 1/2 moderate risk, 0/2 low risk of bias
	monocytic MDSCs	4	168	moMDSC was associated with response in 2/2 studies.	moMDSC was associated with PFS in 2/2 studies	moMDSC was associated with OS in 1/1 studies.	3/4 high risk 1/4 moderate risk, 0/4 low risk of bias
	MDSCs	4	726	MDSCs were associated with response in 3/3 studies.		MDSCs were associated with OS in 2/2 studies.	1/4 high risk, 3/4 moderate risk, 0/3 low risk of bias
	Platelet-to-lymphocyte ratio (PLR)	2	235	PLR was associated with response in 1/2 studies, not associated with response in 1/2 studies.	PLR was not associated with PFS in 1/1 studies.	PLR was associated with OS in 1/1 studies	0/2 high risk 2/2 moderate risk, 0/2 low risk of bias

Anti-CLTA4	Biomarker in blood	Studies	Patients	Response	PFS	OS	Quality assessment
	Neutrophil-to-lymphocyte ratio (NLR)	11	1632	NLR was associated with response in 3/5 studies, not associated in 2/5 studies.	NLR was associated with PFS in 4/5 studies, not associated in 1/5 studies.	NLR was associated with OS in 7/10 studies, not associated with OS in 3/10 studies	1/11 high risk, 6/11 moderate risk, 4/11 low risk of bias
	Eosinophil-to-lymphocyte ratio (ELR)	3	282	ELR was not associated with response in 3/3 studies.	ELR was not associated with PFS in 2/2 studies.	ELR was not associated with OS in 2/2 studies.	0/3 high risk, 3/3 moderate risk 0/3 and low risk of bias
	Lymphocyte-to-monocyte ratio (LMR)	1	133		LMR was associated with PFS in 1/1 studies.	LMR was associated with PFS in 1/1 studies.	0/1 high risk, 0/1 moderate risk, 1/1 low risk of bias
	Thrombocytes	1	52	Thrombocytes were not associated with response in 1/1 studies.	Thrombocytes were not associated with PFS in 1/1 studies.		0/1 high risk, 1/1 moderate, risk 0/1 low risk of bias
	Natural killer (NK) cells	2	63	NK cells were associated with response in 1/2 studies, but not associated in 1/2 studies.			1/2 high risk, 1/2 moderate, risk 0/2 low risk of bias
**Soluble factors**	LDH	20	2539	LDH was associated with response in 4/10 studies, not associated in 6/10 studies.	LDH was associated with PFS in 3/3 studies.	LDH was associated with OS in 12/16 studies, not associated in 4/16 studies.	2/20 high risk, 14/20, moderate, 4/20 low risk of bias
	Anemia	1	97			Anemia was not associated with OS in 1/1 studies.	0/1 high risk, 1/1 moderate risk, 0/1 low risk of bias
	CRP	6	493	CRP was associated with response in 1/2 studies, not associated in 1/2 studies.	CRP was not associated with PFS in 2/2 studies.	CRP was associated with OS in 2/4 studies, not associated in 2/4 studies	1/6 high risk, 4/6 moderate risk, 1/6 low risk of bias
	Erythrocyte sedimentation rate (ESR)	2	277	ESR was not associated with response in 1/1 studies.		ESR was associated with OS in 1/2 studies, not associated with OS in 1/2 studies.	0/2 high risk, 2/2 moderate risk, 0/2 low risk of bias
	Albumin	1	134			Albumin was associated with OS in 1/1 studies.	0/1 high risk, 1/1 moderate risk, 0/1 low risk of bias
	Alkaline phosphatase (AP)	1	215			AP was not associated with OS in 1/1 studies.	0/1 high risk, 0/1 moderate risk, 1/1 low risk of bias
	Interleukin-2 (IL-2)	3	290	IL-2 was not associated with response 1/1 studies.	IL-2 was not associated with PFS in 1/1 studies.	IL-2 was not associated with OS in 1/1 studies.	0/3 high risk, 2/3 moderate risk 1/3 low risk of bias
	Interleukin-6 (IL-6)	3	290	IL-6 was associated with response 1/1 studies.	IL-6 was not associated with PFS in 1/1 studies.	IL-6 was not associated with OS in 2/2 studies.	0/3 high risk, 2/3 moderate risk 1/3 low risk of bias
	IFN-gamma	1	35		IFN-g not associated with PFS in 1/1 studies.		0/1 high risk, 1/1 moderate risk, 0/1 low risk of bias
	MICA antibodies	1	77	Anti-MICA was not associated with response in 1/1 studies.		Anti-MICA was not associated with OS in 1/1 studies.	0/1 high risk, 0/1 moderate risk, 1/1 low risk of bias
	HMGB1 protein (cytokine)	1	59	HMGB1 was associated with response in 1/1 studies.			1/1 high risk, 0/1 moderate risk, 0/1 low risk of bias
	T-cell derived exosomes	1	59	D-exos were not associated with response in 1/1 studies.	D-exos were associated with PFS in 1/1 studies.	D-exos were associated with PFS in 1/1 studies.	0/1 high risk, 0/1 moderate risk 1/1 low risk of bias
	S100	5	633	S100 was associated with response in 2/3 studies and not associated in 1/3 studies.	S100 was associated with MSS in 1/1 studies, not associated with PFS in 1/1 studies.	S100 was associated with for OS in 1/3 studies and not associated with OS in 2/3 studies.	0/5 high risk, 3/5 moderate risk, 2/5 low risk of bias
	Vitamin D	1	52	Vit D was not associated with response in 1/1 studies.	Vit D was not associated with PFS in 1/1 studies.		0/1 high risk, 1/1 moderate risk, 0/1 low risk of bias
	VEGF	2	426		VEGF was not associated with PFS in 1/1 studies.	VEGF was associated with OS in 1/2 studies, not associated in 1/2 studies.	0/2 high risk 2/2 moderate risk, 1/2 low risk of bias
	Soluble CTLA-4	1	113	sCTLA-4 was associated with response in 1/1 studies.		sCTLA-4 was associated with OS in 1/1 studies.	0/1 high risk, 1/1 moderate risk, 0/1 low risk of bias

Anti-CLTA4	Biomarker in blood	Studies	Patients	Response	PFS	OS	Quality assessment
	Soluble PD-L1	1	193	sPD-L1 was associated with response in 1/1 studies.			0/1 high risk, 1/1 moderate risk, 0/1 low risk of bias
	Soluble receptor tyrosine kinase AXL	1	53			sAXL was associated with OS in 1/1 studies.	1/1 high risk, 0/1 moderate risk, 0/1 low risk of bias
	Kynurenine-tryptophan ratio	1	78			Kyn/tript ratio was associated with OS in 1/1 studies.	1/1 high risk, 0/1 moderate risk, 0/1 low risk of bias
	MMP-degraded collagens	1	66	Collagen biomarkers were associated with response in 1/1 studies.	Collagen biomarkers were not associated with PFS in 1/1 studies.	Collagen biomarkers were associated with OS in 1/1 studies.	0/1 high risk, 0/1 moderate risk, 1/1 low risk of bias
	Type III collagen formation (PRO-C3)	1	66	PRO-C3 was associated with response in 1/1 studies.		PRO-C3 was associated with OS in 1/1 studies.	0/1 high, 0/1 moderate risk, 1/1 low risk of bias
	Plasma protein signature	1	46		Plasma protein signature was associated with PFS in 1/1 studies.	Plasma protein signature was associated with OS in 1/1 studies.	0/1 high risk, 0/1 moderate risk, 1/1 low risk of bias
**Soluble tumor factors**	Melanoma inhibitory activity	1	77	MIA was not associated with response in 1/1 studies.		MIA was not associated with OS in 1/1 studies.	0/1 high risk, 0/1 moderate risk, 1/1 low risk of bias
	β2-microglobulin	1	215			B2-M was not associated with OS in 1/1 studies.	0/1 high risk, 0/1 moderate risk, 1/1 low risk of bias
	NY-ESO antibody	1		NY-ESO was associated with response in 1/1 studies.		NY-ESO was associated with OS in 1/1 studies.	1/1 high risk, 0/1 moderate risk, 0/1 low risk of bias
**T cell regulation / tumor infiltration**	FoxP3/regulatory T-cells	3	741	Treg was associated with response in 1/1 studies.	Treg was associated with RFS in 1/1 studies	Treg was associated with OS in 2/2 studies.	1/3 high risk, 2/3 moderate risk, 0/3 as low risk of bias
	ICOS expression in CD8 and CD4 cells	1	17	ICOS expression was associated with response in 1/1 studies.		ICOS expression was associated with OS 1/1 studies.	0/1 high risk, 1/1 moderate risk, 0/1 as low risk of bias
	Naïve CD4 and CD8 T-cells	2	99	CD4 T cells were not associated with response in 1/1 studies.		Naive CD4 cells were not associated with OS in 2/2 studies.	0/2 high risk, 2/2 moderate, 0/2 as low risk of bias
	CD4 effector-memory T-cells	1	17			CD4 effector memory cells were not associated with OS in 1/1 studies.	0/1 high risk, 1/1 moderate, 0/1 low risk of bias
	CD45RA+ memory T-cells	2	64	CD45RA+ was associated with response in 1/2 studies, not associated in 1/2 studies.			0/2 high risk 2/2 moderate risk 0/2 low risk of bias
	CD8 (effector) memory T-cells	3	90	CD8 memory T cells were associated with response in 2/2 studies.		CD8 memory cells were associated with OS in 3/3 studies.	2/3 high risk, 1/3 moderate risk 0/1 low risk of bias
	CD4 and 8 EMRA T-cells	1	17			CD4 and 8 EMRA cells were associated with OS in 1/1 studies.	0/1 high risk, 1/1 moderate risk, 0/1 low risk of bias
	PD-L1 on CD4+ and CD8+ T-cells	1	190	PDL1+CD4+ and PDL1+CD8 were not associated with response in 1/1 studies.	PDL1+CD4+ were associated with PFS in 1/1 studies, not associated in 1/1 studies	PDL1+CD8+ was associated with OS in 1/1 studies, not associated in 1/1 studies	1/1 high risk, 0/1 moderate risk, 0/1 low risk of bias
	γδ1+ and 2+ T-cells	1	109			γδ1 T cells were associated with OS in 1/1 studies.	0/1 high risk, 1/1 moderate risk, 0/1 low risk of bias
	T-cell receptor (TCR) diversity	2	54	TCR diversity was associated with response in 2/2 studies.		TCR diversity was not associated with OS in 1/1 studies.	1/2 high risk, 1/2 moderate risk, 0/2 low risk of bias
	SNPs for autoimmune diseases	1	215	SNPs were not associated with response in 1/1 studies.			0/1 high risk, 1/1 moderate risk, 0/1 low risk of bias

Abbreviations; CRP: C-Reactive Protein, ICOS: Inducible T-cell COStimulator, LDH: lactate dehydrogenase, MICA: MHC class I-related chain A, MDSCs: myeloid-derived suppressor cells, MMP: matrix metalloproteinases, SNP: single-nucleotide polymorphism, VEGF: Vascular endothelial growth factor

Table S4: Summary of peripheral blood biomarkers that were studied for anti-PD-1 therapy and assessed risk of bias per article

Anti-PD1	Biomarker in blood	Studies	Patients	Response	PFS	OS	Quality assessment
**Blood count / cytology**	White blood cell count (WBC)	9	1469	WBC was not associated with response in 4/4 studies.	WBC was associated with PFS in 1/4 studies, not associated with PFS in 3/4 studies.	WBC was associated with OS in 1/5 studies. WBC not associated in 4/5 studies.	3/9 high risk, 5/9 moderate, 1/9 low risk of bias
	Absolute neutrophil count (ANC)	11	1261	ANC was not associated with response in 5/5 studies.	ANC was associated with PFS in 4/5 studies, not associated in 1/5 studies.	ANC was associated with OS in 2/3 studies, not associated in 1/3 studies.	5/11 high risk, 5/11 moderate, 1/11 low risk of bias
	Absolute basophil count (ABC)	2	56	ABC was not associated with response in 2/2 studies.			1/2 high risk, 1/2 moderate, 0/2 low risk of bias
	Lymphocyte count (LC)	14	1644	LC was associated with response in 3/6 studies, not associated in 3/6 studies.	LC was associated with PFS in 4/8 studies, not associated in 4/8 studies.	LC was associated with OS in 4/8 studies, not associated with OS in 4/8 studies.	6/14 high risk, 7/14 moderate, 1/14 low risk of bias
	Neutrophil-to-lymphocyte ratio (NLR)	8	732	NLR was associated with response 1/3 studies, not associated in 2/3 studies.	NLR was associated with PFS in 5/5 studies.	NLR was associated with OS in 6/6 studies.	1/8 high risk, 6/8 moderate, 1/8 low risk of bias
	Leucocyte-to-lymphocyte ratio (LLR)	2	159		LLR was associated with PFS in 1/2 studies, not associated 1/2 studies	LLR was associated with OS in 1/1 studies.	0/2 high risk, 1/2 moderate, 1/2 low risk of bias
	Absolute monocyte count (AMC)	7	480	AMC was associated with response in 1/4 studies, not associated in 3/4 studies.	AMC was associated with PFS in 1/3 studies. AMC not associated in 2/3 studies.	AMC was associated with OS in 1/3 studies, not associated with OS in 2/3 studies	5/7 high risk, 2/7 moderate, 0/7 low risk of bias
	MDSCs	1	92	MDSCs were associated with response in 1/1 studies.	MDSCs were associated with PFS in 1/1 studies.	MDSCs were associated with OS in 1/1 studies.	0/1 high risk, 1/1 moderate, 0/1 low risk of bias
	Eosinophil count (EC)	11	1325	EC was associated with response in 1/5 studies, not associated in 4/5 studies.	EC was associated with PFS in 1/5 studies, not associated with PFS in 4/5 studies	EC was associated with OS in 3/6 studies, not associated with OS in 3/6 studies	6/11 high risk, 3/11 moderate, 2/11 low risk of bias
	Thrombocytes	2	143	Platelets were not associated with response in 1/1 studies.	Platelets were associated with disease progression in 1/1 studies.		0/2 high risk, 1/2 moderate, 1/2 low risk of bias
	Platelet-to-lymphocyte ratio (PLR)	1	39	PLR was not associated with response in 1/1 studies.			0/1 high risk, 1/1 moderate, 0/1 low risk of bias
	NK cells	5	128	NKs were associated with response in 3/4 studies, not associated in 1/4 studies.		NK cells were not associated with OS in 2/2 studies.	4/5 high risk, 1/5 moderate, 0/5 low risk of bias
	Natural killer T (NKT) cells	2	37	NKT cells were associated with response in 2/2 studies.			2/2 high risk, 0/2 moderate, 0/2 low risk of bias
**Soluble circulating factors**	LDH	20	2274	LDH was associated with response in 4/10 studies, not associated in 6/10 studies.	LDH was associated with PFS in 10/11 studies, not associated in 1/11 studies.	LDH was associated with OS in 13/13 studies.	5/20 high risk, 12/20 moderate, 3/20 low risk of bias
	5-S-cysteinydopa (5-S-CD)	1	18	5-S-CD was not associated with response in 1/1 studies.			1/1 high risk, 0/1 moderate, 0/1 low risk of bias
	S100A/ S100B	3	261	S100B was associated with response in 1/2 studies, not associated in 1/2 studies.		S100A and S100B associated with OS in 2/2 studies.	0/3 high risk, 2/3 moderate, 1/3 low risk of bias
	CRP	8	486	CRP was associated with response in 1/2 studies, not associated in 1/2 studies.	CRP was associated with PFS in 1/4 studies, not associated in 3/4 studies	CRP was associated with OS in 2/5 studies, not associated in 3/5 studies.	2/8 high risk, 5/8 moderate, 1/8 low risk of bias
	CRP-to-Albumin ratio (CAR)	1	39		CAR was associated with PFS in 1/1 studies.		0/1 high risk, 1/1 moderate, 0/1 low risk of bias

Anti-PD1	Biomarker in blood	Studies	Patients	Response	PFS	OS	Quality assessment
	Erythrocyte Sedimen-tation Rate (ESR)	1	18	ESR was associated with response in 1/1 studies.			1/1 high risk, 0/1 moderate, 0/1 low risk of bias
	Hepatocyte Growth Factor (HGF)	2	64	HGF was associated with response in 1/2 studies, not associated in 1/1 studies.	HGF was associated with PFS in 1/1 studies.	HGF was associated with OS in 1/1 studies.	1/2 high risk, 1/2 moderate, 0/2 low risk of bias
	Interleukin-6 (IL-6)	1	35	IL-6 was associated with response in 1/1 studies.			1/1 high risk, 0/1 moderate, 0/1 low risk of bias.
	Interleukin-8 (IL-8)	2	64	IL-8 was associated with response in 1/2 studies, not associated in 1/2 studies.		IL-8 was associated with OS in 1/1 studies.	1/2 high risk, 1/2 moderate, 0/2 low risk of bias
	Interleukin-9 (IL-9)	1	46	IL-9 was associated with response in 1/1 studies.			1/1 high risk, 0/1 moderate, 0/1 low risk of bias
	Interleukin-10 (IL-10)	3	72	IL-10 was associated with response in 3/3 studies.			3/3 high risk, 0/3 moderate, 0/3 low risk of bias
	IFN-γ	3	101	IFN-γ was associated with response in 2/3 studies, not associated in 1/3 studies.			3/3 high risk, 0/3 moderate, 0/3 low risk of bias
	TGF-b	2	81	TGF-b was associated with response in 1/2 studies, not associated in 1/2 studies			2/2 high risk, 0/2 moderate, 0/2 low risk of bias.
	CXCL5 (chemokine)	1	46	CXCL5 was associated with response in 1/1 studies.			1/1 high risk, 0/1 moderate, 0/1 low risk of bias
	CXCL10 (chemokine)	2	63	CXCL10 was associated with response in 1/2 studies, not associated in 1/2 studies.			2/2 high risk, 0/2 moderate, 0/2 low risk of bias
	CCLL22 (chemokine)	1	46	CCLL22 was not associated with response in 1/1 studies.			1/1 high risk, 0/1 moderate, 0/1 low risk of bias
	Soluble CD73	1	37	sCD73 was associated with response in 1/1 studies.	sCD73 was associated with PFS in 1/1 studies.	sCD73 was associated with OS in 1/1 studies.	0/1 high risk, 1/1 moderate, 0/1 low risk of bias
	Soluble CD163	1	75	sCD163 was associated with response in 1/1 studies.			0/1 high risk, 1/1 moderate, 0/1 low risk of bias
	Exosomal circulating PD-L1	2	62	Exo PD-L1 was associated with response in 1/2 studies, not associated with response in 1/2 studies.			2/2 high risk 0/2 moderate, 0/2 low risk of bias
	Total soluble PD-L1	2	79	Total PD-L1 was not associated with response in 2/2 studies.			1/2 high risk, 1/2 moderate, 0/2 low risk of bias
	Microvescicle PD-L1	1	44	mPD-L1 was not associated with response in 1/1 studies.			1/1 high risk, 0/1 moderate, 0/1 low risk of bias
	Plasma proteomics	2	95		Some proteomics were associated with PFS in 2/2 studies.		0/2 high risk, 2/2 moderate, 0/2 low risk of bias
	Plasma protein signature	1	220		Plasma protein signature was not associated with PFS in 1/1 studies.	Plasma protein signature was associated with OS in 1/1 studies.	0/1 high risk, 1/1 moderate, 0/1 low risk of bias
**Soluble tumor factors**	circulating tumor DNA (ctDNA)	1	85		ctDNA was associated with PFS in 1/1 studies.	ctDNA was associated with OS in 1/1 studies.	0/1 high risk, 1/1 moderate, 0/1 low risk of bias
	PD-L1 expression on circulating tumor cells	1	40	PD-L1+ CTCs was associated with response in 1/1 studies.	PD-L1+ CTCs was associated with PFS in 1/1 studies.		0/1 high risk, 1/1 moderate, 0/1 low risk of bias
**T cell regulation /**	CD8 memory T-cells	2	29	CD8 memory cells were associated with response in 1/2 studies, not associated in 1/2 studies.		CD8 memory cells were not associated with OS in 1/1 studies.	2/2 high risk, 0/2 moderate, 0/2 low risk of bias

Anti-PD1	Biomarker in blood	Studies	Patients	Response	PFS	OS	Quality assessment
**tumor infiltration**	CD45RA+ memory T-cells	4	119	CD45RA+ was associated with response in 2/4 studies, not associated in 2/4 studies.			3/4 high risk, 1/4 moderate, 0/4 low risk of bias
	Total CD8 cells	4	183	CD8 cells was associated with response in 2/4 studies, not associated in 2/4 studies.		CD8 cells were not associated with OS in 1/1 studies.	3/4 high risk, 1/4 moderate, 0/4 low risk of bias
	Regulatory T-cells	1	46	Tregs were not associated with response in 1/1 studies.			1/1 high risk, 0/1 moderate, 0/1 low risk of bias
	CD4+PD1 lymphocytes	3	128	CD4+PD1 cells was associated with response in 1/2 studies, not associated in 1/2 studies.		CD4+PD1 cells was not associated with OS in 1/1 studies.	2/3 high risk, 1/3 moderate, 0/3 low risk of bias
	CD8+PD1 lymphocytes	4	228	CD8+PD1 cells were not associated with response in 3/3 studies.		CD8+PD1 cells were not associated with OS in 2/2 studies.	2/4 high risk, 2/4 moderate, 0/4 low risk of bias
	CD8+CD73+ lymphocytes	1	100	CD8+CD73 were not associated with response in 1/1 studies.		CD8+CD73 cells were not associated with OS in 1/1 studies.	0/1 high risk, 1/1 moderate, 0/1 low risk of bias
	CD8+PD-1+CD73+lymphocytes	1	100	CD8+PD1+CD73 were associated with response in 1/1 studies.		CD8+PD1+CD73 were associated with OS in 1/1 studies.	0/1 high risk, 1/1 moderate, 0/1 low risk of bias
	PD-1+ expression on CD56+ T cells	1	75	PD-1+CD56+ were associated with response in 1/1 studies.	PD-1+CD56+ cells were associated with PFS in 1/1 studies.	PD-1+CD56+ cells were associated with OS in 1/1 studies.	0/1 high risk, 1/1 moderate, 0/1 low risk of bias
	T cell receptor (TCR) diversity	1	38	TCR diversity was associated with response in 1/1 studies.			0/1 high risk, 1/1 moderate, 0/1 low risk of bias
	Ki67 (exhausted T cells)	2	89	Ki67 cells were associated with response in 1/2 studies, not associated in 1/2 studies.	Ki67 cells were associated with PFS in 1/1 studies.	Ki67 cells were associated with OS in 1/1 studies.	1/2 high risk, 1/2 moderate, 0/2 low risk of bias
	TIM3 on T-cells	2	60	TIM3 was associated with response in 1/2 studies, not associated in 1/2 studies.			2/2 high risk, 0/2 moderate, 0/2 low risk of bias
	LAG-3 on T-cells	1	18	LAG-3 was not associated with response in 1/1 studies.			1/1 high risk, 0/1 moderate, 0/1 low risk of bias
**Other**	SNPs associated with autoimmune diseases	1	176	SNP rs17388568 was associated with response in 1/1 studies.			0/1 high risk, 1/1 moderate, 0/1 low risk of bias

Abbreviations; CRP: C-Reactive Protein, IFN: interferon, LAG3: Lymphocyte Activating 3, LDH: lactate dehydrogenase, MDSCs: myeloid-derived suppressor cells, NK: natural killer, SNP: single-nucleotide polymorphism, TGF: tumor growth factor.

Table S5: Summary of peripheral blood biomarkers that were studied for mixed therapy cohorts and assessed risk of bias per article

Mixed cohorts	Biomarker in blood	Studies	Patients	Response	PFS	OS	Quality assessment
**Blood count / cytology**	Absolute lymphocyte count (ALC)	2	112	ALC was not associated with response in 1/1 studies.	ALC was associated with PFS in 1/1 studies.		1/2 high risk, 1/2 moderate ,0/2 low risk of bias
	Relative lymphocyte count (RLC)	1	32	RLC was associated with response in 1/1 studies.			1/1 high risk, 0/1 moderate 0/1 low risk of bias
	Absolute eosinophil count (AEC)	2	311	AEC was associated with response in 1/1 studies.		AEC was associated with OS in 1/1 studies.	2/2 high risk, 0/2 moderate, 0/2 low risk of bias
	Absolute neutrophil count (ANC)	2	112	ANC was not associated with response in 1/1 studies.	ANC was associated with PFS in 1/1 studies.		1/2 high risk, 1/2 moderate, 0/2 low risk of bias
	Relative neutrophil count (RNC)	1	32	RNC was associated with response in 1/1 studies.			1/1 high risk, 0/1 moderate 0/1 low risk of bias

	Relative basophil count (RBC)	1	32	RBC was associated with response in 1/1 studies.		.	1/1 high risk, 0/1 moderate, 0/1 low risk of bias
	Absolute monocyte count (AMC)	1	32	AMC was not associated with response in 1/1 studies.			1/1 high risk, 0/1 moderate, 0/1 low risk of bias
	Neutrophil-to lymphocyte ratio (NLR)	1	32	NLR was not associated with response in 1/1 studies.		1/1 high risk, 0/1 moderate, 0/1 low risk of bias	1/2/ high risk, 1/2 moderate 0/2 low risk of bias
**Soluble circulating factors**	LDH	2	141	LDH was not associated with response in 1/1 studies.	LDH was associated with PFS in 1/1 studies	LDH was associated with OS in 1/2 studies, not associated with OS in 1/2 studies.	0/2 high risk, 2/2 moderate, 0/2 low risk of bias
	CXCL9 / CXCL10 (chemokines)	1	28	CXCL9 and CXCL10 were associated with response in 1/1 studies.			1/1 high risk, 0/1 moderate 0/1 low risk of bias
	ULBP-1 (receptor NK ligands)	1	162	ULBP levels were associated with response in 1/1 studies.		ULBP levels were associated with OS in 1/1 studies.	0/1 high risk, 1/1 moderate, 0/1 low risk of bias
	S100B	3	196	S100B was associated with response in 1/2 studies, not associated in 1/2 studies,	S100B was associated withS in 1/3, and PFS in 1/3 studies, but not with PFS in 1/3 studies.	S100B was associated with OS in 2/2 studies.	1/3 high risk, 2/3 moderate, 0/3 low risk of bias
	IgG1, IgG3, Total IgG	1	49	IgGs were not associated with response in 1/1 studies.	IgGs were associated with PFS in 1/1 studies.	IgGs were not associated with OS in 1/1 studies.	0/1 high risk 0/1 moderate, 1/1 low risk of bias
	IgG2	1	49	IgG2 was associated with response in 1/1 studies.	IgG2 was associated with PFS in 1/1 studies.	IgG2 was associated with OS in 1/1 studies.	0/1 high risk 0/1 moderate, 1/1 low risk of bias
	IgG4	1	49	IgG4 was not associated with response in 1/1 studies.	IgG4 was not associated with PFS in 1/1 studies.	IgG4 was not associated with OS in 1/1 studies.	0/1 high risk 0/1 moderate, 1/1 low risk of bias
	IgG against melanocytes: TRP/TYRP 1 and 2	1	41	TRP/TYRP was associated with response in 1/1 studies.	TRP/TYRP was not associated with PFS in 1/1 studies.	TRP/TYRP was not associated with OS in 1/1 studies.	1/1 high risk, 0/1 moderate 0/1 low risk of bias
	IgG against melanocytes: MelanA	1	41	MelanA was associated with response in 1/1 studies.	MelanA was associated with PFS in 1/1 studies.	MelanA was associated with OS in 1/1 studies.	1/1 high risk, 0/1 moderate 0/1 low risk of bias
	IgG against melanocytes: GP100	1	41	GP100 was not associated with response in 1/1 studies.	GP100 was associated with PFS in 1/1 studies.	GP100 was associated with OS in 1/1 studies.	1/1 high risk, 0/1 moderate 0/1 low risk of bias.
	IgG against melanocytes: NY-ESO1	1	41	NY-ESO1 was associated with response in 1/1 studies.	NY-ESO1 was not associated with PFS in 1/1 studies.	NY-ESO1 was associated with OS in 1/1 studies.	1/1 high risk, 0/1 moderate 0/1 low risk of bias
	Circulating exosomal PD-L1	1	100	Circ exosomal PD-L1 was associated with response in 1/1 studies.	Circ exosomal PD-L1 was associated with PFS in 1/1 studies.	Circ exosomal PD-L1 was associated with OS in 1/1 studies.	0/1 high risk, 1/1 moderate, 0/1 low risk of bias
	Plasma cholesterol	1	28		High cholesterol was associated with PFS in 1/1 studies.	High cholesterol was associated with OS in 1/1 studies.	1/1 high risk, 0/1 moderate 0/1 low risk of bias
**Tumor factors**	circulating tumor DNA (ctDNA)	3	190	ctDNA was associated with response in 3/3 studies.	ctDNA was associated with PFS in 3/3 studies.	ctDNA was associated with OS in 2/2 studies.	1/3 high risk, 2/3 moderate 0/3 low risk
**T cells**	Large CD8+ T cell clones	1	124	Large CD8 clones were associated with response in 1/1 studies.	Large CD8 clones were associated with PFS in 1/1 studies.	Large CD8 clones were associated with OS in 1/1 studies.	1/1 high risk, 0/1 moderate 0/1 low risk of bias

Abbreviations; Ig: immunoglobulin, LDH: lactate dehydrogenase, SNP: single-nucleotide polymorphism.

Table S6: Summary of peripheral blood biomarkers that were studied for combination therapy and assessed risk of bias per article

**Combination therapy**	**Biomarker in blood**	**Studies**	**Patients**	**Response**	**PFS**	OS	**Quality assessment**
**Blood count /**	Relative lymphocyte count (RLC)	1	209	RLC was associated with response in 1/1 studies.		RLC was not associated with OS in 1/1 studies.	0/1 high risk, 1/1 moderate 0/1 low risk of bias

**cytology**	Absolute eosinophil count (AEC)	1	209	AEC was not associated with response in 1/1 studies.		AEC was not associated with OS in 1/1 studies.	0/1 high risk, 1/1 moderate 0/1 low risk of bias
	Relative eosinophil count (REC)	1	209	REC was associated with response in 1/1 studies.		REC was associated with OS in 1/1 studies.	0/1 high risk, 1/1 moderate 0/1 low risk of bias
	Relative basophil count (RBC)	1	209	RBC was not associated with response in 1/1 studies.		RBC was associated with OS in 1/1 studies.	0/1 high risk, 1/1 moderate 0/1 low risk of bias
	Absolute monocyte count (AMC)	1	209	AMC was not associated with response in 1/1 studies.		AMC was associated with OS in 1/1 studies.	0/1 high risk, 1/1 moderate 0/1 low risk of bias
	Neutrophil-to-lymphocyte ratio (NLR)	1	209	NLR was not associated with response in 1/1 studies.		NLR was associated with OS in 1/1 studies.	0/1 high risk, 1/1 moderate 0/1 low risk of bias
**Soluble circulating factors**	LDH	2	295	LDH was associated with response in 1/2 studies, not associated in 1/2 studies.	LDH was associated with PFS in 1/1 studies	LDH was associated with OS in 1/2 studies, not associated with OS in 1/2 studies.	1/2 high risk, 1/2 moderate, 0/2 low risk of bias
	Interleukin-8 (IL-8)	1	15	IL-8 was associated with response in 1/1 studies.		IL-8 was associated with OS in 1/1 studies.	0/1 high risk, 1/1 moderate, 0/1 low risk of bias
	S100B	1	86	S100B was associated with response in 1/1 studies.		S100B was associated with OS in 1/1 studies.	1/1 high risk, 0/1 moderate 0/1 low risk of bias
	Plasma protein signature	1	21		Plasma protein signature was not associated with PFS in 1/1 studies.	Plasma protein signature was not associated with OS in 1/1 studies.	0/1 high risk 0/1 moderate, 1/1 low risk of bias
**Tumor factors**	circulating tumor DNA (ctDNA)	1	35	ctDNA was associated with response in 1/1 studies.	ctDNA was associated with PFS in 1/1 studies.	ctDNA was associated with OS in 1/1 studies.	1/1 high risk, 0/1 moderate 0/1 low risk of bias
**T cell regulation / tumor infiltration**	CD95 on CD4+ T-cells	1	127	CD95+CD4+ cells were not associated with response in 1/1 studies.			1/1 high risk, 0/1 moderate 0/1 low risk of bias
	PD-L1 expression on CD4+ T-cells	1	127	PD-1+CD4+ cells were not associated with response in 1/1 studies.	PD-1+CD4+ cells were associated with PFS in 1/1 studies.	PD-1+CD4+ cells were not associated with OS in 1/1 studies.	1/1 high risk, 0/1 moderate 0/1 low risk of bias
	PD-L1 expression on CD8+ T-cells	1	127	PD-1+CD8+ cells were not associated with response in 1/1 studies.	PD-1+CD8+ cells were not associated with PFS in 1/1 studies.	PD-1+CD8+ cells were associated with OS in 1/1 studies.	1/1 high risk, 0/1 moderate 0/1 low risk of bias
	CD137 on CD8+ T-cells	1	127	CD137+CD8+ cells were associated with response in 1/1 studies.			1/1 high risk, 0/1 moderate 0/1 low risk of bias
	Large CD8+ T-cell clones	1	124	Large CD8 clones were associated with response in 1/1 studies.	Large CD8 clones were associated with PFS in 1/1 studies.	Large CD8 clones were associated with OS in 1/1 studies.	1/1 high risk, 0/1 moderate 0/1 low risk of bias
	T cell repertoire (TCR) diversity	1	80		TCR diversity was associated with PFS in 1/1 studies.		0/1 high risk, 1/1 moderate, 0/1 low risk of bias
**Other factors**	SNPs associated with autoimmune diseases	1	45	SNPs were not associated with response in 1/1 studies.			0/1 high risk, 1/1 moderate, 0/1 low risk of bias

Abbreviations; LDH: lactate dehydrogenase, MHC: major histocompatibility complex, SNP: single-nucleotide polymorphism.

Table S7: Summary of tumor-tissue biomarkers that were studied for anti-CLTA-4 therapy and assessed risk of bias per article.

Anti-CLTA4	Biomarker in tumour	Studies	Patients	Response	PFS	OS	Quality assessment
**Mutations / gene pathways**	BRAF mutation	6	447	BRAF mutation was not associated with response in 3/3 studies.	BRAF mutation was associated with PFS in 1/1 studies.	BRAF was associated with OS in 1/3 studies, not associated with OS in 2/3 studies.	2/6 high risk, 4/6 moderate, 0/6 low risk of bias
	NRAS mutation	2	209			NRAS was not associated with OS in 2/2 studies.	1/2 high risk, 1/2 moderate, 0/2 low risk of bias
	SERPINB3/4/9 mutation	2	216	SERPINB3/4/9 was associated with response in 1/1 studies.	SERPINB3/4/9 was associated with PFS in 2/2 studies.	SERPINB3/4/9 was associated with OS in 2/2 studies.	1/2 high risk, 1/2 moderate, 0/2 low risk of bias

	c-KIT mutation	1	97			c-KIT was not associated with OS in 1/1 studies.	0/1 high risk, 1/1 moderate, 0/1 low risk of bias
	TP53	1	110	TP53 was not associated with response in 1/1 studies.	TP53 was associated with PFS in 1/1 studies.	TP53 was associated with OS in 1/1 studies.	0/1/ high risk, 0/1 moderate, 1/1 low risk of bias
	(immune) Gene expression profiling	4	304	GEP was associated with response in 4/4 studies.			2/4 high risk, 2/4 moderate, 0/4 low risk of bias
	Memory B-cell like gene expression	1	42	Memory B-cell like genes were associated with response in 1/1 studies		Memory B-cell genes were associated with response in 1/1 studies	1/1 high risk, 0/1 moderate, 0/1 low risk of bias
	Copy number alteration (CNA)	2	230	CNA was associated with response in 1/1 studies.		CNA was associated with OS in 1/1 studies.	0/2 high risk, 2/2 moderate, 0/2 low risk of bias
	CNA of IFNg pathway	1	104	CNA of IFN pathway were associated with response in 1/1 studies.			0/1 high risk, 1/1 moderate, 0/1 low risk of bias
	IDO expression	1	64	IDO expression was associated with response in 1/1 studies.			0/1 high risk, 1/1 moderate, 0/1 low risk of bias
	DUX4 (embryonic transcription factor)	1	80	DUX4 was associated with response in 1/1 studies.	DUX4 was associated with PFS in 1/1 studies.	DUX4 was associated with OS in 1/1 studies.	1/1 high risk, 0/1 moderate, 0/1 low risk of bias
	miR222 expression	1	13	miR222 was associated with response in 1/1 studies.			1/1 high risk, 0/1 moderate, 0/1 low risk of bias
	phospho-Akt expression	1	81		p-Akt was associated with PFS in 1/1 studies.	p-Akt was associated with OS in 1/1 studies.	0/1 high risk, 1/1 moderate, 0/1 low risk of bias
**Tumor-related factors**	PD-L1 expression on tumor cells	5	637	PD-L1 was not associated with response in 5/5 studies.	PD-L1 was not associated with PFS in 1/1 studies.	PDL-1 was not associated with OS in 3/3 studies.	2/5 high risk, 3/5 moderate, 0/5 low risk of bias
	PD-L1 on mRNA level	1	111	PD-L1 on mRNA was not associated with response in 1/1 studies.			0/1 high risk, 1/1 moderate, 0/1 low risk of bias
	Tumor Mutation Burden (TMB)	7	724	TMB was associated with response in 3/6 studies, not associated in 3/6 studies.		TMB was associated with OS in 2/4 studies, not associated with OS in 2/4 studies.	3/7 high risk, 4/7 moderate, 0/7 low risk of bias
	Neoantigen load	5	385	Neoantigen load was associated with response in 2/3 studies., not associated in 1/3 studies.		Neoantigen load was associated with OS in 2/2 studies.	2/5 high risk 3/5 moderate, 0/5 low risk of bias
	MageA cancer germline antigens	1	81	MageA genes were associated with response in 1/1 studies.		MageA genes were associated with OS in 1/1 studies.	0/1 high risk, 1/1 moderate, 0/1 low risk of bias
**Tumor infiltrating cells**	TILs/ TIL score	1	64	TILs were associated with response in 1/1 studies.			0/1 high risk, 1/1 moderate, 0/1 low risk of bias
	TILs in lymph nodes	2	26	TILs in lymph nodes were associated with response in 2/2 studies.	TILs in lymph nodes were not associated with PFS in 1/1 studies.	TILs in lymph nodes were not associated with OS in 1/1 studies.	1/2 high risk, 1/2 moderate, 0/2 low risk of bias
	Total CD4+ T-cells	3	147	CD4 T cells were associated with response in 1/3 studies, not associated in 2/3 studies.		CD4 T cells were not associated with OS in 1/1 studies.	0/3 high risk, 3/3 moderate, 0/3 low risk of bias
	Total CD3+ T-cells	1	17	CD3 T cells were associated with response in 1/1 studies.			0/1 high risk, 1/1 moderate, 0/1 low risk of bias
	Total CD8+ T-cells	5	278	CD8 T cells were associated with response in 2/4 studies, not associated in 2/4 studies.		CD8 T cells were asccoiated with OS in 1/2 studies, not associated in 1/1 studies.	1/4 high risk, 3/4 moderate, 0/4 low risk of bias
	Total CD20+ B-cells	1	35	CD 20+ B cells were not associated with response in 1/1 studies.	CD 20+ B cells were not associated with PFS in 1/1 studies.		0/1 high risk, 1/1 moderate, 0/1 low risk of bias
	FOXP3 /regulatory T-cells	4	169	FOXP3 was associated with response in 2/4 studies, not associated in 2/4 studies.		FOXP3 was associated with OS in 2/2 studies.	0/4 high risk, 4/4 moderate, 0/4 low risk of bias
	Natural killer (NK) cells	1	30	NK cells were associated with response 1/1 studies.			0/1 high risk, 1/1 moderate, 0/1 low risk of bias
	CD68+ macrophages	1	30	CD68+ cells were associated with response in 1/1 studies.		CD68+ cells were not associated with OS in 1/1 studies.	0/1 high risk, 1/1 moderate, 0/1 low risk of bias

	MHC Class I expression or HLA-A	2	174	MHC I expression was associated with response in 1/2 studies, not associated in 1/2 studies.		MHC I expression was associated with OS in 1/1 studies.	0/2 high risk, 2/2 moderate, 0/2 low risk of bias
	MHC Class II expression	1	110	MHC II expression not associated with response in 1/1 studies.		MHC II expression was associated with OS in 1/1 studies.	0/1 high risk, 1/1 moderate, 0/1 low risk of bias
	Granzyme B	2	150	Granzyme B was associated with response in 1/2 studies, but not in 1/2 studies.			1/2 high risk, 1/2 moderate, 0/2 low risk of bias
	Granzyme A	1	150	Granzyme A was associated with response in 1/1 studies.			0/1 high risk, 1/1 moderate, 0/1 low risk of bias
	Perforin	2	214	Perforin was associated with response in 1/2 studies, not associated in 1/2 studies.			0/2 high risk, 2/2 moderate, 0/2 low risk of bias
	PD-1 expression on T-cells	3	222	PD-1 was not associated with response in 3/3 studies.		PD-1 was not associated with OS in 1/1 studies.	1/3 high risk, 2/3 moderate, 0/3 low risk of bias
	PD-L1 expression on CD163 macrophages	1	114			PD-L1 on macrophages were associated with OS in 1/1 studies	1/1 high risk, 0/1 moderate, 0/1 low risk of bias
	CTLA-4 expression on T-cells	4	290	CTLA-4 was associated with response in 2/3 studies, not associated in 1/3 studies.	CTLA-4 was associated with PFS in 1/1 studies.	CTLA-4 was associated with OS in 1/2 studies, not associated with OS in 1/2 studies.	1/4 high risk, 3/4 moderate, 0/4 low risk of bias
**Other factors**	SNP in association with TMB	1	51	SNP + TMB were associated with response in 1/1 studies	SNP + TMB were associated with PFS in 1/1 studies	SNP + TMB were associated with OS in 1/1 studies	1/1 high risk, 0/1 moderate, 0/1 low risk of bias
	Single nucleotide polymorphisms (SNP)	1	64	SNPs were not associated with response in 1/1 studies.			0/1 high risk, 1/1 moderate, 0/1 low risk of bias
	Tertiary lymphoid structure	1	77			TLS-high tumors were associated with OS in 1/1 studies.	0/1 high risk, 1/1 moderate, 0/1 low risk of bias

Abbreviations; IDO: Indoleamine 2,3-dioxygenase, MHC: major histocompatibility complex, TILs; tumor infiltrating lymphocytes

Table S8: Summary of tumor-tissue biomarkers that were studied for anti-PD1 therapy and assessed risk of bias per article.

**Anti-PD1**	**Biomarker in tumour**	**Studies**	**Patients**	**Response**	**PFS**	OS	**Quality assessment**
**Mutations / gene pathways**	BRAF status	6	433	BRAF was not associated with response in 3/3 studies.	BRAF was associated with PFS in 1/4 studies but with PFS in 3/4 studies.	BRAF was not associated with OS in 3/3 studies.	1/6 high risk, 4/6 moderate, 1/6 low risk of bias
	NRAS status	3	235	NRAS was associated with response in 2/3 studies, not associated in 1/3 studies.	NRAS was not associated with PFS in 1/1 studies.	NRAS was not associated with OS in 1/1 studies.	1/3 high risk, 2/3 moderate, 0/3 low risk of bias
	BRCA2 mutation	1	38	BRCA2 was associated with response in 1/1 studies.			1/1 high risk, 0/1 moderate, 0/1 low risk of bias
	cKIT status	2	222	cKIT was not associated with response in 1/1 studies.	cKIT was not associated with PFS in 2/2 studies.	cKIT was not associated with OS in 2/2 studies.	1/2 high risk, 1/2 moderate, 0/2 low risk of bias
	IRF-1 expression	1	62	IRF-1 expression was associated with response in 1/1 studies.	IRF-1 expression was associated with PFS in 1/1 studies.		0/1 high risk, 1/1 moderate, 0/1 low risk of bias
	DUX4 expression	1	80	DUX4 was not associated with response in 1/1 studies.	DUX4 was not associated with PFS in 1/1 studies.	DUX4 was not associated with OS in 1/1 studies.	1/1 high risk, 0/1 moderate, 0/1 low risk of bias
	Gene expression profiling (GEP)	9	1237	GEP was associated with response in 7/9 studies, not associated in 2/9 studies.	GEP was associated with PFS in 1/2 studies, not associated in 1/2 studies	GEP was not associated with OS in 2/2 studies.	4/9 high risk, 5/9 moderate, 0/9 low risk of bias
	RNA gene expression profiles of immune cells	1	228	RNA expression immunoscore was associated with response in 1/1 studies.	RNA expression immunoscore was not associated with PFS in 1/1 studies.	RNA expression immunoscore was not associated with OS in 1/1 studies.	1/1 high risk, 0/1 moderate, 0/1 low risk of bias
	Memory B-cell like genes	1	28	Memory B-cell genes were associated with response in 1/1 studies		Memory B-cell genes were not associated with response in 1/1 studies	1/1 high risk, 0/1 moderate, 0/1 low risk of bias
	TNFRSF9 mRNA expression	1	169	mRNA was not associated with response in 1/1 studies.	mRNA was associated with PFS in 1/1 studies.	mRNA was associated with OS in 1/1 studies.	0/1 high risk, 1/1 moderate, 0/1 low risk of bias

	TNFRSF9 DNA methylation	1	169	DNA methylation was not associated with response in 1/1 studies.	DNA methylation was associated with PFS in 1/1 studies.		0/1 high risk, 1/1 moderate, 0/1 low risk of bias
	phosphostat-1 (pSTST-1)	1	61	pSTST-1 was associated with response in 1/1 studies.			0/1 high risk, 1/1 moderate, 0/1 low risk of bias
	SERPINB3/4/9 mutation	1	68	SERPINB3/4/9 not was associated with response in 1/1 studies.			1/1 high risk, 0/1 moderate, 0/1 low risk of bias
**Tumor-related factors**	PD-L1 expression on tumor cells	12	1481	PD-L1 was associated with response in 7/12 studies, not associated in 5/12 studies.	PD-L1 was associated with PFS in 2/5 studies, not associated in 3/5 studies.	PD-L1 was associated with OS in 3/4 studies, not associated in 1/4 studies.	8/12 high risk, 4/12 moderate, 0/12 low risk of bias
	PD-1/PD-L1 interactions	1	166	PD1/PD-L1 was associated with response in 1/1 studies.	PD1/PD-L1 was associated with PFS in 1/1 studies.	PD1/PD-L1 was associated with OS in 1/1 studies.	0/1 high risk, 1/1 moderate, 0/1 low risk of bias
	Tumor Mutational Burden (TMB)	8	438	TMB was associated with response in 3/6 studies, not associated in 3/6 studies	TMB was associated with PFS in 2/2 studies.	TMB was associated with OS in 3/4 studies, not associated with OS in 1/4 studies.	4/8 high risk, 4/8 moderate, 0/8 low risk of bias
	Neoantigen load (NAL)	1	68	NAL was associated with response in 1/1 studies.			1/1 high risk, 0/1 moderate, 0/1 low risk of bias
	Copy number alteration (CNA)	2	116	CNA was associated with response in 1/2 studies, not associated in 1/2 studies.			1/2 high risk, 1/2 moderate, 0/2 low risk of bias
	MageA cancer germline antigens	1	63	MageA genes were not associated with response in 1/1 studies.		MageA genes were not associated with OS in 1/1 studies.	0/1 high risk, 1/1 moderate, 0/1 low risk of bias
**Tumor infiltrating cells**	Tumor Infiltrating Lymphocytes (TILs)	2	121	TILs were associated with response in 2/2 studies.			2/2 high risk, 0/2 moderate, 0/2 low risk of bias
	Total CD4+ T-cells	6	292	CD4 cells were associated with response in 3/6 studies, not in 3/6 studies.	CD4 T cells were not associated with PFS in 1/1 studies.	CD4 T cells were not associated with OS in 1/1 studies.	3/6 high risk, 2/6 moderate, 1/6 low risk of bias
	PD1+ T-cells	1	23	PD1+T cells were associated with response in 1/1 studies.	PD1+T cells were associated with PFS in 1/1 studies.	PD1+T cells were associated with OS in 1/1 studies.	1/1 high risk, 0/1 moderate, 0/1 low risk of bias
	Total CD8+ T-cells	7	315	CD8 cells were associated with response in 4/7 studies, not associated in 3/7 studies.	CD8 cells were associated with PFS in 1/2 studies, not associated in 1/2 studies.	CD8 cells were associated with OS in 2/2 studies.	4/7 high risk, 2/7 moderate, 1/7 low risk of bias
	Tumor-resistant CD8+ T-cells	1	13	TR-CD8 cells were not associated with response in 1/1 studies.	TR- CD8 T cells were associated with MSS in 1/1 studies.		1/1 high risk, 0/1 moderate, 0/1 low risk of bias
	CD8+ memory T-cells	1	53	CD8 memory T cells were associated with response in 1/1 studies,			1/1 high risk, 0/1 moderate, 0/1 low risk of bias
	CD163+ cells (macrophages)	2	93	CD163+ cells were associated with response in 2/2 studies.	CD163+ cells were associated with PFS in 1/1 studies.	CD163+ cells were not associated with OS in 1/1 studies.	2/2 high risk, 0/2 moderate, 0/2 low risk of bias
	CD8+/CD4+ ratio	2	43	CD8+CD4+ ratio was associated with response in 2/2 studies.			2/2 high risk, 0/2 moderate, 0/2 low risk of bias
	Total CD3+ T-cells	3	170	CD3 cells were associated with response in 2/3 studies, not associated in 1/3 studies.	CD3 T cells were not associated with PFS in 1/1 studies.	CD3 T cells were associated with OS in 1/1 studies.	1/3 high risk, 1/3 moderate, 1/3 low risk of bias
	CD4+ effector memory cells	2	73	CD4+ memory cells were associated with response in 2/2 studies.			2/2 high risk, 0/2 moderate, 0/2 low risk of bias
	CD 20+ T-cells (Th1/Tc1-like)	2	117	CD 20 was not associated with response in 2/2 studies.		CD 20 T cells were not associated with OS in 1/1 studies.	1/2 high risk, 0/2 moderate, 1/2 low risk of bias
	CTLA-4+PD-1+ CD8+ TIL's	1	40	CTLA-4+PD-1+CD8+TILs were associated with response in 1/1 studies.	CTLA-4+PD-1+CD8+TILs were associated with PFS in 1/1 studies.	CTLA-4+PD-1+CD8+TILs were not associated with OS in 1/1 studies.	1/1 high risk, 0/1 moderate, 0/1 low risk of bias
	Regulatory T-cells	1	53	Tregs were not associated with response in 1/1 studies.			1/1 high risk, 0/1 moderate, 0/1 low risk of bias
	B-cells	1	53	B cells were not associated with response in 1/1 studies.			1/1 high risk, 0/1 moderate, 0/1 low risk of bias
	IDO-1/HLA-DR+ interaction	1	166	IDO-1/HLA-DR was associated with response in 1/1 studies.	IDO-1/HLA-DR was associated with PFS in 1/1 studies.	IDO-1/HLA-DR was associated with OS in 1/1 studies.	0/1 high risk, 1/1 moderate, 0/1 low risk of bias

	HLA-A (MHC Class I)	4	212	HLA-A was associated with response in 1/4 studies, not associated in 3/4 studies.		HLA-A was not associated with OS in 1/1 studies.	3/4 high risk, 1/4 moderate, 0/4 low risk of bias
	MHC Class II expression	3	201	MHC II was associated with response in 2/3 studies, not associated in 1/3 studies.	MHC Class II expression was associated with PFS in 1/1 studies.	MHC Class II expression was associated with OS in 1/1 studies.	2/3 high risk, 1/3 moderate, 0/3 low risk of bias
	LAG3 on T-cells	1	53	LAG3 was associated with response in 1/1 studies.			0/1 high risk, 1/1 moderate, 0/1 low risk of bias
	FOXP3 (Tregs)	2	66	FOXP3 was associated with response in 1/2 studies, not associated in 1/2 studies.			1/2 high risk, 1/2 moderate, 0/2 low risk of bias
	Granzyme A	1	35	Granzyme A was associated with response in 1/1 studies.			1/1 high risk, 0/1 moderate, 0/1 low risk of bias
	Granzyme B	3	223	Granzyme B was associated with response in 2/3 studies, not associated in 1/3 studies.			1/3 high risk, 2/3 moderate, 0/3 low risk of bias
	T Cell Receptor (TCR) repertoire	4	184	TCR diversity was associated with response in 3/4 studies, not associated in 1/4 studies.			2/4 high risk, 2/4 moderate, 0/4 low risk of bias
	Tumor score based on predicted MHC	1	167			Tumour fitness score was associated with OS in 1/1 studies.	0/1 high risk, 0/1 moderate, 1/1 low risk of bias
	PD-1 expression on T cells	3	142	PD-1 was associated with response in 2/3 studies, not associated in 1/3 studies.		PD-1 expression was not associated with OS in 1/ 1studies.	1/3 high risk, 2/3 moderate, 0/3 low risk of bias
	PD-L1 expression on macrophages	1	23	PD-L1 on macrophages during treatment was associated with response in 1/1 studies.			1/1 high risk, 0/1 moderate, 0/1 low risk of bias
	CTLA-4 expression on T cells	2	48	CTLA-4 was associated with response in 1/2 studies, not associated in 1/2 studies.		CTLA-4 was not associated with OS in 1/1 studies.	2/2 high risk, 0/2 moderate, 0/2 low risk of bias
**Other**	Tertiary lymphoid structure	1	69			TLS-high tumors were associated with OS in 1/1 studies.	0/1 high risk, 1/1 moderate, 0/1 low risk of bias

Abbreviations; IRF: interferon regulatory factor, MHC: major histocompatibility complex, TILs; tumor infiltrating lymphocytes.

Table S9: Summary of tumor-tissue biomarkers that were studied for mixed therapy cohorts and assessed risk of bias per article.

**Mixed cohorts**	**Biomarker in tumour**	**Studies**	**Patients**	**Response**	**PFS**	OS	**Quality assessment**
**Mutations / gene pathways**	NRAS mutation	2	444	NRAS was not associated with response in 1/1 studies.	NRAS was not associated with PFS in 2/2 studies.	NRAS was associated with OS in 1/1 studies.	0/2 high risk, 2/2 moderate, 0/2 low risk of bias
	BRAF mutation	2	112		BRAF was not associated with PFS in 2/2 studies	BRAF was not associated with OS in 1/1 studies	1/2 high risk, 1/2 moderate, 0/2 low risk of bias
	LPR1B mutation	1	332		LPR1B mutation was associated with MSS in 1/1 studies.		0/1 high risk, 1/1 moderate, 0/1 low risk of bias
	P/LP germline	1	59	P/LP germline was associated with response in 1/1 studies.	P/LP germline was associated with PFS in 1/1 studies.		0/1 high risk, 1/1 moderate, 0/1 low risk of bias
	Tumor transcriptomics	2	118	Tumor transcriptomics were not associated with response in 1/1 studies.	Tumor transcriptomics were associated with PFS in 1/1 studies.	Tumor transcriptomics were associated with OS in 1/2 studies, not associated in 1/2 studies.	1/2 high risk, 1/2 moderate, 0/2 low risk of bias
	PSMB8 and PSMB9 gene expression	1	46	PSMB8/9 genes were associated with response in 1/1 studies.			1/1 high risk, 0/1 moderate, 0/1 low risk of bias
	PD-L1 expression on tumor cells	5	298	PD-L1 expression was associated with response in 2/3 studies, not associated in 1/3 studies.	PD-L1 expression was not associated with PFS in 1/1 studies.	PD-L1 expression was associated with OS in 1/4 studies, not associated in 3/4 studies.	2/5 high risk 3/5 moderate, 0/5 low risk of bias

**Tumor-related factors**	Tumor mutational burden (TMB)	5	861	TMB was associated with response in 4/5 studies, not associated in 1/5 studies.	TMB was associated with PFS in 1/2 studies, but not associated with PFS in 1/2 studies.	TMB was associated with OS in 1/4 studies, not associated with OS in 3/4 studies.	2/5 high risk, 3/5 moderate, 0/5 low risk of bias
	Neoantigen load (NAL)	2	423	NAL was associated with response in 1/2 studies, not associated in 1/2 studies.		NAL was associated with OS in 1/2 studies, not associated with OS in 1/2 studies.	1/2 high risk, 1/2 moderate, 0/2 low risk of bias
	Neoepitope burden	1	302	Neoepitope burden was not associated with response in 1/1 studies.		Neoepitope burden was not associated with OS in 1/1 studies.	0/1 high risk 1/1 moderate, 0/1 low risk of bias
	Neopeptide DIA	1	131			Neoantigen DIA was not associated with OS in 1/1 studies	0/1 high risk 1/1 moderate, 0/1 low risk of bias
	Fibroblast activation protein (FAP)	1	117	FAP was not associated with response in 1/1 studies.	FAP was associated with PFS in 1/1 studies.	FAP was associated with OS in 1/1 studies.	0/1 high risk, 0/1 moderate, 1/1 low risk of bias
	Cancer associated fibroblasts: Thy1 and smooth muscle actin	1	117	Thy1 and SMA were not associated with response in 1/1 studies.	Thy1 and SMA were not associated with PFS in 1/1 studies.	Thy1 and SMA were not associated with OS in 1/1 studies.	0/1 high risk, 0/1 moderate, 1/1 low risk of bias
**Tumor infiltrating cells**	TILs	2	123	TILs were associated with response in 1/1 studies.	TILs were not associated with PFS in 1/1 studies.	TILs were associated with OS in 2/2 studies.	1/2 high risk, 1/2 moderate, 0/2 low risk of bias
	TIL clonality	1	91	TIL clonality was associated with response in 1/1 studies.		TIL clonality was not associated with OS in 1/1 studies.	0/1 high risk 1/1 moderate, 0/1 low risk of bias
	FOXP3/Tregs	1	32			Tregs were not associated with OS in 1/1 studies.	1/1 high risk, 0/1 moderate, 0/1 low risk of bias
	CD8+TIL status	3	160			CD8+TIL was associated with OS in 1/3 studies, not associated in 2/3 studies.	2/3 high risk, 1/3 moderate, 0/3 low risk of bias
	CD4+ T cells	1	32			CD4+ cells were not associated with OS in 1/1 studies.	1/1 high risk, 0/1 moderate, 0/1 low risk of bias
	PD-L1+/CD8+TIL s	1	51			PD-L1+/CD8+TIL status was associated with OS in 1/1 studies.	0/1 high risk 1/1 moderate, 0/1 low risk of bias
	HLA-B44 supertype	1	269			HLA-B44 was associated with OS in 1/1 studies.	0/1 high risk 1/1 moderate, 0/1 low risk of bias
	HLA-B62 supertype	1	269			HLA-B62 was associated with OS in 1/1 studies.	0/1 high risk 1/1 moderate, 0/1 low risk of bias
	MHC Class I expression	1	110	MHC Class I expression was not associated in 1/1 studies.		MHC Class I expression was not associated with OS in 1/1 studies.	0/1 high risk 1/1 moderate, 0/1 low risk of bias
	MHC Class II expression	1	110	MHC Class II expression was not associated in 1/1 studies.		MHC Class II expression was not associated with OS in 1/1 studies.	0/1 high risk 1/1 moderate, 0/1 low risk of bias
	PD-1 expression on T cells	1	32			PD-1 was not associated with OS in 1/1 studies.	1/1 high risk, 0/1 moderate, 0/1 low risk of bias
	CTLA-4 expression on T cells	1	32			CTLA-4 was not associated with OS in 1/1 studies.	1/1 high risk, 0/1 moderate, 0/1 low risk of bias

Abbreviations; DIA; differential agretopicity index, MHC: major histocompatibility complex, LRR1B: lipoprotein receptor-related 1B, P/LP: pathogenic/likely pathogenic, TILs; tumor infiltrating lymphocytes.

Table S10: Summary of tumor-tissue biomarkers that were studied for combination therapy and assessed risk of bias per article.

**Combination**	**Biomarker in tumour**	**Studies**	**Patients**	**Response**	**PFS**	OS	**Quality assessment**
**Gene pathways**	T-cell inflamed GEP	1	57	GEP was associated with response in 1/1 studies.			0/1 high risk 1/1 moderate, 0/1 low risk of bias

	IRF-1 expression	1	47	IRF-1 expression was associated with response in 1/1 studies.	IRF-1 expression was associated with PFS in 1/1 studies.		0/1 high risk, 1/1 moderate, 0/1 low risk of bias
**Tumor-related factors**	Tumor mutational burden (TMB)	1	35	TMB was associated with response in 1/1 studies.		TMB was not associated with OS in 1/1 studies.	1/1 high risk, 0/1 moderate, 0/1 low risk of bias
**Tumor infiltrating cells**	Memory T cells	1	57		Memory T cells were associated with PFS in 1/1 studies.		0/1 high risk 1/1 moderate, 0/1 low risk of bias
	CD8+T cells	1	57	CD8+ T cells were associated with response in 1/1 studies.		CD8+TIL was associated with OS in 1/3 studies, not associated in 2/3 studies.	0/1 high risk 1/1 moderate, 0/1 low risk of bias
	CTLA-4 promotor methylation	1	50	mCTLA4 was associated with response in 1/1 studies.		mCTLA4 was associated with OS in 1/1 studies.	0/1 high risk 1/1 moderate, 0/1 low risk of bias

Abbreviations: IRF-1: interferon regulatory factor 1.

Table S11: Summary of fecal biomarkers that were studied for all ICIs and assessed risk of bias per article.

**Biomarker**	**Number of studies**	**Number of patients**	**Response**	**Survival**	**Quality assessment**
**Gut microbiomes**	2 for anti-PD-1, 2 for mixed cohorts	104 for anti-PD-1, 66 for mixed cohorts	Gut microbiomes were associated with response in 4/4 studies.	Gut microbiomes were associated with PFS in 1/1 studies.	2/3 high risk, 1/3 moderate, 0/3 low risk of bias
**Gut metabolics**	1 for anti-PD-1, 1 for mixed cohorts	14 for anti-PD-1, 24 for mixed cohorts	Gut metabolics were associated with response in 2/2 studies.		2/2 high risk, 0/2 moderate, 0/2 low risk of bias
**Oral microbiomes**	1 for anti-PD-1	89 for anti-PD-1	Oral microbiomes were not associated with response in 1/1 studies.		0/1 high risk 1/1 moderate, 0/1 low risk of bias

Complete search strategy for systematic review

Date searches run (all databases search from inception to this date): 6/18/2020

Pubmed search

- *Strategy 1*

(("Melanoma"[Mesh] OR "melanoma"[tw] OR "melanoma*"[tw] OR "melanotic Freckle*"[tw])

AND

("Antineoplastic Agents, Immunological"[Mesh] OR "Antineoplastic Agents, Immunological"[Pharmacological Action] OR "Immunotherapy"[Mesh:noexp] OR "immunotherap*"[tw] OR "immune therap*"[tw] OR "abagovomab"[tw] OR "Ado-Trastuzumab Emtansine"[tw] OR "Alemtuzumab"[tw] OR "bavituximab"[tw] OR "Bevacizumab"[tw] OR "BI 836826"[tw] OR "bivatuzumab mertansine"[tw] OR "BMS-936559"[tw] OR "Brentuximab Vedotin"[tw] OR "cantuzumab mertansine"[tw] OR "cemiplimab"[tw] OR "Cetuximab"[tw] OR "conatumumab"[tw] OR "dacetuzumab"[tw] OR "DS-8273a"[tw] OR "durvalumab"[tw] OR "ecromeximab"[tw] OR "epratuzumab"[tw] OR "Gemtuzumab"[tw] OR "glembatumumab vedotin"[tw] OR "Inotuzumab Ozogamicin"[tw] OR "intetumumab"[tw] OR "Ipilimumab"[tw] OR "labetuzumab"[tw] OR "lintuzumab"[tw] OR "lorvotuzumab mertansine"[tw] OR "magrolimab"[tw] OR "necitumumab"[tw] OR "nimotuzumab"[tw] OR "Nivolumab"[tw] OR "obinutuzumab"[tw] OR "oregovomab"[tw] OR "Panitumumab"[tw] OR "pembrolizumab"[tw] OR "pertuzumab"[tw] OR "PF-06263507"[tw] OR "pidilizumab"[tw] OR "rilotumumab"[tw] OR "rindopepimut"[tw] OR "Rituximab"[tw] OR "talimogene laherparepvec"[tw] OR "Trastuzumab"[tw] OR "zalutumumab"[tw] OR "zanolimumab"[tw] OR "Immune checkpoint block*"[tw])

AND

("response"[tw] OR "respon*"[tw] OR "Treatment Outcome"[Mesh] OR "outcom*"[tw] OR "Disease-Free Survival"[tw] OR "Progression-Free Survival"[tw] OR "Treatment Failure"[tw]) AND ("Biomarkers"[Mesh] OR "biomarkers"[tw] OR "biomarker"[tw] OR "biomarker*"[tw] OR "Biochemical Marker"[tw] OR "Biochemical Markers"[tw] OR "Biologic Marker"[tw] OR "Biologic Markers"[tw] OR "Biological Marker"[tw] OR "Biological Markers"[tw] OR "Clinical Marker"[tw] OR "Clinical Markers"[tw] OR "Immune Marker"[tw] OR "Immune Markers"[tw] OR "Immunologic Marker"[tw] OR "Immunologic Markers"[tw] OR "Laboratory Marker"[tw] OR "Laboratory Markers"[tw] OR "Serum Marker"[tw] OR "Serum Markers"[tw] OR "Surrogate End Point"[tw] OR "Surrogate End Points"[tw] OR "Surrogate Endpoint"[tw] OR "Surrogate Endpoints"[tw] OR "Surrogate Marker"[tw] OR "Surrogate Markers"[tw] OR "Viral Marker"[tw] OR "Viral Markers"[tw] OR "marker"[tw] OR "markers"[tw])

AND

("predictor"[tw] OR "predictors"[tw] OR "predictor*"[tw] OR "Survival Analysis"[Mesh:noexp] OR "Kaplan-Meier Estimate"[mesh] OR "Proportional Hazards Models"[mesh] OR "Probability"[Mesh]))

- *Strategy 2*

(("Melanoma"[majr] OR "melanoma"[ti] OR "melanoma*"[ti] OR "melanotic Freckle*"[ti])

AND

("Antineoplastic Agents, Immunological"[majr] OR "Immunotherapy"[majr:noexp] OR "immunotherap*"[ti] OR "immune therap*"[ti] OR "abagovomab"[ti] OR "Ado-Trastuzumab Emtansine"[ti] OR "Alemtuzumab"[ti] OR "bavituximab"[ti] OR "Bevacizumab"[ti] OR "BI 836826"[ti] OR "bivatuzumab mertansine"[ti] OR "BMS-936559"[ti] OR "Brentuximab Vedotin"[ti] OR "cantuzumab mertansine"[ti] OR "cemiplimab"[ti] OR "Cetuximab"[ti] OR "conatumumab"[ti] OR "dacetuzumab"[ti] OR "DS-8273a"[ti] OR "durvalumab"[ti] OR "ecromeximab"[ti] OR "epratuzumab"[ti] OR "Gemtuzumab"[ti] OR "glembatumumab vedotin"[ti] OR "Inotuzumab Ozogamicin"[ti] OR "intetumumab"[ti] OR "Ipilimumab"[ti] OR "labetuzumab"[ti] OR "lintuzumab"[ti] OR "lorvotuzumab mertansine"[ti] OR "magrolimab"[ti] OR "necitumumab"[ti] OR "nimotuzumab"[ti] OR "Nivolumab"[ti] OR "obinutuzumab"[ti] OR "oregovomab"[ti] OR "Panitumumab"[ti] OR "pembrolizumab"[ti] OR "pertuzumab"[ti] OR "PF-06263507"[ti] OR "pidilizumab"[ti] OR "rilotumumab"[ti] OR "rindopepimut"[ti] OR "Rituximab"[ti] OR "talimogene laherparepvec"[ti] OR "Trastuzumab"[ti] OR "zalutumumab"[ti] OR "zanolimumab"[ti] OR "Immune checkpoint block*"[ti])

AND

("response"[tw] OR "respon*"[tw] OR "Treatment Outcome"[Mesh] OR "outcom*"[tw] OR "Disease-Free Survival"[tw] OR "Progression-Free Survival"[tw] OR "Treatment Failure"[tw]) AND ("Biomarkers"[Mesh] OR "biomarkers"[tw] OR "biomarker"[tw] OR "biomarker*"[tw] OR "Biochemical Marker"[tw] OR "Biochemical Markers"[tw] OR "Biologic Marker"[tw] OR "Biologic Markers"[tw] OR "Biological Marker"[tw] OR "Biological Markers"[tw] OR "Clinical Marker"[tw] OR "Clinical Markers"[tw] OR "Immune Marker"[tw] OR "Immune Markers"[tw] OR "Immunologic Marker"[tw] OR "Immunologic Markers"[tw] OR "Laboratory Marker"[tw] OR "Laboratory Markers"[tw] OR "Serum Marker"[tw] OR "Serum Markers"[tw] OR "Surrogate End Point"[tw] OR "Surrogate End Points"[tw] OR "Surrogate Endpoint"[tw] OR "Surrogate Endpoints"[tw] OR "Surrogate Marker"[tw] OR "Surrogate Markers"[tw] OR "Viral Marker"[tw] OR "Viral Markers"[tw] OR "marker"[tw] OR "markers"[tw]))

AND

("xxxx/01/01"[PDAT] : "3000/12/31"[PDAT])

Reference list

[1] Amaral T, Schulze M, Sinnberg T, Nieser M, Martus P, Battke F, et al. Are Pathogenic Germline Variants in Metastatic Melanoma Associated with Resistance to Combined Immunotherapy? Cancers (Basel). 2020;12.
[2] Arce Vargas F, Furness AJS, Litchfield K, Joshi K, Rosenthal R, Ghorani E, et al. Fc Effector Function Contributes to the Activity of Human Anti-CTLA-4 Antibodies. Cancer Cell. 2018;33:649-63.e4.
[3] Ascierto PA, Capone M, Grimaldi AM, Mallardo D, Simeone E, Madonna G, et al. Proteomic test for anti-PD-1 checkpoint blockade treatment of metastatic melanoma with and without BRAF mutations. J Immunother Cancer. 2019;7:91.
[4] Auslander N, Zhang G, Lee JS, Frederick DT, Miao B, Moll T, et al. Robust prediction of response to immune checkpoint blockade therapy in metastatic melanoma. Nat Med. 2018;24:1545-9.
[5] Ayers M, Lunceford J, Nebozhyn M, Murphy E, Loboda A, Kaufman DR, et al. IFN-γ-related mRNA profile predicts clinical response to PD-1 blockade. J Clin Invest. 2017;127:2930-40.
[6] Babačić H, Lehtiö J, Pico de Coaña Y, Pernemalm M, Eriksson H. In-depth plasma proteomics reveals increase in circulating PD-1 during anti-PD-1 immunotherapy in patients with metastatic cutaneous melanoma. J Immunother Cancer. 2020;8.
[7] Balatoni T, Ladányi A, Fröhlich G, Czirbesz K, Kovács P, Pánczél G, et al. Biomarkers Associated with Clinical Outcome of Advanced Melanoma Patients Treated with Ipilimumab. Pathol Oncol Res. 2020;26:317-25.
[8] Balatoni T, Mohos A, Papp E, Sebestyén T, Liszkay G, Oláh J, et al. Tumor-infiltrating immune cells as potential biomarkers predicting response to treatment and survival in patients with metastatic melanoma receiving ipilimumab therapy. Cancer Immunol Immunother. 2018;67:141-51.
[9] Barak V, Leibovici V, Peretz T, Kalichman I, Lotem M, Merims S. Assessing Response to New Treatments and Prognosis in Melanoma Patients, by the Biomarker S-100β. Anticancer Res. 2015;35:6755-60.
[10] Bartlett EK, Flynn JR, Panageas KS, Ferraro RA, Sta Cruz JM, Postow MA, et al. High neutrophil-to-lymphocyte ratio (NLR) is associated with treatment failure and death in patients who have melanoma treated with PD-1 inhibitor monotherapy. Cancer. 2020;126:76-85.
[11] Bence C, Hofman V, Chamorey E, Long-Mira E, Lassalle S, Albertini AF, et al. Association of combined PD-L1 expression and tumour-infiltrating lymphocyte features with survival and treatment outcomes in patients with metastatic melanoma. J Eur Acad Dermatol Venereol. 2020;34:984-94.
[12] Bjoern J, Juul Nitschke N, Zeeberg Iversen T, Schmidt H, Fode K, Svane IM. Immunological correlates of treatment and response in stage IV malignant melanoma patients treated with Ipilimumab. Oncoimmunology. 2016;5:e1100788.
[13] Bochem J, Zelba H, Amaral T, Spreuer J, Soffel D, Eigentler T, et al. Peripheral PD-1+CD56+ T-cell frequencies correlate with outcome in stage IV melanoma under PD-1 blockade. PLoS One. 2019;14:e0221301.
[14] Brüggemann C, Kirchberger MC, Goldinger SM, Weide B, Konrad A, Erdmann M, et al. Predictive value of PD-L1 based on mRNA level in the treatment of stage IV melanoma with ipilimumab. J Cancer Res Clin Oncol. 2017;143:1977-84.
[15] Cabrita R, Lauss M, Sanna A, Donia M, Skaarup Larsen M, Mitra S, et al. Tertiary lymphoid structures improve immunotherapy and survival in melanoma. Nature. 2020;577:561-5.
[16] Campesato LF, Barroso-Sousa R, Jimenez L, Correa BR, Sabbaga J, Hoff PM, et al. Comprehensive cancer-gene panels can be used to estimate mutational load and predict clinical benefit to PD-1 blockade in clinical practice. Oncotarget. 2015;6:34221-7.
[17] Capone M, Giannarelli D, Mallardo D, Madonna G, Festino L, Grimaldi AM, et al. Baseline neutrophil-to-lymphocyte ratio (NLR) and derived NLR could predict overall survival in patients with advanced melanoma treated with nivolumab. J Immunother Cancer. 2018;6:74.
[18] Capone M, Fratangelo F, Giannarelli D, Sorrentino C, Turiello R, Zanotta S, et al. Frequency of circulating CD8+CD73+T cells is associated with survival in nivolumab-treated melanoma patients. J Transl Med. 2020;18:121.
[19] Carlino MS, Long GV, Schadendorf D, Robert C, Ribas A, Richtig E, et al. Outcomes by line of therapy and programmed death ligand 1 expression in patients with advanced melanoma treated with pembrolizumab or ipilimumab in KEYNOTE-006: A randomised clinical trial. Eur J Cancer. 2018;101:236-43.

[20] Cassidy MR, Wolchok RE, Zheng JT, Panageas KS, Wolchok JD, Coit D, et al. Neutrophil to Lymphocyte Ratio is Associated With Outcome During Ipilimumab Treatment. Ebiomedicine. 2017;18:56-61.
[21] Chakravarti N, Ivan D, Trinh VA, Glitza IC, Curry JL, Torres-Cabala C, et al. High cytotoxic T-lymphocyte-associated antigen 4 and phospho-Akt expression in tumor samples predicts poor clinical outcomes in ipilimumab-treated melanoma patients. Melanoma Res. 2017;27:24-31.
[22] Chasseuil E, Saint-Jean M, Chasseuil H, Peuvrel L, Quéreux G, Nguyen JM, et al. Blood Predictive Biomarkers for Nivolumab in Advanced Melanoma. Acta Derm Venereol. 2018;98:406-10.
[23] Chat V, Ferguson R, Simpson D, Kazlow E, Lax R, Moran U, et al. Autoimmune genetic risk variants as germline biomarkers of response to melanoma immune-checkpoint inhibition. Cancer Immunol Immunother. 2019;68:897-905.
[24] Chen G, Huang AC, Zhang W, Zhang G, Wu M, Xu W, et al. Exosomal PD-L1 contributes to immunosuppression and is associated with anti-PD-1 response. Nature. 2018;560:382-6.
[25] Chen H, Chong W, Wu Q, Yao Y, Mao M, Wang X. Association of LRP1B Mutation With Tumor Mutation Burden and Outcomes in Melanoma and Non-small Cell Lung Cancer Patients Treated With Immune Check-Point Blockades. Front Immunol. 2019;10:1113.
[26] Chen H, Yang M, Wang Q, Song F, Li X, Chen K. The new identified biomarkers determine sensitivity to immune check-point blockade therapies in melanoma. Oncoimmunology. 2019;8:1608132.
[27] Chen PL, Roh W, Reuben A, Cooper ZA, Spencer CN, Prieto PA, et al. Analysis of immune signatures in longitudinal tumor samples yields insight into biomarkers of response and mechanisms of resistance to immune checkpoint blockade. Cancer Discovery. 2016;6:827-37.
[28] Chew GL, Campbell AE, De Neef E, Sutliff NA, Shadle SC, Tapscott SJ, et al. DUX4 Suppresses MHC Class I to Promote Cancer Immune Evasion and Resistance to Checkpoint Blockade. Dev Cell. 2019;50:658-71.e7.
[29] Cho J, Ahn S, Yoo KH, Kim JH, Choi SH, Jang KT, et al. Treatment outcome of PD-1 immune checkpoint inhibitor in Asian metastatic melanoma patients: correlative analysis with PD-L1 immunohistochemistry. Invest New Drugs. 2016;34:677-84.
[30] Chow MT, Ozga AJ, Servis RL, Frederick DT, Lo JA, Fisher DE, et al. Intratumoral Activity of the CXCR3 Chemokine System Is Required for the Efficacy of Anti-PD-1 Therapy. Immunity. 2019;50:1498-512.e5.
[31] Chowell D, Morris LGT, Grigg CM, Weber JK, Samstein RM, Makarov V, et al. Patient HLA class I genotype influences cancer response to checkpoint blockade immunotherapy. Science. 2018;359:582-7.
[32] Cordonnier M, Nardin C, Chanteloup G, Derangere V, Algros MP, Arnould L, et al. Tracking the evolution of circulating exosomal-PD-L1 to monitor melanoma patients. J Extracell Vesicles. 2020;9:1710899.
[33] Cristescu R, Mogg R, Ayers M, Albright A, Murphy E, Yearley J, et al. Pan-tumor genomic biomarkers for PD-1 checkpoint blockade-based immunotherapy. Science. 2018;362:197-+.
[34] Daud AI, Loo K, Pauli ML, Sanchez-Rodriguez R, Sandoval PM, Taravati K, et al. Tumor immune profiling predicts response to anti-PD-1 therapy in human melanoma. J Clin Invest. 2016;126:3447-52.
[35] Daud AI, Wolchok JD, Robert C, Hwu WJ, Weber JS, Ribas A, et al. Programmed Death-Ligand 1 Expression and Response to the Anti-Programmed Death 1 Antibody Pembrolizumab in Melanoma. J Clin Oncol. 2016;34:4102-9.
[36] de Coana YP, Wolodarski M, Poschke I, Yoshimoto Y, Yang Y, Nystrom M, et al. Ipilimumab treatment decreases monocytic MDSCs and increases CD8 effector memory T cells in long-term survivors with advanced melanoma. Oncotarget. 2017;8:21539-53.
[37] Del Re M, Marconcini R, Pasquini G, Rofi E, Vivaldi C, Bloise F, et al. PD-L1 mRNA expression in plasma-derived exosomes is associated with response to anti-PD-1 antibodies in melanoma and NSCLC. Br J Cancer. 2018;118:820-4.
[38] Delyon J, Mateus C, Lefeuvre D, Lanoy E, Zitvogel L, Chaput N, et al. Experience in daily practice with ipilimumab for the treatment of patients with metastatic melanoma: an early increase in lymphocyte and eosinophil counts is associated with improved survival. Ann Oncol. 2013;24:1697-703.
[39] Di Giacomo AM, Calabro L, Danielli R, Fonsatti E, Bertocci E, Pesce I, et al. Long-term survival and immunological parameters in metastatic melanoma patients who responded to ipilimumab 10 mg/kg within an expanded access programme. Cancer Immunology, Immunotherapy. 2013;62:1021-8.
[40] Diem S, Hasan Ali O, Ackermann CJ, Bomze D, Koelzer VH, Jochum W, et al. Tumor infiltrating lymphocytes in lymph node metastases of stage III melanoma correspond to response and survival in nine patients treated with ipilimumab at the time of stage IV disease. Cancer Immunol Immunother. 2018;67:39-45.
[41] Diem S, Kasenda B, Martin-Liberal J, Lee A, Chauhan D, Gore M, et al. Prognostic score for patients with advanced melanoma treated with ipilimumab. Eur J Cancer. 2015;51:2785-91.
[42] Diem S, Kasenda B, Spain L, Martin-Liberal J, Marconcini R, Gore M, et al. Serum lactate dehydrogenase as an early marker for outcome in patients treated with anti-PD-1 therapy in metastatic melanoma. Br J Cancer. 2016;114:256-61.

[43] Diem S, Fässler M, Bomze D, Ali OH, Berner F, Niederer R, et al. Immunoglobulin G and Subclasses as Potential Biomarkers in Metastatic Melanoma Patients Starting Checkpoint Inhibitor Treatment. J Immunother. 2019;42:89-93.
[44] Dupuis F, Lamant L, Gerard E, Torossian N, Chaltiel L, Filleron T, et al. Clinical, histological and molecular predictors of metastatic melanoma responses to anti-PD-1 immunotherapy. Br J Cancer. 2018;119:193-9.
[45] Edwards J, Wilmott JS, Madore J, Gide TN, Quek C, Tasker A, et al. CD103(+) Tumor-Resident CD8(+) T Cells Are Associated with Improved Survival in Immunotherapy-Naïve Melanoma Patients and Expand Significantly During Anti-PD-1 Treatment. Clin Cancer Res. 2018;24:3036-45.
[46] Failing JJ, Yan Y, Porrata LF, Markovic SN. Lymphocyte-to-monocyte ratio is associated with survival in pembrolizumab-treated metastatic melanoma patients. Melanoma Res. 2017;27:596-600.
[47] Fairfax BP, Taylor CA, Watson RA, Nassiri I, Danielli S, Fang H, et al. Peripheral CD8(+) T cell characteristics associated with durable responses to immune checkpoint blockade in patients with metastatic melanoma. Nat Med. 2020;26:193-9.
[48] Fässler M, Diem S, Mangana J, Hasan Ali O, Berner F, Bomze D, et al. Antibodies as biomarker candidates for response and survival to checkpoint inhibitors in melanoma patients. J Immunother Cancer. 2019;7:50.
[49] Felix J, Cassinat B, Porcher R, Schlageter MH, Maubec E, Pages C, et al. Relevance of serum biomarkers associated with melanoma during follow-up of anti-CTLA-4 immunotherapy. Int Immunopharmacol. 2016;40:466-73.
[50] Felix J, Lambert J, Roelens M, Maubec E, Guermouche H, Pages C, et al. Ipilimumab reshapes T cell memory subsets in melanoma patients with clinical response. Oncoimmunology. 2016;5:1136045.
[51] Ferrucci PF, Ascierto PA, Pigozzo J, Del Vecchio M, Maio M, Antonini Cappellini GC, et al. Baseline neutrophils and derived neutrophilto-lymphocyte ratio: Prognostic relevance in metastatic melanoma patients receiving ipilimumab. Annals of Oncology. 2016;27:732-8.
[52] Ferrucci PF, Gandini S, Battaglia A, Alfieri S, Di Giacomo AM, Giannarelli D, et al. Baseline neutrophil-to-lymphocyte ratio is associated with outcome of ipilimumab-treated metastatic melanoma patients. British Journal of Cancer. 2015;112:1904-10.
[53] Ferrucci PF, Gandini S, Cocorocchio E, Pala L, Baldini F, Mosconi M, et al. Baseline relative eosinophil count as a predictive biomarker for ipilimumab treatment in advanced melanoma. Oncotarget. 2017;8:79809-15.
[54] Flem-Karlsen K, Nyakas M, Farstad IN, McFadden E, Wernhoff P, Jacobsen KD, et al. Soluble AXL as a marker of disease progression and survival in melanoma. PLoS One. 2020;15:e0227187.
[55] Forschner A, Battke F, Hadaschik D, Schulze M, Weißgraeber S, Han CT, et al. Tumor mutation burden and circulating tumor DNA in combined CTLA-4 and PD-1 antibody therapy in metastatic melanoma - results of a prospective biomarker study. J Immunother Cancer. 2019;7:180.
[56] Frankel AE, Coughlin LA, Kim J, Froehlich TW, Xie Y, Frenkel EP, et al. Metagenomic Shotgun Sequencing and Unbiased Metabolomic Profiling Identify Specific Human Gut Microbiota and Metabolites Associated with Immune Checkpoint Therapy Efficacy in Melanoma Patients. Neoplasia. 2017;19:848-55.
[57] Fröhlich A, Loick S, Bawden EG, Fietz S, Dietrich J, Diekmann E, et al. Comprehensive analysis of tumor necrosis factor receptor TNFRSF9 (4-1BB) DNA methylation with regard to molecular and clinicopathological features, immune infiltrates, and response prediction to immunotherapy in melanoma. EBioMedicine. 2020;52:102647.
[58] Fujimura T, Sato Y, Tanita K, Kambayashi Y, Otsuka A, Fujisawa Y, et al. Serum Level of Soluble CD163 May Be a Predictive Marker of the Effectiveness of Nivolumab in Patients With Advanced Cutaneous Melanoma. Front Oncol. 2018;8:530.
[59] Fujimura T, Sato Y, Tanita K, Lyu C, Kambayashi Y, Amagai R, et al. Association of Baseline Serum Levels of CXCL5 With the Efficacy of Nivolumab in Advanced Melanoma. Front Med (Lausanne). 2019;6:86.
[60] Galore-Haskel G, Nemlich Y, Greenberg E, Ashkenazi S, Hakim M, Itzhaki O, et al. A novel immune resistance mechanism of melanoma cells controlled by the ADAR1 enzyme. Oncotarget. 2015;6:28999-9015.
[61] Gambichler T, Brown V, Steuke AK, Schmitz L, Stockfleth E, Susok L. Baseline laboratory parameters predicting clinical outcome in melanoma patients treated with ipilimumab: a single-centre analysis. J Eur Acad Dermatol Venereol. 2018;32:972-7.
[62] Gao J, Shi LZ, Zhao H, Chen J, Xiong L, He Q, et al. Loss of IFN-gamma Pathway Genes in Tumor Cells as a Mechanism of Resistance to Anti-CTLA-4 Therapy. Cell. 2016;167:397-404 e9.
[63] Gebhardt C, Sevko A, Jiang H, Lichtenberger R, Reith M, Tarnanidis K, et al. Myeloid Cells and Related Chronic Inflammatory Factors as Novel Predictive Markers in Melanoma Treatment with Ipilimumab. Clin Cancer Res. 2015;21:5453-9.

[64] Ghorani E, Rosenthal R, McGranahan N, Reading JL, Lynch M, Peggs KS, et al. Differential binding affinity of mutated peptides for MHC class I is a predictor of survival in advanced lung cancer and melanoma. Ann Oncol. 2018;29:271-9.
[65] Gide TN, Quek C, Menzies AM, Tasker AT, Shang P, Holst J, et al. Distinct Immune Cell Populations Define Response to Anti-PD-1 Monotherapy and Anti-PD-1/Anti-CTLA-4 Combined Therapy. Cancer Cell. 2019;35:238-55.e6.
[66] Goltz D, Gevensleben H, Vogt TJ, Dietrich J, Golletz C, Bootz F, et al. CTLA4 methylation predicts response to anti-PD-1 and anti-CTLA-4 immunotherapy in melanoma patients. JCI Insight. 2018;3.
[67] Gopalakrishnan V, Spencer CN, Nezi L, Reuben A, Andrews MC, Karpinets TV, et al. Gut microbiome modulates response to anti-PD-1 immunotherapy in melanoma patients. Science. 2018;359:97-103.
[68] Graves M, CelliMarchett G, van Zyl B, Tang D, Vilain RE, van der Westhuizen A, et al. Monitoring Patient Response to Pembrolizumab With Peripheral Blood Exhaustion Marker Profiles. Front Med (Lausanne). 2019;6:113.
[69] Hamid O, Schmidt H, Nissan A, Ridolfi L, Aamdal S, Hansson J, et al. A prospective phase II trial exploring the association between tumor microenvironment biomarkers and clinical activity of ipilimumab in advanced melanoma. Journal of Translational Medicine. 2011;9.
[70] Hamid O, Robert C, Daud A, Hodi FS, Hwu WJ, Kefford R, et al. Five-year survival outcomes for patients with advanced melanoma treated with pembrolizumab in KEYNOTE-001. Ann Oncol. 2019;30:582-8.
[71] Heidelberger V, Goldwasser F, Kramkimel N, Jouinot A, Franck N, Arrondeau J, et al. Clinical parameters associated with anti-programmed death-1 (PD-1) inhibitors-induced tumor response in melanoma patients. Invest New Drugs. 2017;35:842-7.
[72] Hogan SA, Courtier A, Cheng PF, Jaberg-Bentele NF, Goldinger SM, Manuel M, et al. Peripheral Blood TCR Repertoire Profiling May Facilitate Patient Stratification for Immunotherapy against Melanoma. Cancer Immunol Res. 2019;7:77-85.
[73] Hong X, Sullivan RJ, Kalinich M, Kwan TT, Giobbie-Hurder A, Pan S, et al. Molecular signatures of circulating melanoma cells for monitoring early response to immune checkpoint therapy. Proc Natl Acad Sci U S A. 2018;115:2467-72.
[74] Huang AC, Postow MA, Orlowski RJ, Mick R, Bengsch B, Manne S, et al. T-cell invigoration to tumour burden ratio associated with anti-PD-1 response. Nature. 2017;545:60-5.
[75] Hugo W, Zaretsky JM, Sun L, Song C, Moreno BH, Hu-Lieskovan S, et al. Genomic and Transcriptomic Features of Response to Anti-PD-1 Therapy in Metastatic Melanoma. Cell. 2016;165:35-44.
[76] Iivanainen S, Ahvonen J, Knuuttila A, Tiainen S, Koivunen JP. Elevated CRP levels indicate poor progression-free and overall survival on cancer patients treated with PD-1 inhibitors. ESMO Open. 2019;4.
[77] Indini A, Di Guardo L, Cimminiello C, Prisciandaro M, Randon G, De Braud F, et al. Developing a score system to predict therapeutic outcomes to anti-PD-1 immunotherapy in metastatic melanoma. Tumori. 2019;105:465-73.
[78] Inoue H, Park JH, Kiyotani K, Zewde M, Miyashita A, Jinnin M, et al. Intratumoral expression levels of PD-L1, GZMA, and HLA-A along with oligoclonal T cell expansion associate with response to nivolumab in metastatic melanoma. Oncoimmunology. 2016;5:e1204507.
[79] Jacquelot N, Roberti MP, Enot DP, Rusakiewicz S, Ternès N, Jegou S, et al. Predictors of responses to immune checkpoint blockade in advanced melanoma. Nat Commun. 2017;8:592.
[80] Jensen C, Madsen DH, Hansen M, Schmidt H, Svane IM, Karsdal MA, et al. Non-invasive biomarkers derived from the extracellular matrix associate with response to immune checkpoint blockade (anti-CTLA-4) in metastatic melanoma patients. J Immunother Cancer. 2018;6:152.
[81] Ji RR, Chasalow SD, Wang L, Hamid O, Schmidt H, Cogswell J, et al. An immune-active tumor microenvironment favors clinical response to ipilimumab. Cancer Immunology, Immunotherapy. 2012;61:1019-31.
[82] Jiang P, Gu S, Pan D, Fu J, Sahu A, Hu X, et al. Signatures of T cell dysfunction and exclusion predict cancer immunotherapy response. Nat Med. 2018;24:1550-8.
[83] Johnson DB, Bordeaux J, Kim JY, Vaupel C, Rimm DL, Ho TH, et al. Quantitative Spatial Profiling of PD-1/PD-L1 Interaction and HLA-DR/IDO-1 Predicts Improved Outcomes of Anti-PD-1 Therapies in Metastatic Melanoma. Clin Cancer Res. 2018;24:5250-60.
[84] Johnson DB, Estrada MV, Salgado R, Sanchez V, Doxie DB, Opalenik SR, et al. Melanoma-specific MHC-II expression represents a tumour-autonomous phenotype and predicts response to anti-PD-1/PD-L1 therapy. Nat Commun. 2016;7:10582.
[85] Johnson DB, Frampton GM, Rioth MJ, Yusko E, Xu Y, Guo X, et al. Targeted Next Generation Sequencing Identifies Markers of Response to PD-1 Blockade. Cancer Immunol Res. 2016;4:959-67.
[86] Kalaora S, Lee JS, Barnea E, Levy R, Greenberg P, Alon M, et al. Immunoproteasome expression is associated with better prognosis and response to checkpoint therapies in melanoma. Nat Commun. 2020;11:896.

[87] Karachaliou N, Gonzalez-Cao M, Crespo G, Drozdowskyj A, Aldeguer E, Gimenez-Capitan A, et al. Interferon gamma, an important marker of response to immune checkpoint blockade in non-small cell lung cancer and melanoma patients. Ther Adv Med Oncol. 2018;10:1758834017749748.
[88] Kasanen H, Hernberg M, Mäkelä S, Brück O, Juteau S, Kohtamäki L, et al. Age-associated changes in the immune system may influence the response to anti-PD1 therapy in metastatic melanoma patients. Cancer Immunol Immunother. 2020;69:717-30.
[89] Kelderman S, Heemskerk B, Van Tinteren H, Van Den Brom RRH, Hospers GAP, Van Den Eertwegh AJM, et al. Lactate dehydrogenase as a selection criterion for ipilimumab treatment in metastatic melanoma. Cancer Immunology, Immunotherapy. 2014;63:449-58.
[90] Keller L, Guibert N, Casanova A, Brayer S, Farella M, Delaunay M, et al. Early Circulating Tumour DNA Variations Predict Tumour Response in Melanoma Patients Treated with Immunotherapy. Acta Derm Venereol. 2019;99:206-10.
[91] Khattak MA, Reid A, Freeman J, Pereira M, McEvoy A, Lo J, et al. PD-L1 Expression on Circulating Tumor Cells May Be Predictive of Response to Pembrolizumab in Advanced Melanoma: Results from a Pilot Study. Oncologist. 2020;25:e520-e7.
[92] Khoja L, Atenafu EG, Templeton A, Qye Y, Chappell MA, Saibil S, et al. The full blood count as a biomarker of outcome and toxicity in ipilimumab-treated cutaneous metastatic melanoma. Cancer Medicine. 2016;5:2792-9.
[93] Kirchberger MC, Ugurel S, Mangana J, Heppt MV, Eigentler TK, Berking C, et al. MEK inhibition may increase survival of NRAS-mutated melanoma patients treated with checkpoint blockade: Results of a retrospective multicentre analysis of 364 patients. Eur J Cancer. 2018;98:10-6.
[94] Kondo T, Nomura M, Otsuka A, Nonomura Y, Kaku Y, Matsumoto S, et al. Predicting marker for early progression in unresectable melanoma treated with nivolumab. Int J Clin Oncol. 2019;24:323-7.
[95] Krieg C, Nowicka M, Guglietta S, Schindler S, Hartmann FJ, Weber LM, et al. High-dimensional single-cell analysis predicts response to anti-PD-1 immunotherapy. Nat Med. 2018;24:144-53.
[96] Kubo Y, Fukushima S, Inamori Y, Tsuruta M, Egashira S, Yamada-Kanazawa S, et al. Serum concentrations of HGF are correlated with response to anti-PD-1 antibody therapy in patients with metastatic melanoma. J Dermatol Sci. 2019;93:33-40.
[97] Kumpers C, Jokic M, Haase O, Offermann A, Vogel W, Gratz V, et al. Immune Cell Infiltration of the Primary Tumor, Not PD-L1 Status, Is Associated With Improved Response to Checkpoint Inhibition in Metastatic Melanoma. Frontiers in Medicine. 2019;6:11.
[98] Lee J, Lee SJ, Kim K, Kim ST, Jang KT, Lee J. Comprehensive molecular and clinical characterization of Asian melanoma patients treated with anti-PD-1 antibody. BMC Cancer. 2019;19:805.
[99] Lee JH, Long GV, Boyd S, Lo S, Menzies AM, Tembe V, et al. Circulating tumour DNA predicts response to anti-PD1 antibodies in metastatic melanoma. Ann Oncol. 2017;28:1130-6.
[100] Li H, Bullock K, Gurjao C, Braun D, Shukla SA, Bossé D, et al. Metabolomic adaptations and correlates of survival to immune checkpoint blockade. Nat Commun. 2019;10:4346.
[101] Liu L, Bai X, Wang J, Tang XR, Wu DH, Du SS, et al. Combination of TMB and CNA Stratifies Prognostic and Predictive Responses to Immunotherapy Across Metastatic Cancer. Clin Cancer Res. 2019;25:7413-23.
[102] Łuksza M, Riaz N, Makarov V, Balachandran VP, Hellmann MD, Solovyov A, et al. A neoantigen fitness model predicts tumour response to checkpoint blockade immunotherapy. Nature. 2017;551:517-20.
[103] Maccalli C, Giannarelli D, Chiarucci C, Cutaia O, Giacobini G, Hendrickx W, et al. Soluble NKG2D ligands are biomarkers associated with the clinical outcome to immune checkpoint blockade therapy of metastatic melanoma patients. Oncoimmunology. 2017;6:10.
[104] Madonna G, Ballesteros-Merino C, Feng Z, Bifulco C, Capone M, Giannarelli D, et al. PD-L1 expression with immune-infiltrate evaluation and outcome prediction in melanoma patients treated with ipilimumab. Oncoimmunology. 2018;7:e1405206.
[105] Martens A, Wistuba-Hamprecht K, Geukes Foppen M, Yuan J, Postow MA, Wong P, et al. Baseline Peripheral Blood Biomarkers Associated with Clinical Outcome of Advanced Melanoma Patients Treated with Ipilimumab. Clin Cancer Res. 2016;22:2908-18.
[106] Martens A, Wistuba-Hamprecht K, Yuan J, Postow MA, Wong P, Capone M, et al. Increases in Absolute Lymphocytes and Circulating CD4+ and CD8+ T Cells Are Associated with Positive Clinical Outcome of Melanoma Patients Treated with Ipilimumab. Clin Cancer Res. 2016;22:4848-58.
[107] Mastracci L, Fontana V, Queirolo P, Carosio R, Grillo F, Morabito A, et al. Response to ipilimumab therapy in metastatic melanoma patients: potential relevance of CTLA-4(+) tumor infiltrating lymphocytes and their in situ localization. Cancer Immunol Immunother. 2020;69:653-62.
[108] Matson V, Fessler J, Bao R, Chongsuwat T, Zha Y, Alegre ML, et al. The commensal microbiome is associated with anti-PD-1 efficacy in metastatic melanoma patients. Science. 2018;359:104-8.

[109] McGranahan N, Furness AJ, Rosenthal R, Ramskov S, Lyngaa R, Saini SK, et al. Clonal neoantigens elicit T cell immunoreactivity and sensitivity to immune checkpoint blockade. Science. 2016;351:1463-9.
[110] Meyer C, Cagnon L, Costa-Nunes CM, Baumgaertner P, Montandon N, Leyvraz L, et al. Frequencies of circulating MDSC correlate with clinical outcome of melanoma patients treated with ipilimumab. Cancer Immunology, Immunotherapy. 2014;63:247-57.
[111] Minowa T, Kato J, Hida T, Horimoto K, Sato S, Sawada M, et al. Prognostic role of neutrophil to lymphocyte ratio in advanced melanoma treated with anti-programmed death-1 therapy. J Dermatol. 2018;45:e250-e1.
[112] Moreira A, Leisgang W, Schuler G, Heinzerling L. Eosinophilic count as a biomarker for prognosis of melanoma patients and its importance in the response to immunotherapy. Immunotherapy. 2017;9:115-21.
[113] Morello S, Capone M, Sorrentino C, Giannarelli D, Madonna G, Mallardo D, et al. Soluble CD73 as biomarker in patients with metastatic melanoma patients treated with nivolumab. J Transl Med. 2017;15:244.
[114] Morrison C, Pabla S, Conroy JM, Nesline MK, Glenn ST, Dressman D, et al. Predicting response to checkpoint inhibitors in melanoma beyond PD-L1 and mutational burden. J Immunother Cancer. 2018;6:32.
[115] Muto Y, Kitano S, Tsutsumida A, Namikawa K, Takahashi A, Nakamura Y, et al. Investigation of clinical factors associated with longer overall survival in advanced melanoma patients treated with sequential ipilimumab. J Dermatol. 2019;46:498-506.
[116] Nakamura Y, Kitano S, Takahashi A, Tsutsumida A, Namikawa K, Tanese K, et al. Nivolumab for advanced melanoma: pretreatment prognostic factors and early outcome markers during therapy. Oncotarget. 2016;7:77404-15.
[117] Nakamura Y, Tanaka R, Maruyama H, Ishitsuka Y, Okiyama N, Watanabe R, et al. Correlation between blood cell count and outcome of melanoma patients treated with anti-PD-1 antibodies. Japanese Journal of Clinical Oncology. 2019;49:431-7.
[118] Nie RC, Yuan SQ, Wang Y, Chen YB, Cai YY, Chen S, et al. Robust immunoscore model to predict the response to anti-PD1 therapy in melanoma. Aging-US. 2019;11:11576-90.
[119] Nonomura Y, Otsuka A, Nakashima C, Seidel JA, Kitoh A, Dainichi T, et al. Peripheral blood Th9 cells are a possible pharmacodynamic biomarker of nivolumab treatment efficacy in metastatic melanoma patients. Oncoimmunology. 2016;5:e1248327.
[120] Nosrati A, Tsai KK, Goldinger SM, Tumeh P, Grimes B, Loo K, et al. Evaluation of clinicopathological factors in PD-1 response: derivation and validation of a prediction scale for response to PD-1 monotherapy. Br J Cancer. 2017;116:1141-7.
[121] Okuhira H, Yamamoto Y, Inaba Y, Kunimoto K, Mikita N, Ikeda T, et al. Prognostic factors of daily blood examination for advanced melanoma patients treated with nivolumab. Biosci Trends. 2018;12:412-8.
[122] Pan M, Alavi M, Herrinton LJ. Association of Inflammatory Markers with Disease Progression in Patients with Metastatic Melanoma Treated with Immune Checkpoint Inhibitors. Perm J. 2018;22:17-149.
[123] Perrone F, Minari R, Bersanelli M, Bordi P, Tiseo M, Favari E, et al. The Prognostic Role of High Blood Cholesterol in Advanced Cancer Patients Treated With Immune Checkpoint Inhibitors. J Immunother. 2020;43:196-203.
[124] Pirozyan MR, McGuire HM, Emran AA, Tseng HY, Tiffen JC, Lee JH, et al. Pretreatment Innate Cell Populations and CD4 T Cells in Blood Are Associated With Response to Immune Checkpoint Blockade in Melanoma Patients. Front Immunol. 2020;11:372.
[125] Pistillo MP, Fontana V, Morabito A, Dozin B, Laurent S, Carosio R, et al. Soluble CTLA-4 as a favorable predictive biomarker in metastatic melanoma patients treated with ipilimumab: an Italian melanoma intergroup study. Cancer Immunol Immunother. 2019;68:97-107.
[126] Postow MA, Manuel M, Wong P, Yuan J, Dong Z, Liu C, et al. Peripheral T cell receptor diversity is associated with clinical outcomes following ipilimumab treatment in metastatic melanoma. J Immunother Cancer. 2015;3:23.
[127] Retseck J, Nasr A, Lin Y, Lin H, Mendiratta P, Butterfield LH, et al. Long term impact of CTLA4 blockade immunotherapy on regulatory and effector immune responses in patients with melanoma. J Transl Med. 2018;16:184.
[128] Riaz N HJ, Kendall SM, Makarov V, Walsh LA, Desrichard A, Weinhold N, Chan TA. Recurrent SERPINB3 and SERPINB4 mutations in patients who respond to anti-CTLA4 immunotherapy. Nat Genet. 2016;48:1327-9.
[129] Riaz N, Havel JJ, Makarov V, Desrichard A, Urba WJ, Sims JS, et al. Tumor and Microenvironment Evolution during Immunotherapy with Nivolumab. Cell. 2017;171:934-49.e16.
[130] Ribas A, Shin DS, Zaretsky J, Frederiksen J, Cornish A, Avramis E, et al. PD-1 Blockade Expands Intratumoral Memory T Cells. Cancer Immunol Res. 2016;4:194-203.
[131] Rodig SJ, Gusenleitner D, Jackson DG, Gjini E, Giobbie-Hurder A, Jin C, et al. MHC proteins confer differential sensitivity to CTLA-4 and PD-1 blockade in untreated metastatic melanoma. Sci Transl Med. 2018;10.

[132] Roh W, Chen PL, Reuben A, Spencer CN, Prieto PA, Miller JP, et al. Integrated molecular analysis of tumor biopsies on sequential CTLA-4 and PD-1 blockade reveals markers of response and resistance. Sci Transl Med. 2017;9.
[133] Rosner S, Kwong E, Shoushtari AN, Friedman CF, Betof AS, Brady MS, et al. Peripheral blood clinical laboratory variables associated with outcomes following combination nivolumab and ipilimumab immunotherapy in melanoma. Cancer Med. 2018;7:690-7.
[134] Sade-Feldman M, Kanterman J, Klieger Y, Ish-Shalom E, Olga M, Saragovi A, et al. Clinical significance of circulating CD33⁺ CD11bHLA-DR myeloid cells in patients with stage IV melanoma treated with ipilimumab. Clinical Cancer Research. 2016;22:5661-72.
[135] Sanmamed MF, Perez-Gracia JL, Schalper KA, Fusco JP, Gonzalez A, Rodriguez-Ruiz ME, et al. Changes in serum interleukin-8 (IL-8) levels reflect and predict response to anti-PD-1 treatment in melanoma and non-small-cell lung cancer patients. Ann Oncol. 2017;28:1988-95.
[136] Seremet T, Jansen Y, Planken S, Njimi H, Delaunoy M, El Housni H, et al. Undetectable circulating tumor DNA (ctDNA) levels correlate with favorable outcome in metastatic melanoma patients treated with anti-PD1 therapy. J Transl Med. 2019;17:303.
[137] Shukla SA, Bachireddy P, Schilling B, Galonska C, Zhan Q, Bango C, et al. Cancer-Germline Antigen Expression Discriminates Clinical Outcome to CTLA-4 Blockade. Cell. 2018;173:624-33.e8.
[138] Simeone E, Gentilcore G, Giannarelli D, Grimaldi AM, Caraco C, Curvietto M, et al. Immunological and biological changes during ipilimumab treatment and their potential correlation with clinical response and survival in patients with advanced melanoma. Cancer Immunology, Immunotherapy. 2014;63:675-83.
[139] Simon S, Vignard V, Varey E, Parrot T, Knol AC, Khammari A, et al. Emergence of High-Avidity Melan-A-Specific Clonotypes as a Reflection of Anti-PD-1 Clinical Efficacy. Cancer Res. 2017;77:7083-93.
[140] Simon SCS, Hu X, Panten J, Grees M, Renders S, Thomas D, et al. Eosinophil accumulation predicts response to melanoma treatment with immune checkpoint inhibitors. Oncoimmunology. 2020;9:1727116.
[141] Smithy JW, Moore LM, Pelekanou V, Rehman J, Gaule P, Wong PF, et al. Nuclear IRF-1 expression as a mechanism to assess "Capability" to express PD-L1 and response to PD-1 therapy in metastatic melanoma. J Immunother Cancer. 2017;5:25.
[142] Snyder A, Makarov V, Merghoub T, Yuan J, Zaretsky JM, Desrichard A, et al. Genetic basis for clinical response to CTLA-4 blockade in melanoma. N Engl J Med. 2014;371:2189-99.
[143] Subrahmanyam PB, Dong Z, Gusenleitner D, Giobbie-Hurder A, Severgnini M, Zhou J, et al. Distinct predictive biomarker candidates for response to anti-CTLA-4 and anti-PD-1 immunotherapy in melanoma patients. J Immunother Cancer. 2018;6:18.
[144] Tarhini AA, Edington H, Butterfield LH, Lin Y, Shuai Y, Tawbi H, et al. Immune monitoring of the circulation and the tumor microenvironment in patients with regionally advanced melanoma receiving neoadjuvant ipilimumab. PLoS One. 2014;9:e87705.
[145] Tarhini AA, Zahoor H, Lin Y, Malhotra U, Sander C, Butterfield LH, et al. Baseline circulating IL-17 predicts toxicity while TGF-beta1 and IL-10 are prognostic of relapse in ipilimumab neoadjuvant therapy of melanoma. J Immunother Cancer. 2015;3:39.
[146] Tarhini AA, Lin Y, Lin HM, Vallabhaneni P, Sander C, LaFramboise W, et al. Expression profiles of immune-related genes are associated with neoadjuvant ipilimumab clinical benefit. Oncoimmunology. 2017;6:e1231291.
[147] Tietze JK, Angelova D, Heppt MV, Reinholz M, Murphy WJ, Spannagl M, et al. The proportion of circulating CD45RO(+)CD8(+) memory T cells is correlated with clinical response in melanoma patients treated with ipilimumab. Eur J Cancer. 2017;75:268-79.
[148] Tietze JK, Angelova D, Heppt MV, Ruzicka T, Berking C. Low baseline levels of NK cells may predict a positive response to ipilimumab in melanoma therapy. Exp Dermatol. 2017;26:622-9.
[149] Topalian SL, Hodi FS, Brahmer JR, Gettinger SN, Smith DC, McDermott DF, et al. Safety, activity, and immune correlates of anti-PD-1 antibody in cancer. N Engl J Med. 2012;366:2443-54.
[150] Tucci M, Passarelli A, Mannavola F, Stucci LS, Ascierto PA, Capone M, et al. Serum exosomes as predictors of clinical response to ipilimumab in metastatic melanoma. Oncoimmunology. 2018;7:e1387706.
[151] Tumeh PC, Harview CL, Yearley JH, Shintaku IP, Taylor EJ, Robert L, et al. PD-1 blockade induces responses by inhibiting adaptive immune resistance. Nature. 2014;515:568-71.
[152] Urun Y, Yasar HA, Turna H, Esin E, Sedef AM, Alkan A, et al. Prognostic factors for survival in patients with metastatic malign melanoma treated with ipilimumab: Turkish Oncology Group study. J Oncol Pharm Pract. 2019;25:1658-64.
[153] Uryvaev A, Passhak M, Hershkovits D, Sabo E, Bar-Sela G. The role of tumor-infiltrating lymphocytes (TILs) as a predictive biomarker of response to anti-PD1 therapy in patients with metastatic non-small cell lung cancer or metastatic melanoma. Med Oncol. 2018;35:25.

[154] Valpione S, Martinoli C, Fava P, Mocellin S, Campana LG, Quaglino P, et al. Personalised medicine: Development and external validation of a prognostic model for metastatic melanoma patients treated with ipilimumab. Eur J Cancer. 2015;51:2086-94.
[155] Van Allen EM, Miao D, Schilling B, Shukla SA, Blank C, Zimmer L, et al. Genomic correlates of response to CTLA-4 blockade in metastatic melanoma. Science. 2015;350:207-11.
[156] Varn FS, Wang Y, Cheng C. A B cell-derived gene expression signature associates with an immunologically active tumor microenvironment and response to immune checkpoint blockade therapy. Oncoimmunology. 2019;8:e1513440.
[157] Vilain RE, Menzies AM, Wilmott JS, Kakavand H, Madore J, Guminski A, et al. Dynamic Changes in PD-L1 Expression and Immune Infiltrates Early During Treatment Predict Response to PD-1 Blockade in Melanoma. Clin Cancer Res. 2017;23:5024-33.
[158] Wagner NB, Forschner A, Leiter U, Garbe C, Eigentler TK. S100B and LDH as early prognostic markers for response and overall survival in melanoma patients treated with anti-PD-1 or combined anti-PD-1 plus anti-CTLA-4 antibodies. Br J Cancer. 2018;119:339-46.
[159] Wagner NB, Weide B, Gries M, Reith M, Tarnanidis K, Schuermans V, et al. Tumor microenvironment-derived S100A8/A9 is a novel prognostic biomarker for advanced melanoma patients and during immunotherapy with anti-PD-1 antibodies. J Immunother Cancer. 2019;7:343.
[160] Weber JS, Sznol M, Sullivan RJ, Blackmon S, Boland G, Kluger HM, et al. A Serum Protein Signature Associated with Outcome after Anti-PD-1 Therapy in Metastatic Melanoma. Cancer Immunol Res. 2018;6:79-86.
[161] Weber J, Gibney G, Kudchadkar R, Yu B, Cheng P, Martinez AJ, et al. Phase I/II Study of Metastatic Melanoma Patients Treated with Nivolumab Who Had Progressed after Ipilimumab. Cancer Immunol Res. 2016;4:345-53.
[162] Weide B, Martens A, Hassel JC, Berking C, Postow MA, Bisschop K, et al. Baseline Biomarkers for Outcome of Melanoma Patients Treated with Pembrolizumab. Clin Cancer Res. 2016;22:5487-96.
[163] Wen X, Ding Y, Li J, Zhao J, Peng R, Li D, et al. The experience of immune checkpoint inhibitors in Chinese patients with metastatic melanoma: a retrospective case series. Cancer Immunol Immunother. 2017;66:1153-62.
[164] Wistuba-Hamprecht K, Martens A, Haehnel K, Geukes Foppen M, Yuan J, Postow MA, et al. Proportions of blood-borne Vδ1+ and Vδ2+ T-cells are associated with overall survival of melanoma patients treated with ipilimumab. Eur J Cancer. 2016;64:116-26.
[165] Wistuba-Hamprecht K, Martens A, Heubach F, Romano E, Geukes Foppen M, Yuan J, et al. Peripheral CD8 effector-memory type 1 T-cells correlate with outcome in ipilimumab-treated stage IV melanoma patients. European Journal of Cancer. 2017;73:61-70.
[166] Wong PF, Wei W, Gupta S, Smithy JW, Zelterman D, Kluger HM, et al. Multiplex quantitative analysis of cancer-associated fibroblasts and immunotherapy outcome in metastatic melanoma. J Immunother Cancer. 2019;7:194.
[167] Wong PF, Wei W, Smithy JW, Acs B, Toki MI, Blenman KRM, et al. Multiplex Quantitative Analysis of Tumor-Infiltrating Lymphocytes and Immunotherapy Outcome in Metastatic Melanoma. Clin Cancer Res. 2019;25:2442-9.
[168] Wood MA, Weeder BR, David JK, Nellore A, Thompson RF. Burden of tumor mutations, neoepitopes, and other variants are weak predictors of cancer immunotherapy response and overall survival. Genome Med. 2020;12:33.
[169] Woods DM, Laino AS, Winters A, Alexandre J, Freeman D, Rao V, et al. Nivolumab and ipilimumab are associated with distinct immune landscape changes and response-associated immunophenotypes. JCI Insight. 2020;5.
[170] Xiao W, Du N, Huang T, Guo J, Mo X, Yuan T, et al. TP53 Mutation as Potential Negative Predictor for Response of Anti-CTLA-4 Therapy in Metastatic Melanoma. EBioMedicine. 2018;32:119-24.
[171] Yamazaki N, Kiyohara Y, Uhara H, Iizuka H, Uehara J, Otsuka F, et al. Cytokine biomarkers to predict antitumor responses to nivolumab suggested in a phase 2 study for advanced melanoma. Cancer Sci. 2017;108:1022-31.
[172] Yoshida T, Ichikawa J, Giuroiu I, Laino AS, Hao Y, Krogsgaard M, et al. C reactive protein impairs adaptive immunity in immune cells of patients with melanoma. Journal for Immunotherapy of Cancer. 2020;8:13.
[173] Yuan J, Adamow M, Ginsberg BA, Rasalan TS, Ritter E, Gallardo HF, et al. Integrated NY-ESO-1 antibody and CD8+ T-cell responses correlate with clinical benefit in advanced melanoma patients treated with ipilimumab. Proc Natl Acad Sci U S A. 2011;108:16723-8.
[174] Yuan JD, Zhou J, Dong ZW, Tandon S, Kuk D, Panageas KS, et al. Pretreatment Serum VEGF Is Associated with Clinical Response and Overall Survival in Advanced Melanoma Patients Treated with Ipilimumab. Cancer Immunology Research. 2014;2:127-32.
[175] Yusko E, Vignali M, Wilson RK, Mardis ER, Hodi FS, Horak C, et al. Association of Tumor Microenvironment T-cell Repertoire and Mutational Load with Clinical Outcome after Sequential Checkpoint Blockade in Melanoma. Cancer Immunol Res. 2019;7:458-65.

[176] Zaragoza J, Caille A, Beneton N, Bens G, Christiann F, Maillard H, et al. High neutrophil to lymphocyte ratio measured before starting ipilimumab treatment is associated with reduced overall survival in patients with melanoma. British Journal of Dermatology. 2016;174:146-51.
[177] Zhou J, Mahoney KM, Giobbie-Hurder A, Zhao F, Lee S, Liao X, et al. Soluble PD-L1 as a Biomarker in Malignant Melanoma Treated with Checkpoint Blockade. Cancer Immunol Res. 2017;5:480-92.
